# Glutamine prevents acute kidney injury by modulating oxidative stress and apoptosis in tubular epithelial cells

**DOI:** 10.1172/jci.insight.163161

**Published:** 2022-11-08

**Authors:** Katharina Thomas, Lisa Zondler, Nadine Ludwig, Marina Kardell, Corinna Lüneburg, Katharina Henke, Sina Mersmann, Andreas Margraf, Tilmann Spieker, Tobias Tekath, Ana Velic, Richard Holtmeier, Juliane Hermann, Vera Jankowski, Melanie Meersch, Dietmar Vestweber, Martin Westphal, Johannes Roth, Michael A. Schäfers, John A. Kellum, Clifford A. Lowell, Jan Rossaint, Alexander Zarbock

**Affiliations:** 1 Department of Anesthesiology, Intensive Care and Pain Medicine, University Hospital Münster, Münster, Germany.; 2 Institute for Pathology, St. Franziskus Hospital Münster, Münster, Germany.; 3 Institute of Medical Informatics, University of Münster, Münster, Germany.; 4 Department of Quantitative Proteomics, University of Tübingen, Tübingen, Germany.; 5 Institute of Clinical Radiology, University Hospital Münster, Münster, Germany.; 6 Institute for Molecular Cardiovascular Research, RWTH Aachen University Hospital, Aachen, Germany.; 7 Max Planck Institute for Molecular Biomedicine, Münster, Germany.; 8 Fresenius Kabi AG, Bad Homburg, Germany.; 9 Institute for Immunology, University of Münster, Münster.; 10 European Institute for Molecular Imaging, University Hospital Münster, Münster, Germany.; 11 Center for Critical Care Nephrology, Department of Critical Care Medicine, University of Pittsburgh, Pittsburgh, Pennsylvania, USA.; 12 Department of Laboratory Medicine, University of California, San Francisco, San Francisco, California, USA.

**Keywords:** Immunology, Nephrology, Apoptosis, Cellular immune response, Mitochondria

## Abstract

Acute kidney injury (AKI) represents a common complication in critically ill patients that is associated with increased morbidity and mortality. In a murine AKI model induced by ischemia/reperfusion injury (IRI), we show that glutamine significantly decreases kidney damage and improves kidney function. We demonstrate that glutamine causes transcriptomic and proteomic reprogramming in murine renal tubular epithelial cells (TECs), resulting in decreased epithelial apoptosis, decreased neutrophil recruitment, and improved mitochondrial functionality and respiration provoked by an ameliorated oxidative phosphorylation. We identify the proteins glutamine gamma glutamyltransferase 2 (Tgm2) and apoptosis signal-regulating kinase (Ask1) as the major targets of glutamine in apoptotic signaling. Furthermore, the direct modulation of the Tgm2-HSP70 signalosome and reduced Ask1 activation resulted in decreased JNK activation, leading to diminished mitochondrial intrinsic apoptosis in TECs. Glutamine administration attenuated kidney damage in vivo during AKI and TEC viability in vitro under inflammatory or hypoxic conditions.

## Graphical abstract

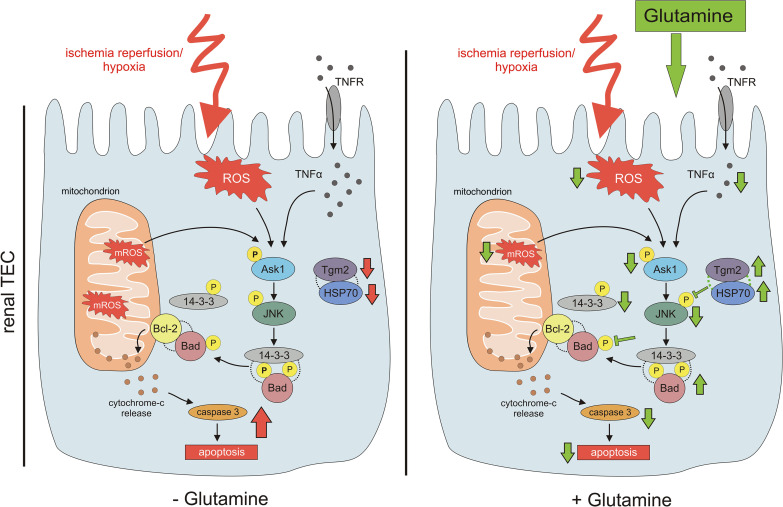

## Introduction

Acute kidney injury (AKI) may be caused by insults such as sepsis and nephrotoxins, as well as kidney ischemia/reperfusion injury (IRI). Tissue damage in AKI may occur in multiple parts of the nephron (tubular and glomerular). However, injury to renal tubular epithelial cells (TECs) is most critical for the degree of functional decline (1). TEC dysfunction represents the initiating event in AKI and directly correlates with tubular epithelial cell death in one or several tubular segments as a consequence of regulated necrosis, ferroptosis, and apoptosis (2, 3). Tubular cell loss initially provokes the secretion of inflammatory cytokines and chemokines by remaining TECs and resident leukocytes, thereby contributing to excess infiltration of neutrophils and other immune cells (4–6). Consequently, activation and recruitment of circulating neutrophils into the kidney are triggered; the amount of neutrophil infiltration correlates with the degree of tissue damage and the decline of renal function (7, 8).

Glutamine is the most abundant free amino acid in the body and is considered conditionally essential during prolonged periods of physical stress and inflammatory conditions such as infection and injury (9). This conditionally essential amino acid is required for protein biosynthesis but also for energy metabolism and reactive oxygen species (ROS) scavenging, which is of particular importance in high-turnover tissues and immune cells (10, 11). Reduced glutamine availability may result in impairment of biological mechanisms and physiological processes such as cell proliferation and survival. Renal TECs and immune cells have been reported to be the main consumers of glutamine (12). Glutamine supplementation has been found to suppress the production of inflammatory mediators, such as TNF-α or IL-6, by LPS-stimulated peripheral blood monocytes (13). Moreover, glutamine administration has been shown to prevent apoptosis of immortalized human kidney 2 (HK-2) cells through induction of heme oxygenase-1 via a p38MAPK-dependent pathway, which may play a key role in the cytoprotective effect of glutamine (14). Recent research has revealed that glutamine induces expression of heat shock proteins (HSPs), which may protect from cellular injury (15, 16). In addition, a previous report demonstrated that a single dose of glutamine administered after the initiation of sepsis downregulates the expression of high-motility group box-1–related (HMGB1-related) mediators and decreases renal oxidative stress (17). Finally, it has been reported that glutamine’s renal protective effect in nephrotoxic AKI is related to activation of HSP70, which mitigates inflammation and neutrophil infiltration (16).

Urinary biomarkers released by injured renal cells have been found to predict the development of clinical AKI (18, 19). Tissue inhibitor of metalloproteinase-2 (TIMP-2) and insulin-like growth factor binding protein 7 (IGFBP7) are both secreted under conditions of renal stress caused by inflammation, ischemia, oxidative stress, drugs, and toxins (20). These proteins are involved in G_1_ cell cycle arrest that occurs during cellular stress.

The aim of this study was to investigate the protective effect of glutamine application on the development of AKI in mice after renal IRI and to unveil the underlying molecular mechanisms. We show that systemic glutamine administration causes transcriptomic and proteomic reprogramming in renal TECs, which has a direct protective effect on renal tissue by interfering with proapoptotic signaling and inflammation. As a result, glutamine treatment reduces kidney injury by diminishing mitochondrial intrinsic apoptosis in TECs. Finally, we show that the systemic administration of glutamine substantially promotes the maintenance of renal tissue integrity and functionality in mice.

## Results

### Glutamine administration attenuates kidney damage and improves kidney function during IRI-induced AKI.

**Figure 1:**
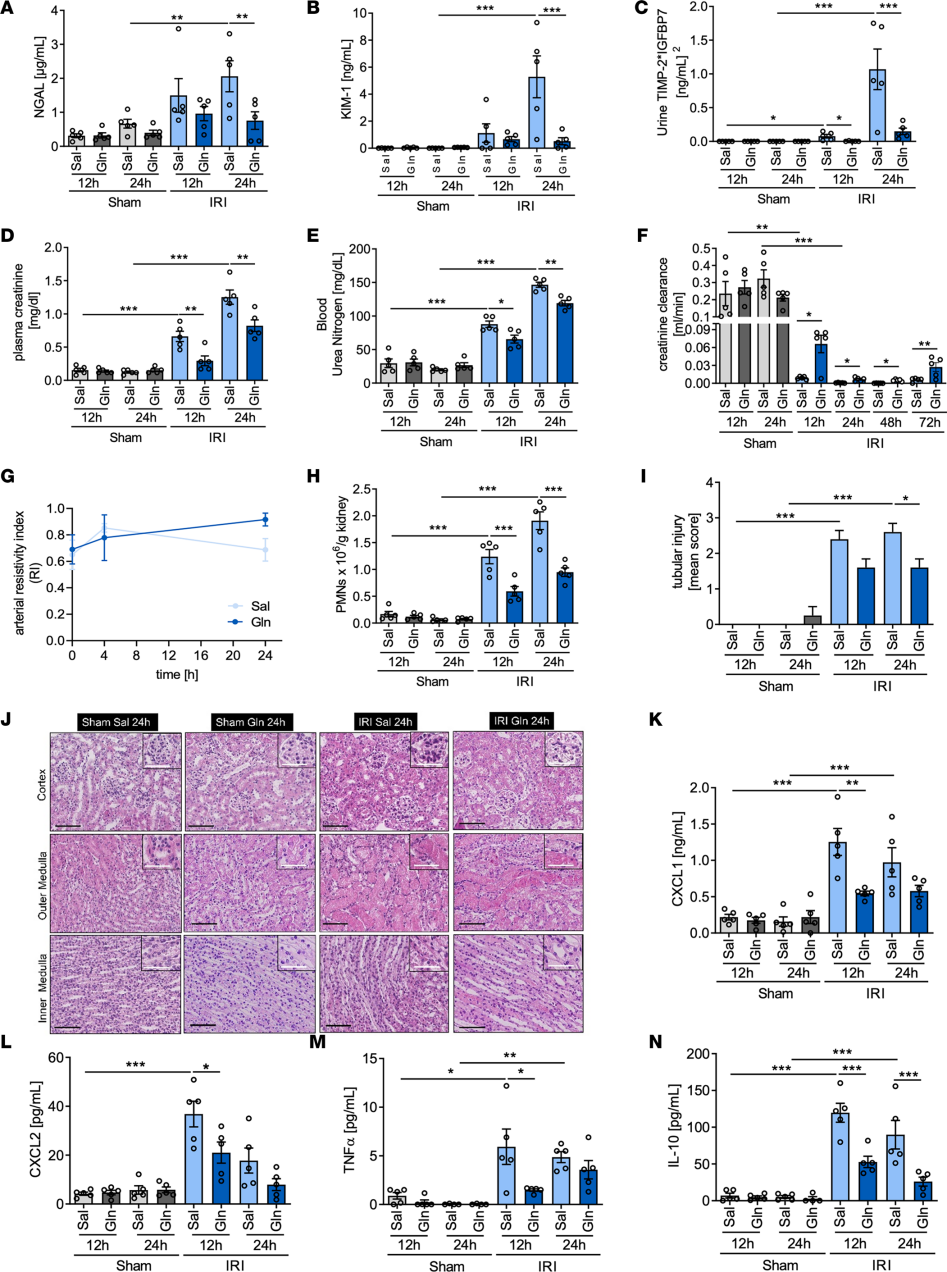
Glutamine administration attenuates kidney damage and improves kidney function during IRI-induced AKI. WT mice were subjected to sham or IRI surgery and received glutamine or saline 4 hours after reperfusion. The levels of urinary protein biomarkers specific for different parts of the kidney nephron were assessed by ELISA for all time points. NGAL (**A**, *n* = 5) represents the distal tubule function; KIM-1 (**B**, *n* = 5) represents the functionality of proximal tubules. TIMP-2 and IGFBP7 (**C**, *n* = 5) are biomarkers of G_1_ cell cycle arrest correlating with renal tubular cell stress. Plasma creatinine (**D**, *n* = 5) as well as blood urea nitrogen levels were measured (**E**, *n* = 5). Creatinine clearance was calculated 12 and 24 hours after the procedure (**F**, *n* = 5). Renal blood flow was analyzed in the *Arteria renalis* (*A*. *renalis*) at baseline conditions, 4 hours and 24 hours after IRI induction (**G**, *n* = 3; mean + SD). Neutrophil recruitment into the kidney was analyzed by flow cytometry (**H**, *n* = 5). After performance of H&E staining of paraffin-embedded sections 1 tissue section per mouse was scored to identify tissue damage (**I**, tubular injury score; **J**, representative H&E staining, *n* = 5). Black scale bar: 100 μm; white scale bar: 50 μm. The plasma levels of CXCL1 (**K**), CXCL2 (**L**), TNF-α (**M**), and IL-10 (**N**) were analyzed by ELISA (*n* = 5; mean ± SEM; 1-way ANOVA *P < 0.05; **P < 0.005; ***P < 0.001).

To investigate the effect of glutamine on AKI, we used a murine model of renal IRI (21). Mice were subjected to sham or renal IRI surgery and received glutamine or physiologic saline solution intravenously 4 hours after reperfusion. Urinary protein biomarkers of AKI, including neutrophil gelatinase-associated lipocalin (NGAL; Figure 1A) and kidney injury molecule-1 (KIM-1; Figure 1B), were increased after IRI induction but were significantly reduced in glutamine-treated mice. Similarly, levels of the renal stress biomarkers [TIMP‑2]*[IGFBP7] were increased in saline-treated IRI mice 12 hours after surgery and significantly attenuated by glutamine (Figure 1C). Plasma creatinine and blood urea nitrogen, measures of kidney dysfunction, were elevated in the saline group after IRI induction and significantly attenuated in the glutamine group (Figure 1, D and E). Glutamine administration also rescued creatinine clearance upon IRI (Figure 1F). The protective effect of glutamine was detectable for 72 hours after IRI induction, which represents the latest time point to be assessed before mice started dying (Figure 1F). To investigate whether glutamine protects the kidney after IRI by modulating the blood flow (22), we analyzed the renal arterial resistivity index at baseline conditions and 4 hours and 24 hours after renal IRI in the presence of either glutamine or saline (Figure 1G). We demonstrate that glutamine does not affect the renal arterial resistivity index, thereby indicating that the observed beneficial effects after glutamine treatment are not mediated by an increased renal blood flow but due to immunomodulation.

In addition, IRI-induced neutrophil recruitment into the kidneys was significantly reduced with glutamine (Figure 1H). Cell surface expression of activation markers on infiltrated neutrophils was not affected by glutamine (Supplemental Figure 1, A–E). Likewise, glutamine did not affect recruitment of other immune cells, such as macrophages, T cells, and dendritic cells (Supplemental Figure 1, F–I). To further investigate tubular injury after renal IRI, H&E staining of kidney sections was performed, and a investigator quantified tubular injury by using blinded protocol and a tubular injury score (23). In the control group, the tubular injury score significantly increased over time, and glutamine significantly reduced the score 24 hours after renal IRI (Figure 1, I and J, and Supplemental Figure 2A). Immune system activation and neutrophil recruitment upon reperfusion of ischemic tissues contribute to tissue damage and kidney dysfunction (21, 24). Therefore, we measured chemokine and cytokine levels as well as neutrophil recruitment into the kidneys after IRI induction. Both cytokine and chemokine levels (CXCL1, CXCL2, TNF-α, and IL-10) were decreased following therapeutic glutamine administration (Figure 1, K–N). These findings demonstrate that glutamine administration ameliorates both the global and local immune cell activation in renal IRI in vivo, thereby protecting tissue integrity and maintaining kidney function.

In order to obtain a better understanding of the time-dependent effects of glutamine, we investigated the dynamics of glutamine uptake by assessing glutamine levels in blood and urine after the administration of glutamine intravenously. We observed that 50% of the injected glutamine was quickly removed from the circulation (Supplemental Figure 2, B and C). To distinguish whether the observed glutamine-related protective effects were primarily achieved by direct action on renal TECs, we performed a reconstitution experiment. Mice were neutrophil-depleted, and glutamine was injected intravenously 1 hour before neutrophil reconstitution and sham or IRI procedure (Supplemental Figure 2D). In this model, glutamine treatment continued to significantly improve kidney function, suggesting that its effect was primarily on TECs (or other renal cells) rather than on neutrophils directly (Supplemental Figure 2, E and F).

### Glutamine affects reduction-oxidation capacity, NAD metabolism, and apoptotic processes.

**Figure 2:**
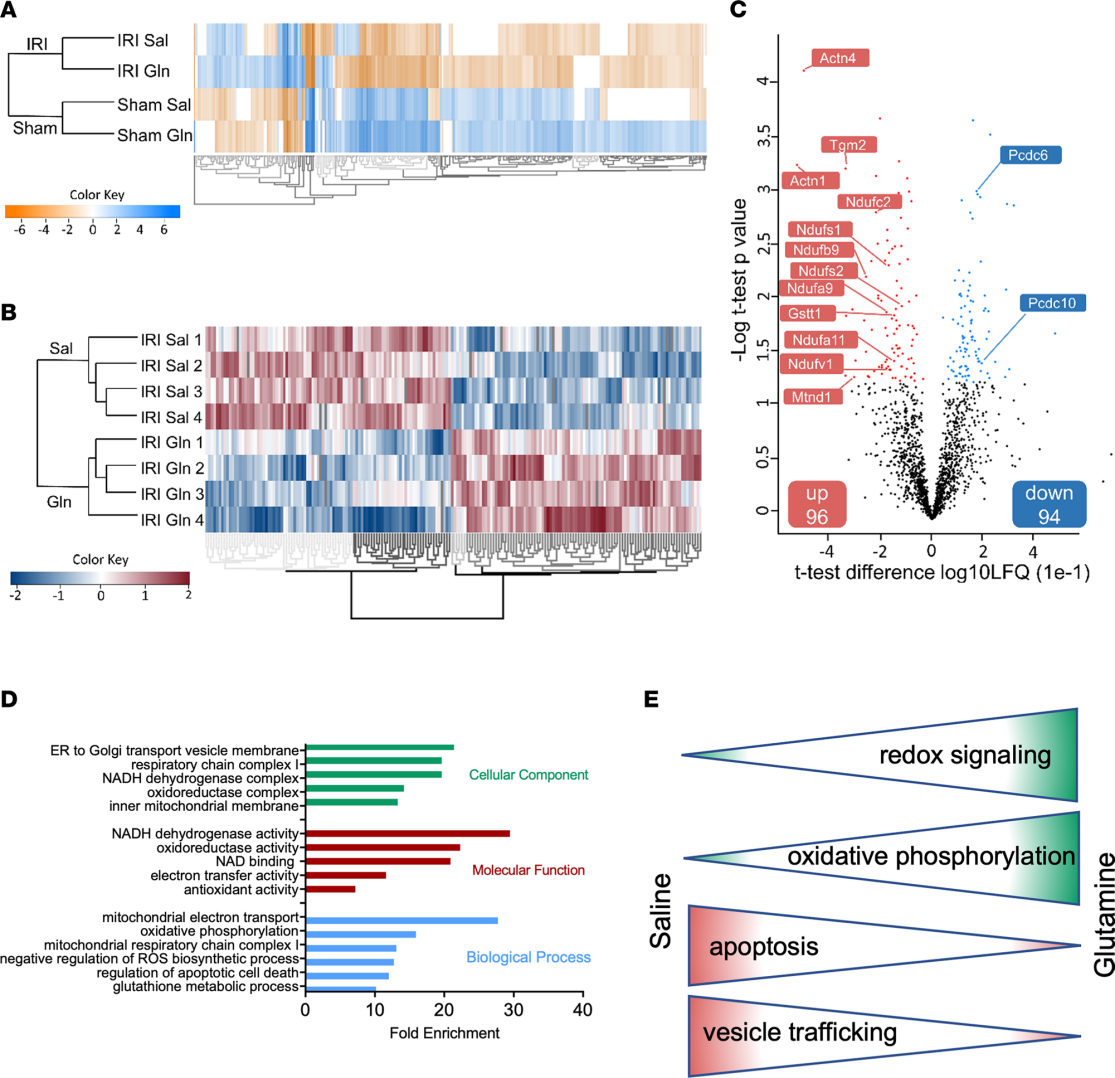
Glutamine affects reduction-oxidation capacity, NAD metabolism, and apoptotic processes. WT mice were subjected to sham or IRI surgery and received glutamine or saline 15 minutes after reperfusion. Kidneys were collected and homogenized 24 hours after IRI induction. Mass spectrometric label-free quantification was performed in order to identify alteration in protein expression levels as a result of glutamine treatment. A hierarchical clustering heatmap indicates differentially expressed genes (rows) between the respective sham and IRI groups (**A**, *n* = 4). Further analysis of 4 kidney homogenates of glutamine-treated mice and saline-treated mice after IRI induction revealed the effect of glutamine on renal protein expression. Red indicates upregulation and blue indicates downregulation (**B**, *n* = 4). Volcano plots generated to compare glutamine versus saline treatment after IRI induction reveal 190 proteins to be significantly differentially expressed (**C**, *n* = 4). Among these proteins, 96 proteins were significantly elevated due to glutamine treatment (indicated by red dots), whereas 94 proteins were significantly decreased (indicated by blue dots). GO terms representing molecular function are presented in red, cellular component in green, and biological processes in blue (**D**; Fisher’s exact test, *P* value < 0.05; only significantly modulated GO terms are displayed). (**E**) Schematic illustration of signaling pathways in renal TECs affected by glutamine treatment.

To investigate the mechanisms by which glutamine treatment reduced renal tissue injury upon AKI induction, we performed label-free quantitative mass spectrometry proteomic analysis of kidney homogenates from saline- and glutamine-treated mice after IRI induction or sham surgery. Unbiased hierarchical clustering analysis clearly separated the 4 groups, whereby 2,784 proteins were detected (Figure 2A). Additionally, a deeper proteomic analysis of the 2 IRI-treated groups verified fundamental differences of protein levels in response to glutamine treatment (Figure 2B). Overall, proteomic analysis of kidney tissue from saline- or glutamine-treated, IRI-induced mice identified 190 significantly regulated proteins out of 2,609 detected, of which 96 proteins were upregulated and 94 were downregulated in kidney homogenates of glutamine-treated mice following IRI (Figure 2C and Supplemental Table 1). A gene ontology (GO) enrichment analysis of the involved signaling pathways revealed glutamine administration affected oxidative phosphorylation, apoptosis, and glutathione metabolic processes as well as NAD^+^ binding and antioxidant activity (Figure 2D). This was also supported by a network analysis (see scheme in Figure 2, D and E, and Supplemental Figure 3). Upregulated key proteins included several subunits of NADH dehydrogenase 1 and NADH ubiquinone oxidoreductase (complex 1 of the oxidative phosphorylation system; Ndufc2, Ndufs1, Ndufb9, Ndufa11, Ndufs2, Mtnd1) as well as glutathione-*S*-transferase theta 1 (Gstt1) (Figure 2C and Supplemental Figure 3A). Protein-glutamine gamma glutamyltransferase 2 (Tgm2), alpha-actinin-1 (Actn1), and alpha-actinin-4 (Actn4), which are involved in apoptosis and leukocyte transendothelial migration, as well as cell division control protein 42 homolog (Cdc42), were among the top upregulated proteins. Downregulated proteins included apoptosis-related molecules such as programmed cell death proteins 6 and 10 (Pdcd-10 and -6) and vesicle trafficking, e.g., Sec22b and Arcn1 (Figure 2C and Supplemental Figure 3B).

### Glutamine administration functionally attenuates renal tubular cell apoptosis.

**Figure 3:**
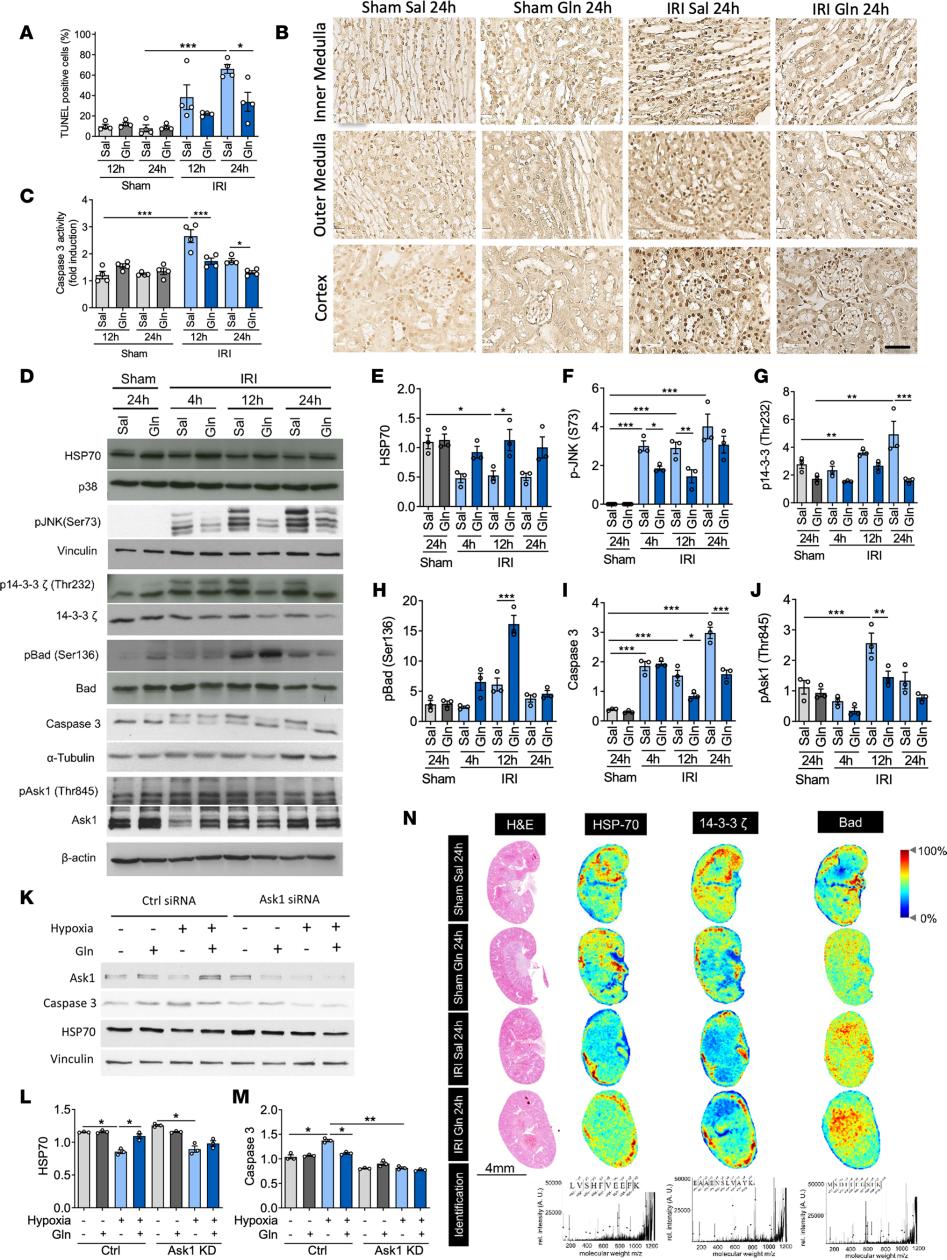
Glutamine administration functionally attenuates renal tubular cell apoptosis. WT mice were subjected to sham or IRI surgery and received glutamine or saline. Paraffin-embedded tissue sections were prepared and TUNEL staining was performed (**A**, TUNEL-positive cells [%]; **B**, representative TUNEL-stained cortex and medulla tissue) (*n* = 4; scale bar: 50 μm). Caspase-3 activity was detected in kidney lysates (**C**, *n* = 4). Western blotting was performed to assess the expression level of HSP70 (**D** and **E**), p-JNK (**D** and **F**), 14-3-3ζ and p–14‑3‑3ζ (Thr232) (**D** and **G**), Bad and p-Bad (Ser136) (**D** and **H**), caspase-3 (**D** and **I**), as well as apoptosis signal-regulating kinase (Ask1) and p-Ask1 (**D** and **J**). TECs were transfected with Ask1 siRNA and control siRNA using the Lipofectamine RNAiMAX Reagent. Knockdown efficiency determined by Western blot analysis was ~10% protein expression. Transfected TECs were treated with glutamine or saline, then subjected to hypoxia. TEC lysates were analyzed by Western blotting to assess the expression levels of HSP70 (**K** and **L**, *n* = 3) and caspase-3 (**K** and **M**, *n* = 3). MALDI imaging mass spectrometry (MALDI-IMS) of kidney sections was performed to analyze protein distribution of HSP70, 14-3-3ζ, and Bad (**N**). The scale represents the relative intensity of the protein (*m/z*, mass-to-charge ratio). Mean ± SEM, 1-way ANOVA *P < 0.05; **P < 0.005; ***P < 0.001.

Next, we sought to provide functional proof of our proteomic results. To explore the impact of glutamine on apoptosis in the kidney tissue after IRI, TUNEL-positive cells and caspase-3 activity were investigated in kidney tissue of sham and IRI-induced mice with or without glutamine treatment (Figure 3, A–C). The count of TUNEL-positive cells (Figure 3, A and B) and caspase-3 activity (Figure 3C) were significantly reduced by glutamine treatment after IRI induction. To provide a further mechanistic link of glutamine action on renal TEC apoptosis, immunoblotting of kidney lysates from mice treated with glutamine or saline was performed. Since the involvement of JNK in glutamine-dependent signaling has been reported to play a role in AKI (15), we analyzed the signaling intermediates in JNK signaling, including HSP70, phosphorylated JNK (p-JNK), p–Bcl-2 associated agonist of cell death (p-Bad), p-14-3-3ζ, and caspase-3, by biochemical assays and immunohistochemistry staining (Figure 3, D–I, and Supplemental Figure 4C). We found total HSP70 expression levels to be increased upon glutamine treatment in the kidney lysates of IRI-induced mice (Figure 3, D and E). Furthermore, while Bad phosphorylation was increased, the phosphorylation of JNK and 14-3-3ζ, as well as the expression of caspase-3, were decreased following glutamine treatment (Figure 3, D and F–I), suggesting that glutamine treatment decreases proapoptotic signaling. In addition, we investigated the impact of glutamine administration on Ask1 phosphorylation, which is known to activate JNK in response to an array of stresses, such as oxidative and inflammatory stress, thereby inducing apoptosis in various cells through mitochondria-dependent caspase-3 activation (25). As Ask1 might represent the link between oxidative stress and apoptosis, we analyzed Ask1 phosphorylation (Thr845) and indeed found glutamine to reduce IRI-induced Ask1 activation in kidneys (Figure 3, D and J). To unveil to role of Ask1 on apoptosis and especially glutamine-increased HSP70 expression, we induced an Ask1 knockdown (KD) in TECs by siRNA transfection and analyzed caspase-3 and HSP70 expression under normoxic and hypoxic conditions (Figure 3K). While HSP70 expression was not affected by Ask1 KD (Figure 3, K and L), caspase-3 expression was significantly decreased upon hypoxia of Ask1-KD cells (Figure 3, K and M).

To investigate the upstream components of Bad phosphorylation in kidney tissues following IRI induction, we investigated PI3K and Akt expression as well as Akt phosphorylation. While IRI-induced AKI did result in increased expression of PI3K and enhanced Akt activation 12 hours after IRI induction, glutamine treatment had no effect on these molecules (Supplemental Figure 4, A and B). This suggests that the increased Bad phosphorylation observed following glutamine treatment is more likely to be the result of increased JNK signaling rather than through modulation of PI3K pathways.

To visualize the spatial distribution of HSP70, Bad, and 14-3‑3ζ in kidney sections of glutamine- or saline-treated mice upon IRI induction, matrix-assisted laser desorption/ionization imaging mass spectrometry (MALDI-IMS) was performed (Figure 3N). Glutamine administration after IRI induction led to an increase of HSP70 expression both in the cortical and in the medullary regions of the kidney (Figure 3N). By contrast, neither 14-3-3ζ nor Bad distribution was affected by glutamine treatment after IRI induction.

These observations were additionally confirmed by immunohistochemistry (IHC) staining (Supplemental Figure 4C). Taken together, these data provide evidence that systemic glutamine administration prevents renal tubular cell apoptosis by improving antiapoptotic signaling via HSP70 and reduced Ask1 activation.

### Glutamine improves kidney function upon IRI by mediating Tgm2-HSP70 interaction.

**Figure 4:**
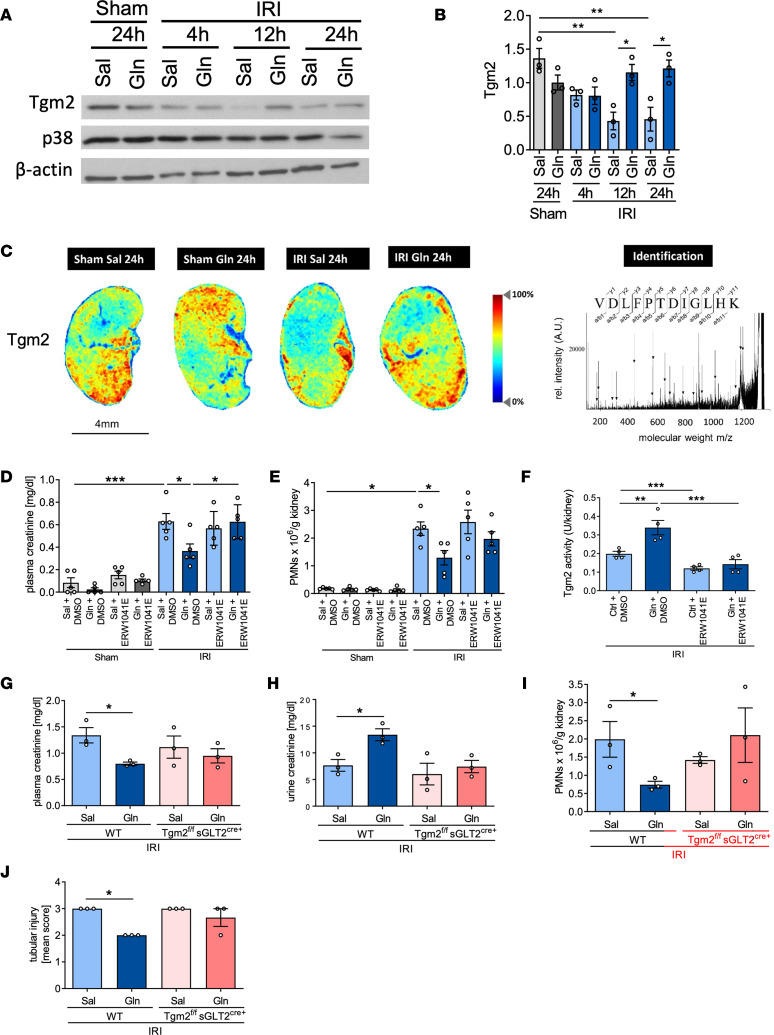
Glutamine improves kidney function upon IRI by modulating Tgm2-HSP70 interaction. WT mice were subjected to sham or IRI surgery and received glutamine or saline. Western blotting was performed to assess the expression of Tgm2 levels (**A** and **B**). MALDI-IMS of kidney sections was performed in order to analyze Tgm2 distribution (**C**). In addition to glutamine or saline exposure, mice obtained Tgm2 inhibitor ERW1041E or DMSO as vehicle control by an intravenous injection (**D**–**F**). Plasma creatinine levels (**D**, *n* = 5) and neutrophil recruitment into the kidney (**E**, *n* = 5) as well as Tgm2 activity in kidneys (**F**, *n* = 4) were determined 12 hours after IRI induction. WT and conditional Tgm2-KO mice (Tgm2^fl/fl^ sGLT2^cre+^) were subjected to IRI surgery. Plasma (**G**, *n* = 3) and urine creatinine (**H**, *n* = 3) as well as neutrophil recruitment (**I**, *n* = 3) and the renal tubular injury score (**J**, *n* = 3) were assessed 24 hours after IRI. Mean ± SEM, 1-way ANOVA *P < 0.05; **P < 0.005; ***P < 0.001.

As Tgm2 amounts in glutamine-treated kidneys were significantly elevated in our proteomic analysis, we verified increased Tgm2 expression levels in kidney lysates by Western blotting (Figure 4, A and B). MALDI-IMS demonstrated Tgm2 to be expressed in the cortical and the medullary structure of the kidney under baseline conditions (Figure 4C). While IRI induction led to a decreased Tgm2 distribution in both cortical and central regions of the kidney, glutamine treatment rescued Tgm2 expression in the medulla, and particularly in cortical areas (Figure 4C). This was additionally observed by IHC staining (Supplemental Figure 4C).

To further investigate the role of Tgm2 activity in glutamine-related antiapoptotic signaling during IRI, we subjected WT mice to IRI or sham surgery and administered glutamine or saline and the Tgm2 inhibitor ERW1041E or 10% DMSO (vehicle control), respectively. Combined administration of glutamine and ERW1041E almost completely reversed the glutamine-induced decreased plasma creatinine levels and reduced neutrophil infiltration induced by glutamine treatment (Figure 4, D and E). To additionally show the functional relevance of glutamine-induced Tgm2 upregulation following renal IRI, we quantified Tgm2 activity in the kidneys. Tgm2 activity was significantly increased after glutamine treatment, while Tgm2 inhibitor treatment completely abolished this effect (Figure 4F).

To verify the effect of glutamine on Tgm2, we generated proximal TEC-specific knockout mice (Tgm2^fl/fl^ sGLT2^cre+^) and subjected them to IRI surgery following glutamine or saline treatment. We analyzed plasma and urine creatinine levels as well as neutrophil recruitment into the kidneys and observed that glutamine had no significant protective effect on kidney damage, inflammation, and the renal tubular injury score in Tgm2–conditional knockout mice anymore (Figure 4, G–J).

### Glutamine supports mitochondrial respiration during oxidative stress.

**Figure 5:**
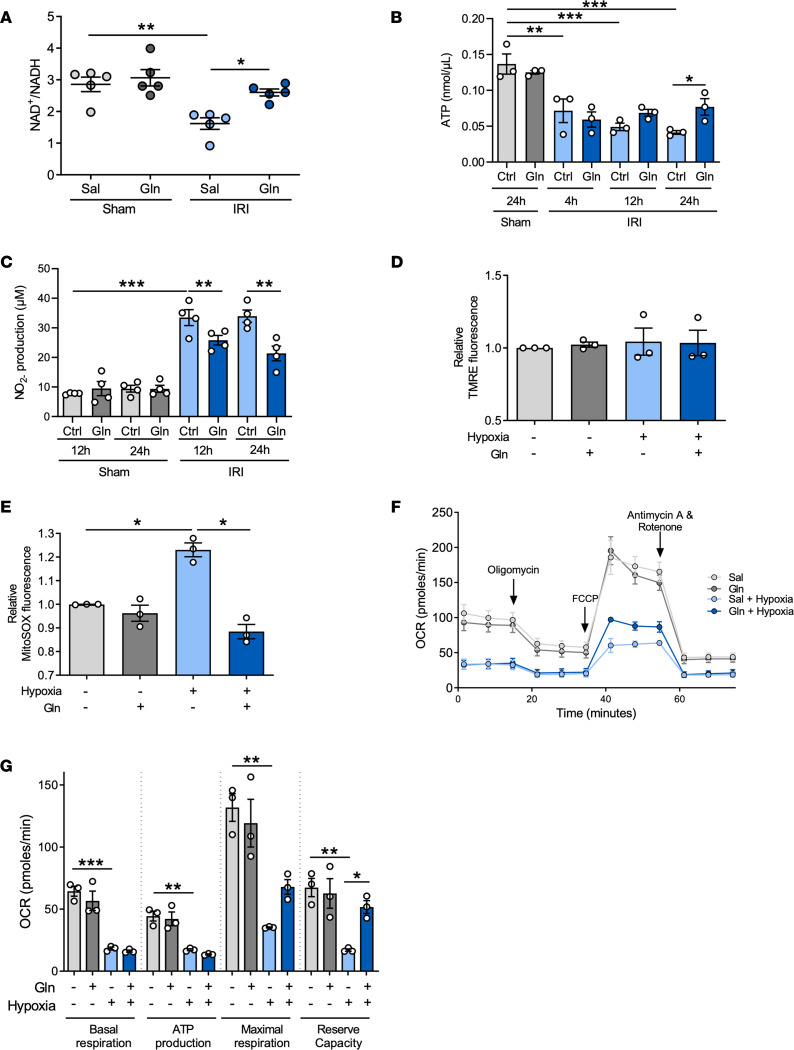
Glutamine supports mitochondrial respiration during oxidative stress. WT mice were subjected to sham or IRI surgery and received glutamine or saline. NAD^+^/NADH ratio (**A**, *n* = 5), ATP concentration (**B**, *n* = 3), and NO_2_^–^ concentration (**C**, *n* = 4) were detected in kidney suspensions 24 hours after IRI by colorimetric assays. TECs were treated with glutamine or saline and subsequently subjected to hypoxic (1% O_2_) conditions for 24 hours. The mitochondrial membrane potential was assessed by TMRE fluorescence detection (**D**, *n* = 3). Mitochondrial ROS production was detected to assess oxidative stress (**E**, *n* = 3). Mitochondrial respiration was assessed using the Seahorse XF24 Flux Analyzer. The oxygen consumption rate (OCR) to analyze mitochondrial respiration was measured using the Mito Stress Kit (**F** and **G**; *n* = 3). Mean ± SEM; 1-way ANOVA. *P < 0.05; **P < 0.005; ***P < 0.001. FCCP, carbonyl cyanide-*p*-trifluoromethoxyphenylhydrazone.

To analyze the effect of glutamine treatment on the cellular reduction-oxidation equilibrium, glycolysis, and energy metabolism, we quantified the NAD^+^/NADH ratio, ATP concentration, as well as NO_2_^–^ levels in kidney tissue after IRI induction (Figure 5, A–C). We found kidney tissue NAD^+^/NADH ratio and ATP concentration to be increased in glutamine-treated IRI mice (Figure 5, A and B) while NO_2_^–^ levels were decreased upon glutamine treatment (Figure 5C). As the maintenance of an optimal NAD^+^/NADH ratio is essential for mitochondrial function, we further focused on the effect of glutamine on mitochondrial functionality and respiration in TECs. Therefore, we analyzed the mitochondrial membrane potential and the mitochondrial ROS (mROS) production upon hypoxia with or without glutamine treatment for 24 hours. The mitochondrial membrane potential was not affected by hypoxia or glutamine/saline treatment, while the mROS production was enhanced under hypoxic conditions (Figure 5, D and E). The observed mROS increase was significantly diminished by glutamine treatment (Figure 5E). To assess mitochondrial respiration upon hypoxic stress, we analyzed the oxygen consumption rate of TECs using the Seahorse Analyzer and found hypoxia to decrease basal and maximal respiration (Figure 5F). Glutamine treatment led to a partial increase of maximal respiration and a consequent elevated reserve capacity, thereby positively regulating oxidative phosphorylation (Figure 5G). In contrast to hypoxia, TNF-α did not affect mitochondrial functionality (Supplemental Figure 5, A–D).

### Glutamine affects the expression of apoptosis- and oxidative stress–related proteins in renal TECs on the transcriptional and proteomic level.

**Figure 6:**
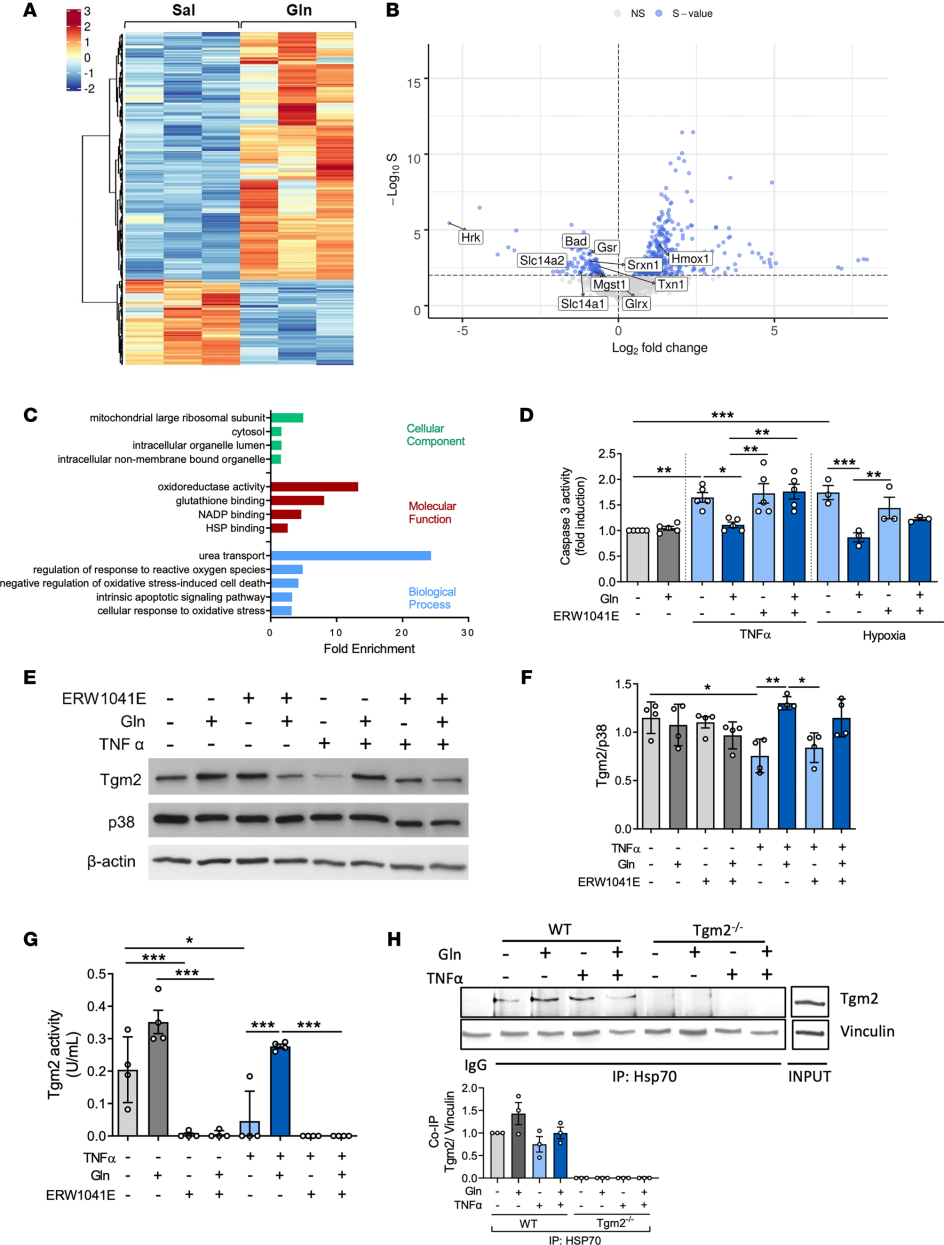
Glutamine affects the expression of apoptosis- and oxidative stress–related proteins in renal TECs on the transcriptional and proteomic level. TECs were identified in kidney homogenates by FACS based on the cell-specific marker prominin-1. RNA was isolated and sequenced. Hierarchical clustering heatmaps of RNA-Seq indicate differentially expressed genes (rows) between TECs of glutamine-treated mice after IRI and glutamine treatment compared with vehicle control (**A**, *n* = 3). Blue color indicates downregulation and red color indicates upregulation. Volcano plots generated to compare glutamine versus saline treatment after IRI induction identify 481 differently expressed genes in renal TECs (**B**). GO pathway enrichment analyses specify the fold enrichment of the transcriptome sequencing relative to the *Mus* *musculus* genome in TECs (**C**). GO terms representing molecular function are presented in red, cellular component in green, and biological processes in blue (Fisher’s exact test, *P* value < 0.05). TECs were isolated from murine kidneys and exposed to TNF-α for 18 hours or hypoxia for 24 hours. Additionally, TECs were treated with glutamine or saline as well as Tgm2 inhibitor ERW1041E or vehicle control, respectively. Caspase-3 activity was detected after TNF-α (*n* = 5) or hypoxia stimulation (*n* = 3, **D**), and Tgm2 levels were determined by Western blotting (**E** and **F**). Tgm2 activity in kidneys was assessed (**G**, *n* = 4). Immuno- and coimmunoprecipitated proteins were separated by SDS-PAGE and blotted using the indicated antibodies. Whole-cell lysate (INPUT) was used as protein control (**H**, *n* = 3). Mean ± SEM; 1-way ANOVA *P < 0.05; **P < 0.005; ***P < 0.001.

To analyze glutamine-related effects on renal TEC, we sorted the cells from kidney tissue by FACS 24 hours after IRI induction, isolated RNA, and performed transcriptome analysis by RNA-Seq. In renal TECs unbiased hierarchical clustering clearly separated the glutamine-treated from the saline-treated mice (Figure 6A). Functional annotation analyses identified 481 differently expressed genes upon renal IRI and glutamine treatment (Figure 6, A and B, and Supplemental Table 2). GO analyses revealed that the differentially detected genes are members of pathways related to apoptosis-related (*Hrk*, *Srxn1*, and *Bad*) and oxidative stress–related (*Trx1*, *Grx1*, *Gsr*, *Mgst1*, and *Hmox1*; Figure 6C) signaling. Caspase-3 activity assessment following TNF-α stimulation and hypoxia induction revealed glutamine to reduce apoptosis in TECs, thereby verifying the RNA-Seq results (Figure 6D).

To verify the glutamine-related effects on Tgm2 reflected by the proteomic analysis, we performed in vitro experiments using isolated murine TECs, treated them with glutamine in vitro, and analyzed Tgm2 levels as well as Tgm2 activity upon TNF-α stimulation (Figure 6, E–G). We found both Tgm2 levels and activity to decrease upon TNF-α stimulation, which was reversed by additional glutamine treatment. Moreover, Tgm2 inhibition completely abolished Tgm2 activity and reduced Tgm2 levels in renal TECs; further addition of glutamine had no effect in the presence of the Tgm2 inhibitor. We performed similar experiments using immortalized human kidney epithelial-1 (IHKE-1) cells in vitro. Quantification of TIMP-2 and IGFBP7 levels revealed a significant increase of both markers upon TNF-α stimulation, and this effect was ameliorated by glutamine treatment (Supplemental Figure 6A). According to the effects we observed in our murine in vivo experiments, immunoblotting of IHKE-1 cells also demonstrated upregulation of HSP70 and p-Bad and downregulation of p14-3-3ζ upon glutamine treatment in vitro (Supplemental Figure 6, B–E). These results were confirmed by immunoblotting of isolated murine TECs, where siRNA-mediated knockdown of Tgm2 reduced or even abolished the effects of glutamine on downstream signaling (Supplemental Figure 7). Thus, in total kidney tissues from IRI mice or in isolated TECs subjected to TNF-α–induced stress, glutamine treatment induced expression of Tgm2 and HSP70 to protect cells from cellular injury and apoptosis. To investigate if Tgm2 modulates HSP70 activation by a direct interaction, we performed co-immunoprecipitation with WT and Tgm2^–/–^ TECs. The analysis revealed Tgm2 interacts with HSP70 and that glutamine enhances the interaction in WT TECs (Figure 6H).

To exclude possible effects of glutamine on the investigated cell-intrinsic functions, we analyzed cell viability of TECs after incubation with glutamine and the Tgm2 inhibitor ERW041E and could not detect significant effects on survival (Supplemental Figure 8, A–C). To demonstrate TNF-α and hypoxia inducing apoptosis in TECs, we analyzed cell viability upon each treatment and found the percentage of apoptotic and necrotic cells to be increased after both stimulations (Supplemental Figure 8, D and E).

## Discussion

This study demonstrates that the administration of glutamine protects the kidney from IRI-induced AKI in vivo. Consistently, we found that the extent of renal tubular cell damage and apoptosis as well as neutrophil recruitment are significantly reduced after glutamine administration in vitro. Mechanistically, glutamine administration modulated proapoptotic c-Jun signaling in renal TECs, and the renal protective effect of glutamine was conferred by Tgm2. The role of glutamine in AKI has been studied in experimental models in the past (15). However, these older studies are rather descriptive while in this study we reveal the underlying molecular pathway by which glutamine exerts its protective effect from AKI on a comprehensive scale. We show that glutamine preserved Tgm2 levels and reduced Ask1 activation in renal TECs. Tgm2 directly interacted with HSP70, correlating with increased HSP70 levels. Both elevated HSP70 expression and reduced Ask1 phosphorylation decreased activation of the intrinsic apoptotic JNK pathway during AKI.

Our experimental findings are in accordance with previous studies investigating the impact of glutamine on inflammation and tissue damage. Several studies focusing on the beneficial effects of glutamine on immune cells investigating bacterial killing (26) or airway neutrophilia (27) further support the observed antiinflammatory effect of glutamine. Moreover, glutamine has been shown to attenuate tubular cell apoptosis (15) and to reduce renal dysfunction (16), supporting the hypothesis that glutamine has both antiinflammatory and cytoprotective properties. Specifically, our study reveals that glutamine affects both signaling pathways in renal TECs. Our results are also consistent with studies of glutamine in nephrotoxicity models in mice in that activation of HSP70 mitigates inflammation and renal neutrophil infiltration (16).

In a broad analytic approach, we analyzed the effects of glutamine on a whole-proteome scale. Our data show that glutamine treatment results in a significant upregulation of proteins that are associated with oxidative phosphorylation in mitochondria, in particular vital subunits of the NADH:ubiquinone oxidoreductase (complex I). Additionally, proteins that are part of apoptosis and oxidative stress pathways, i.e., the glutathione system and related NAD^+^ binding processes, were upregulated upon glutamine administration. Oxidative stress develops as a result of imbalance between ROS production and antioxidant defense (e.g., ROS scavenging) (28). Impaired NAD^+^ synthesis has been shown to augment the pathogenesis of AKI (29). Interestingly, in a phase I trial, enhanced NAD^+^ biosynthesis decreased the postoperative AKI incidence in cardiac surgery patients (29). Glutamine is known to be involved in the regulation of oxidative stress as it is a precursor for many antioxidative molecules, such as glutathione and NAD^+^ (10, 30–32). Hence, glutamine administration might support glutathione synthesis on the one hand and increase the NAD^+^/NADH ratio and subsequent ROS scavenging on the other hand. This is in line with previous reports describing similar effects of glutathione in an in vivo model of hepatic injury induced by paraquat (33) and of glutamine on the NAD^+^/NADH ratio in clinical trials of sickle cell disease (34). Additionally, glutamine has been shown to cause enhanced NAD^+^ accumulation (35) and NAD^+^, in turn, to exert cytoprotective effects in renal cells (36, 37).

On a cellular level, the pathogenesis of AKI involves apoptosis of renal tissue. Renal TECs are particularly vulnerable for apoptosis induction, and AKI is linked to the endogenous activation of proapoptotic signaling pathways in these cells, e.g., the Bad/Bax proapoptotic signaling pathway (38). Beyond the amelioration of oxidative stress, we also observed by using biochemical assays that glutamine administration appears to modulate apoptosis signaling pathways by downregulation of several apoptosis-related proteins, e.g., Pdcd-6 and Pdcd-10. Our results reveal glutamine to enhance intracellular Tgm2 and HSP70 expression levels, while Bad phosphorylation (Ser136) appeared increased and 14‑3‑3 phosphorylation (Thr-232) were decreased following glutamine treatment. Bad and 14-3-3 are essential components of the mitochondrion-mediated intrinsic apoptotic pathway (39, 40) and suppressors of BCL-XL and its antiapoptotic function (41). Thus, increased phosphorylation of Bad and decreased phosphorylation of 14-3-3ζ upon glutamine administration in our renal IRI model might result in reduced proapoptotic signaling in renal structures. In harmony with our current findings, HSP70 has been shown to be upregulated after glutamine administration in an in vivo model of ischemic AKI induced by glycerol injection (15). Importantly, HSP70 has been reported to protect cells from apoptosis by inactivating JNK, which prevents Bad and Bax activation in intrinsic apoptotic signaling. It has also been demonstrated that glutamine induces the expression of the antiapoptotic protein Bcl-2 in HK-2 cells, further supporting our data (14).

Our proteomic and MALDI-IMS analysis revealed that glutamine treatment significantly upregulates Tgm2, a multifunctional enzyme with Ca^2+^-dependent protein cross-linking activity and GTP-dependent G protein functions. Tgm2 has been previously reported to directly regulate heat shock responses by interacting with HSP70, which is in accordance with our own findings (42, 43). Furthermore, the observed upregulation of HSP70 by enhanced Tgm2 levels may additionally contribute to the antiapoptotic effect of glutamine on kidney cells during IRI, similar to the role of HSPs in renal protection during obstructive nephropathy (44). Although Tgm2 is known to also possess opposing roles in apoptotic processes in other scenarios (45), our data suggest Tgm2 represents an important contributor of the cytoprotective effect of glutamine supplementation. However, Tgm2 is an important enzyme with multiple effects that may be required for cell function and integrity, particularly under conditions of stress or injury. Although the protective effects of glutamine appear to require Tgm2, renal IRI itself also modulates Tgm2 levels, suggesting the effects may be indirectly caused by the metabolic benefits of glutamine. Tgm2 also catalyzes the formation of covalent bonds between peptide-bound glutamine and various primary amines, such as gamma-amino group of peptide-bound lysine, or mono- and polyamines. Thereby it may mediate the cross-linking of several proteins, and glutamine is highly necessary for this catalyzed reaction. Thus, we assume that the observed effects of glutamine on Tgm2 levels might be an additive effect of direct interaction and indirect metabolic benefits caused by glutamine.

In addition, we found Ask1 activation to be reduced upon glutamine treatment. Ask1 acts as an upstream regulator for the activation of p38 MAPK and JNK in kidney disease. Ask1 was already suggested to constitute a new therapeutic target as Ask1^–/–^ mice exhibited decreased kidney injury after IRI induction (46). Thus, active Ask1 facilitates downstream JNK signaling, resulting in progression of injury. By demonstrating Ask1 phosphorylation to be decreased after glutamine treatment, we reveal another beneficial effect of glutamine leading to diminished apoptosis.

On a transcriptional level in renal TECs, we found proapoptotic genes (*Hrk*, *Bad*) to be downregulated and cytoprotective genes (*Srxn1*, *Hmox1*) to be upregulated by glutamine treatment. Both sulfiredoxin-1 (product of *Srxn1*) and heme oxygenase-1 (product of *Hmox1*) are known for their renoprotective effects in experimental models of AKI (47, 48). Moreover, we found antioxidative genes downregulated in renal TECs treated with glutamine, showing a reduced state of oxidative stress in these cells. In addition, we further substantiate glutamine’s protective effects on oxidative stress management as we demonstrate that glutamine decreased mROS release, which are mainly produced at the electron transport chain during the oxidative phosphorylation process (49, 50). This finding is in accordance with a previous study of glutamine alleviating kidney lipid peroxides’ production in a polymicrobial sepsis mouse model induced by cecal ligation and puncture (51). Beyond the reduced mROS production, we provide evidence that glutamine ameliorates maximal mitochondrial respiration and a consequent elevated reserve capacity of TECs upon hypoxia. This value specifies the capability of the cell to respond to an energetic demand and serves as an indicator of cell fitness or flexibility. This is in line with our proteomic analysis that revealed mitochondrial oxidative phosphorylation to be affected by glutamine and further stresses the protective effects of glutamine on renal tissue and functionality upon ischemic insults during cardiac surgeries and a subsequent AKI progression.

The IRI model used in this study is highly comparable to patients who undergo cardiac surgery with the use of cardiopulmonary bypass. These patients are prone to developing AKI as the implementation of cardiopulmonary bypass itself decreases the renal perfusion and exposes the renal parenchyma to reduced oxygen tension, which contributes to renal IRI (52). Thus, our results here remarkably suggest glutamine as a therapeutic drug that might be used in patients 4 hours after cardiac surgery, the earliest time point AKI development may be clinically predicted by renal AKI biomarker assessment such as [TIMP-2]*[IGFBP7].

In conclusion, we demonstrate that glutamine attenuates AKI and tissue damage following renal IRI by directly supporting antiapoptotic signaling pathways increasing renal resistance against tubular cell injury on the one hand and reducing neutrophil infiltration and activation on the other hand. Thus, we here provide important evidence for the therapeutic potential of this already clinically available drug in surgical patients at risk for the development of AKI.

## Methods

### Mice.

We used 8- to 12-week-old C57BL/6J (obtained from Jackson Laboratories), Tgm2^–/–^ (53), and Tgm2^fl/fl^ sGLT2^cre+^ male mice, which were kept under specific pathogen–free conditions. All mice had ad libitum access to food and water and were maintained with a 12-hour light/12-hour dark cycle. Animals were randomly assigned to *n* = 3–8 mice/group per experiment, except where indicated otherwise, and data represent at least 3 independent experiments throughout.

### Renal IRI.

The IRI model has been described previously (54). In brief, mice were anesthetized by intraperitoneal administration of ketamine (100 μg/g bw) and xylazine (10 μg/g bw) and were placed on a heating pad to maintain body temperature. In animals undergoing IRI, both renal pedicles were clamped off for 35 minutes with hemostatic micro clips. After clamp removal, kidneys were checked for a change in color to ensure reperfusion. In sham controls the surgical procedure was identical except that no clamps were applied. Incisions were closed in 2 layers and the mice were kept in metabolic cages to quantify urinary excretion. Glutamine [N(2)-L-Alanyl-L-Glutamin; Dipeptiven; Fresenius Kabi Deutschland GmbH, 200 mg/mL concentrate for reconstitution] was reconstituted in 0.9% physiologic saline solution and administered intravenously (0.5 g/kg bw in 200 μL). 0.5 g/kg bw represents the specified and suggested maximum dose per day. As a control 0.9% physiologic saline solution was applied. Mice were euthanized after 4 hours, 12 hours, or 24 hours, respectively; blood samples were taken by heart puncture; and kidneys were harvested.

### Flow cytometry and FACS strategies.

Flow cytometry was performed to assess neutrophil recruitment to the kidney (FACSCanto 2; BD Biosciences) based upon the expression of CD45 (clone 30-F11, BioLegend, 103132), GR-1 (clone RB6-8C5, purified from hybridoma supernatant), and Ly6B.2 (clone 7/4, Bio-Rad, MCA771G). Isotype controls were employed to account for nonspecific antibody binding. Anti-mouse GR-1 antibody was purified from supernatant of the GR-1 hybridoma (ATCC) and fluorescently labeled with an F(ab)-based staining kit following manufacturer’s instructions (Alexa Fluor 633, Invitrogen).

For bulk RNA-Seq analysis, renal TECs were FACS-sorted from kidney tissue of mice treated with glutamine or saline after IRI induction, based upon the expression of CD45^–^Prominin-1^+^ (clone: MB9-3GB, Miltenyi Biotec, 130-102.197).

### Protein extraction and Western blot.

Kidneys were homogenized in lysis buffer (10 mM sodium orthovanadate, 200 μM PMSF, 20 μM leupeptin, and 0.15 μM pepstatin). IHKE-1 cells (gift from G Ciarimboli, Internal Medicine D, University Hospital Münster, Münster, Germany) (55) and isolated proximal TECs were lysed in Homburg lysis buffer (10 mM Tris/HCl pH 7.5, 10 mM NaCl, 0.1 mM EDTA, 0.5% Triton X-100, 20 mM NaN_3_, 1 mM PMSF, 1 mM sodium orthovanadate). Protein concentrations were determined using a bicinchoninic acid kit (Pierce BCA Protein Assay Kit, Thermo Fisher Scientific). Equal amounts of kidney and IHKE-1 cell lysates (30 μg protein) as well as TEC lysates (5 μg protein) were subjected to Western blotting according to standard procedures (Bio-Rad, Mini-PROTEAN Tetra Cell System). Membranes were blocked in TBS-Tween (TBST) + 5% BSA for 1 hour at room temperature before being probed with primary antibodies at 4°C overnight (anti–Tgm2 [D11A6], Cell Signaling Technology [CST], 3557; anti-HSP70, CST, 4872; anti–p38 MAPK, CST, 9212; anti 14-3-3ζ, AAT Bioquest, 8C12007; anti–phospho-14-3-3ζ Thr232, Invitrogen, PA5-38398, anti–Bad D24A9; CST, 9239; anti–phospho-Bad Ser136 D25H8, CST, 4366; anti–β-actin, CST, 4967; anti–caspase-3, CST 9662; anti–phospho-c-Jun [Ser73] 9164, anti-ASK1 [EP553Y], Abcam ab45178; anti–phospho-Ask1 [Thr845], Thermo Fisher Scientific BS-3031R; anti-Vinculin, CST 4650; anti–α-Tubulin, MilliporeSigma, T6074; anti-Akt, CST 4691; anti–phospho-Akt [Ser473], CST 4058; PI3K, CST 4255); washed 3 times in TBST for 10 minutes; and incubated with HRP-linked secondary antibody (anti-rabbit antibody, CST, 7074) for 1 hours at room temperature. Chemiluminescence signals were recorded on Amersham Hyperfilm ECL films (GE Healthcare, now Cytiva). Densitometric quantification was performed using ImageJ software (NIH).

### Immunoprecipitation.

Isolated TECs were lysed in a buffer containing 1× TBS, 0.01% NP-40, 200 μM PMSF, cOmplete inhibitor (Roche, 11697498001), 50 mM sodium fluoride, 2 mM sodium orthovanadate, and 30 mM sodium pyrophosphate. Immunoprecipitation was performed with 0.14 μg HSP70 antibody (CST, 4872) overnight at 4°C rotating. Protein G beads (MilliporeSigma, P3296) were added for 3 hours at 4°C rotating before 3× sample buffer was added and samples were boiled at 95°C for 10 minutes. Supernatants were analyzed by Western blot.

### Creatinine, urea nitrogen, and Tgm2 activity.

Plasma and urine creatinine as well as urea nitrogen levels were determined by using a creatinine assay (Diazyme) and a urea nitrogen (BUN) Colorimetric Detection Kit (Invitrogen, EIABUN) according to the manufacturer’s protocols. Tgm2 activity in kidneys was measured using a colorimetric assay (Transglutaminase 2 Assay Kit, Novus Biologicals, NBP1-37008) following the manufacturer’s directions.

### In vivo and in vitro inhibition of transglutaminase activity.

WT mice were subjected to IRI or sham surgery. In addition to glutamine or saline exposure, mice were treated with the Tgm2 inhibitor ERW1041E (25 mg/g) or control (DMSO 10%), respectively, by an intravenous injection. At 12 hours after IRI or sham induction, mice were euthanized, blood samples were taken by heart puncture, and kidneys were harvested. One kidney was used to assess neutrophil recruitment via flow cytometry while the other one was homogenized by a tissue douncer in lysis buffer for Tgm2 activity detection.

Renal proximal TECs were isolated as previously described (56). Briefly, kidneys were harvested and subjected to mechanical and enzymatic dissociation by using collagenase type II enzyme (Worthington Biochemical) for digestion. TECs were then isolated by magnetic separation based on magnetic microbeads conjugated with an anti–prominin-1 antibody that specifically targets the proximal tubules. TECs were cultivated on cell culture dishes and maintained in renal epithelial cell growth medium supplemented with growth factors (Lonza, CC-3191, CC-4127) at 37°C in 5% CO_2_. The purification rate was 91.7% (Supplemental Figure 3A). TECs were preincubated with 0.0083 g/mL glutamine or saline, respectively for 2 hours. Tgm2 inhibitor ERW1041E (25 μM) or DMSO was added for a 1-hour incubation. Cells were washed, lysed, and used for Western blot analysis as well as Tgm2 activity measurement.

### siRNA-mediated KD induction.

The ON-TARGETplus nontargeting siRNA control pool and Tgm2 and Ask1 ON-TARGETplus siRNAs were purchased from Horizon by Dharmacon. On 24-well plates seeded isolated murine TECs were 60% to 80% confluent on the day of transfection. The siRNAs were transfected using Lipofectamine RNAiMAX Reagent (Thermo Fisher Scientific, 13778030) according to manufacturer protocol. Briefly, the siRNA duplex solution (10 nM) was added to the diluted transfection reagent in a 1:1 ratio and incubated for 10 minutes at room temperature. Subsequently, the 50 μL of the mixture was added to each well containing cells and medium. After 30 hours of incubation at 37°C and 5% CO_2_, cells were subjected to glutamine or saline treatment and TNF-α stimulation for 2 hours. Protein expression levels and phosphorylation were analyzed by Western blot.

### ELISA.

The plasma and urine levels of mediators and biomarkers (IL-10, TNF-α, IL-6, CXCL1, CXCL2, HMGB1, IGFBP7, TIMP-2, NGAL, L-FABP, and PCX) were analyzed using commercial available ELISA kits (Mouse IL-10 Quantikine ELISA Kit, R&D Systems, M1000B; Mouse TNFα Quantikine ELISA Kit, R&D Systems, MTA00B; Mouse IL-6 Quantikine ELISA Kit, R&D Systems, M6000B; Mouse CXCL1/KC Quantikine ELISA Kit, R&D Systems, MKC00B; Mouse CXCL2/MIP2 Quantikine ELISA Kit, R&D Systems, MM200; HMGB1 ELISA, IBL-International, ST51011; IGFBP7 ELISA Kit, Antikörper-Online, ABIN816449, Mouse TIMP-2 DuoSet ELISA, R&D Systems, DY6304-05, Mouse Lipocalin-2/NGAL Quantikine ELISA Kit, R&D Systems, MLCN20, Mouse/Rat FABP1/L-FABP Quantikine ELISA Kit, R&D Systems, RFBP10: PODXL ELISA Kit Mouse, Aviva Systems Biology, OKEH03213). The ELISA plates were quantitatively analyzed on a BioTek Synergy 2 plate reader.

### IHC.

Isolated kidneys were fixed in 4% formaldehyde, embedded in paraffin, and sectioned at 2 μm for H&E, TUNEL, and IHC staining. TUNEL staining was performed with the DeadEnd Colorimetric TUNEL system (G7130, Promega) strictly according to the manufacturer’s instructions. Apoptotic cells in cortex and medulla tissue were quantified in a blinded fashion by reviewing 10 visual fields for each slide to show differences in apoptosis by illustrating the results as apoptotic cells/field. Percentage of apoptotic cells were calculated according to positive and negative control cell counts.

### Renal tubular injury score.

Tubular injury was scored by estimating the percentage of tubules in the cortex or the outer medulla that showed epithelial necrosis or had luminal necrotic debris and tubular dilatation as follows: 0 = none; 1 = <5%; 2 = 5%–30%; 3 = 31%–75%; and 4 = >75%. For each slide, at least 10 fields were reviewed at an original magnification of ×200 (DM5500B; Leica Biosystems). Histopathological scoring of H&E staining was performed by a renal pathologist blinded to the conditions.

### Measurement of mitochondrial respiration.

For mitochondrial respiration assessment in TECs oxygen consumption rate, measurements were performed by employing XF_p_ Seahorse extracellular flux analyzer (Agilent). A monolayer of renal tubular epithelial cells (0.5 × 10^5^ TECs per well) was seeded on a Seahorse miniplate and grown for 24 hours at 37°C and 5% CO_2_ until confluent. To induce cell stress, glutamine- or saline-treated TECs were incubated for 18 hours at 37°C either under hypoxic conditions (1% O_2_) or TNF-α stimulation. Oxygen consumption was blocked by oligomycin (1.5 μM), an ATP synthase inhibitor; the ionophore FCCP assayed maximal respiratory capacity of mitochondria, whereas rotenone plus antimycin A (3 μM), a mitochondrial inhibitor, was used to block mitochondrial respiration. Three independent experiments were performed, and 3 technical replicates per experiment were measured to calculate the mean ± SEM for each time point.

### Measurement of mitochondrial functionality and reduction-oxidation status.

Confluent TECs seeded on 96-well plates were treated with glutamine and incubated for 24 hours at 37°C either under hypoxic conditions (1% O_2_) or while exposed to TNF-α. Functionality assays were performed according to the manufacturer’s instructions. To examine changes in the mitochondrial membrane potential, the TMRE-Mitochondrial Membrane Potential Assay Kit (Abcam, ab113852) was used, and TMRE-positive cells were analyzed by a microplate reader (excitation/emission [Ex/Em] 548/575 nm). Mitochondrial superoxide production of glutamine- or saline-treated TECs after stimulation was assessed by using the MitoSOX Red reagent (Invitrogen, M36008), which oxidizes in the presence of superoxide and generates a fluorescence signal that was analyzed by a microplate reader (Ex/Em 510/580 nm). To analyze NO_2_^–^ formation, the colorimetric Griess Reagent System was implemented (Promega, G2930), which is based on the chemical reaction employing sulfanilamide and N-(1-Naphthyl)ethylenediamine dihydrochloride under acidic conditions that was measured in the microplate reader at 520 nm. Confluent TECs seeded on 6-well plates were treated as previously described. Kidneys isolated from mice that underwent sham or IRI surgery treated with glutamine or saline were homogenized in PBS by a tissue douncer and centrifuged at 10,000*g* for 5 minutes at 4°C. The supernatant was used for Griess assay. NAD/NADH ratios and ATP concentrations of isolated kidneys were measured using colorimetric assays (NAD/NADH Assay Kit, Abcam, ab65348, ATP Assay Kit, Abcam, ab83355).

### Measurement of caspase-3 activity.

The activity of caspase-3 was assessed using a Caspase-3 Colorimetric Assay kit (Caspase-3 Assay Kit, Abcam, ab39401), which is based on the spectrophotometric detection of the color reporter molecule p-nitroaniline (pNA) following cleavage from the labeled substrate DEVD-pNA (caspase-3) as an index. TECs were treated with glutamine or saline and stimulated with either TNF-α or hypoxia induction for 18 hours before caspase-3 activity was detected. Kidneys isolated from mice that underwent sham or IRI surgery treated with glutamine or saline were homogenized in caspase-3 lysis buffer using a tissue douncer and centrifuged at 10,000*g* for 5 minutes at 4°C. Caspase-3 activity was detected in the supernatant.

### RNA-Seq analysis.

RNA was extracted from the sorted cells using an RNeasy Mini Kit (QIAGEN). Isolated RNA samples were converted into cDNA by reverse transcription. PCR products were purified; libraries were constructed and sequenced to 50 bp. The differential expression analysis was carried out by DESeq2 (57) on the gene-level counts.

Additional experimental procedures and methods are described in the Supplemental Methods.

### Mass spectrometry.

Kidneys were removed 24 hours after IRI induction and homogenized in lysis buffer (2% Triton X-100, MilliporeSigma, X100; 0.1× HALT Protease Inhibitor, Thermo Fisher Scientific, 78429; 10 IE/mL heparin, Ratiopharm; 0.1 M 6-aminocaproic acid, MilliporeSigma, A2504; 5 mM EDTA, MilliporeSigma, EDS; pH 7.4). Kidney homogenates were resuspended in urea buffer (6 M urea, 2 M thiourea in 10 mM Tris pH 8.0) and equal amounts were purified using SDS-PAGE (Invitrogen). Coomassie-stained gel pieces were excised and in-gel–digested using trypsin as described previously (58). Extracted peptides were desalted using C18 StageTips (59) and subjected to liquid chromatography-tandem mass spectrometry (LC-MS/MS) analysis. LC-MS/MS analyses were performed on an Easy nano-LC (Thermo Fisher Scientific) coupled to an LTQ Orbitrap Elite (Thermo Fisher Scientific) as described elsewhere (60). Label-free quantification (LFQ) protein intensities were used for relative protein quantification analysis. Statistical analysis was done with Perseus 1.5.0.15 using *P* value of 0.05 and taking also those proteins in the calculation that did not have LFQ intensity in all 4 samples, but in 3 or 2. This gave us approximately 1,500 proteins to calculate the statistics. The majority of the significantly changing proteins are of a low intensity and are on the border of not being significant. Only several proteins with high intensity also express a high fold change.

### MALDI-IMS.

Kidney organ sections were dewaxed, tryptically digested, and coated with matrix (α-cyano-4-hydroxycinnamic acid, Bruker Daltonics) for the MALDI TOF/TOF measurement with a sprayer for MALDI imaging (HTX TM-Sprayer: TMSP-M3, HTX Technologies). MALDI-IMS was performed using the Rapiflex (Bruker Daltonics). Experimental mass spectrometric data were compared with calculated peptide masses for each entry in the sequence database.

Additional experimental procedures and data processing are described in the Supplemental Methods.

### Renal ultrasound.

Ultrasound measurements were performed on a Vevo 2100 ultrasound system (FUJIFILM VisualSonics) at 40 MHz using a high-resolution transducer (MS550D) for vascular imaging in mice. After real-time acquisition of the pulse wave velocity, the peak systolic velocity and the lowest diastolic velocity of the blood flow of the *A. renalis* was calculated at baseline conditions as well as 4 hours and 24 hours after IRI induction. The following formula was used to calculate the renal arterial resistivity index: RI = (peak systolic velocity – lowest diastolic velocity)/peak systolic velocity.

### Study limitation.

This study has the limitation that sex as a biological variable was not considered, as we only used male mice for their enhanced injury and their stable creatinine levels in the used IRI model.

### Data availability.

The transcriptomic data in this publication have been deposited in the National Center for Biotechnology Information Gene Expression Omnibus and are accessible through accession GSE188628. The mass spectrometry proteomics data have been deposited to the ProteomeXchange Consortium via the PRIDE partner repository with the data set identifier PXD029723. All original data are accessible through the corresponding author upon reasonable request.

### Statistics.

All statistical calculations were performed using the GraphPad Prism software (version 6). Data distribution was assessed using Kolmogorov-Smirnov test or Shapiro-Wilk test; differences between groups were analyzed using the Wilcoxon test or *t* test as appropriate. More than 2 groups were compared using 1-way ANOVA followed by Bonferroni correction. Time course curves were compared using 2-way ANOVA followed by Bonferroni correction. All data are represented in means ± SEM. A *P* < 0.05 was considered as statistically significant.

### Study approval.

All animal experiments were performed in accordance with institutional and federal guidelines and approved by the institutional review board (Landesamt für Natur-, Umwelt- und Verbraucherschutz Nordrhein-Westfalen, Recklinghausen, Germany).

## Author contributions

KT, LZ, NL, MK, CL, KH, SM, AM, AV, RH, JH, and MM performed experiments and analyzed data. TS, TT, and VJ analyzed data. KT, DV, J Roth, MAS, J Rossaint, and AZ interpreted data. LZ, JAK, and CAL contributed to writing the manuscript. MAS, J Rossaint, and AZ designed the study. MW gave input on previous experimental and clinical findings with glutamine. J Rossaint and AZ supervised the study.

## Supplementary Material

Supplemental data

## Version 1 09/15/2022

In-Press Preview

## Version 2 11/08/2022

Electronic publication
