# Supplementary material for: Glutamine prevents acute kidney injury by modulating oxidative stress and apoptosis in tubular epithelial cells
Source: JCI Insight. 2022 Nov 8;7(21):e163161. doi: 10.1172/jci.insight.163161 (PMC9675453; doi:10.1172/jci.insight.163161)
Supplement: Supplemental data [file jciinsight-7-163161-s082.pdf]

Supplementary Figures

Figure S1

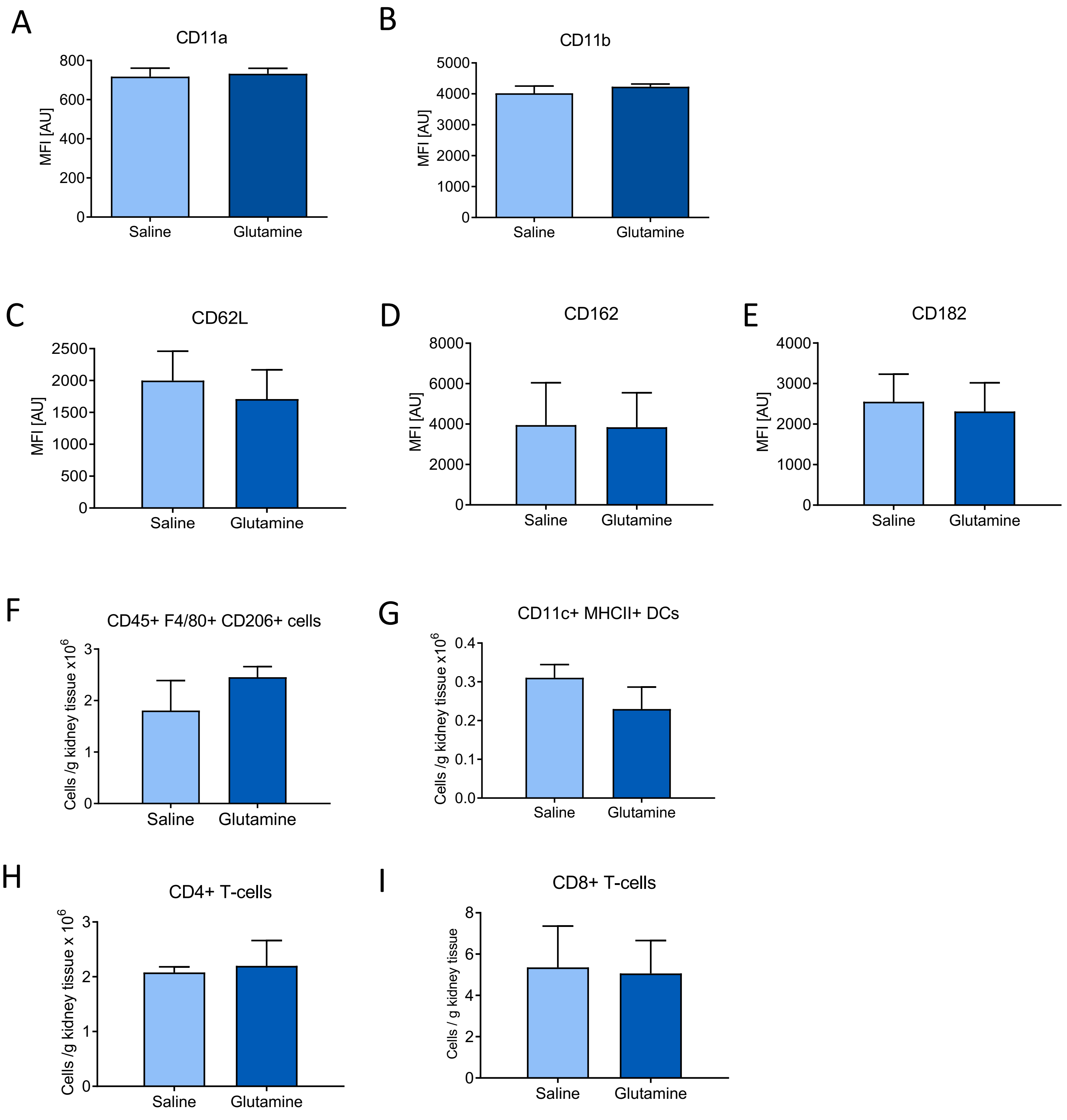

Figure S1: Characterization of infiltrated immune cells after IRI. WT mice were subjected to IRI surgery and received glutamine or saline 4h after reperfusion. Kidneys were isolated 24h after IRI induction, homogenized and prepared for expression molecule analysis via flow cytometry. Mean Fluorescent Intensities (MFIs) were analyzed for the expression markers CD11a (A), CD11b (B), CD62L (C), CD162 (D) and CD182 (E). Cell counts per g kidney tissue were assessed for CD45+ F4/80+ CD206+ Macrophages (F), CD11c+ MHCII+ dendritic cells (DC; G) as well as CD4+ and CD8+ T-cells (H, I). Mean ± SEM; t-test *=p<0.05; n=3.

Figure S2

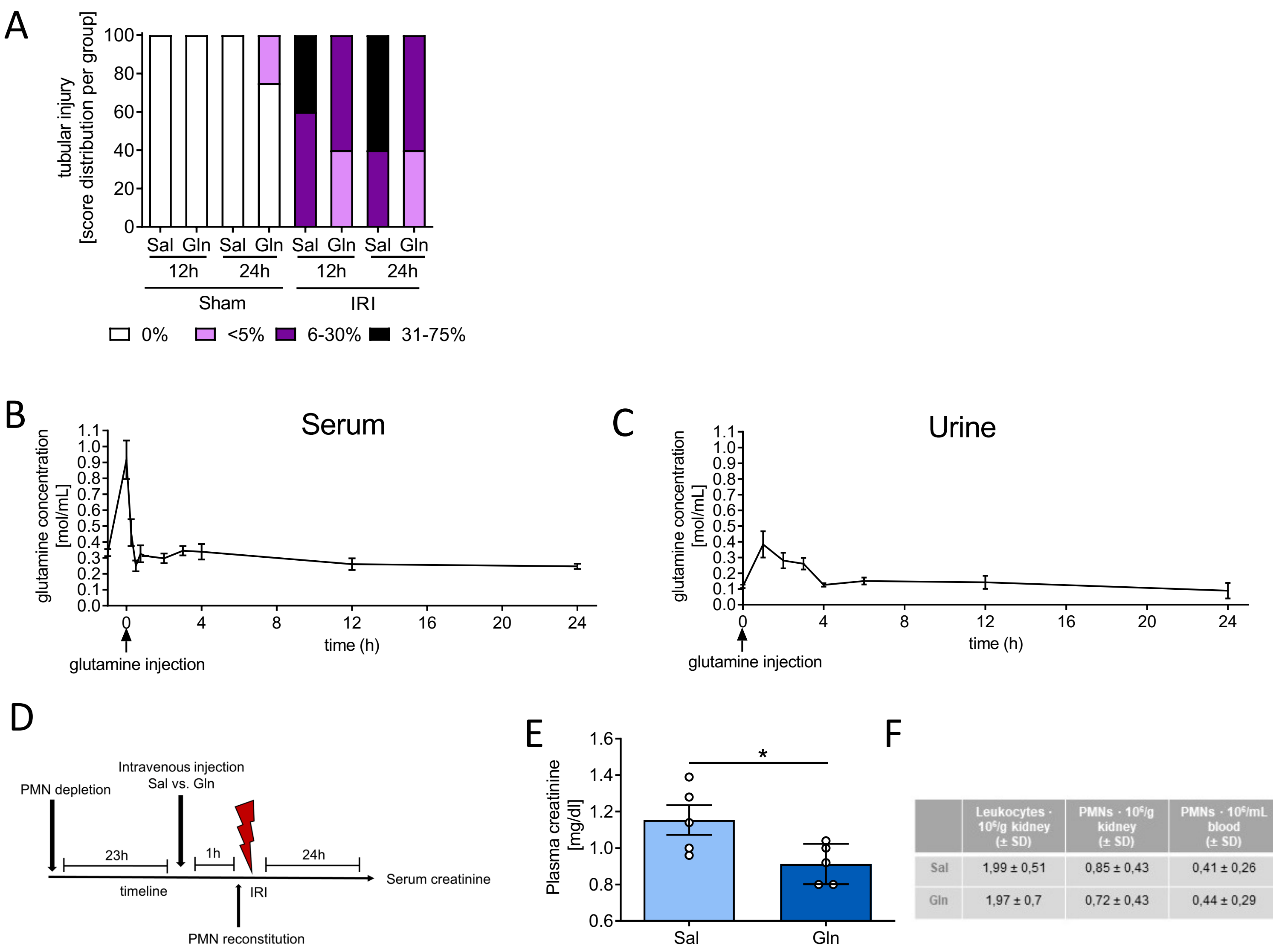

F

	Leukocytes · 10^6/g kidney (± SD)	PMNs · 10^6/g kidney (± SD)	PMNs · 10^6/mL blood (± SD)
Sal	1,99 ± 0,51	0,85 ± 0,43	0,41 ± 0,26
Gln	1,97 ± 0,7	0,72 ± 0,43	0,44 ± 0,29

Figure S2: Glutamine affects renal tubular epithelial cells leading to decreased renal injury. Glutamine was administered in WT mice by intravenous glutamine injection (A-C). After performance of H&E staining of paraffin-embedded sections one tissue section per mouse was scored to identify tissue damage by illustrating score distribution (A; n=5). Glutamine concentration was measured by a colorimetric glutamine assay in collected blood plasma (B; n=3) and excreted urine (C; n=3). Glutamine or saline as vehicle control was administered intravenously 23h after PMN depletion achieved by an intraperitoneal injection of Gr1 in WT mice. 1h after systemic glutamine or saline treatment mice were subsequently subjected to IRI surgery (D). Plasma creatinine levels (E; n=5) and leukocyte counts (F; n=5) were determined 24h post IRI induction. Mean ± SEM; one-way ANOVA *=p<0.05; ** p<0.005; ***p<0.001.

Figure S3

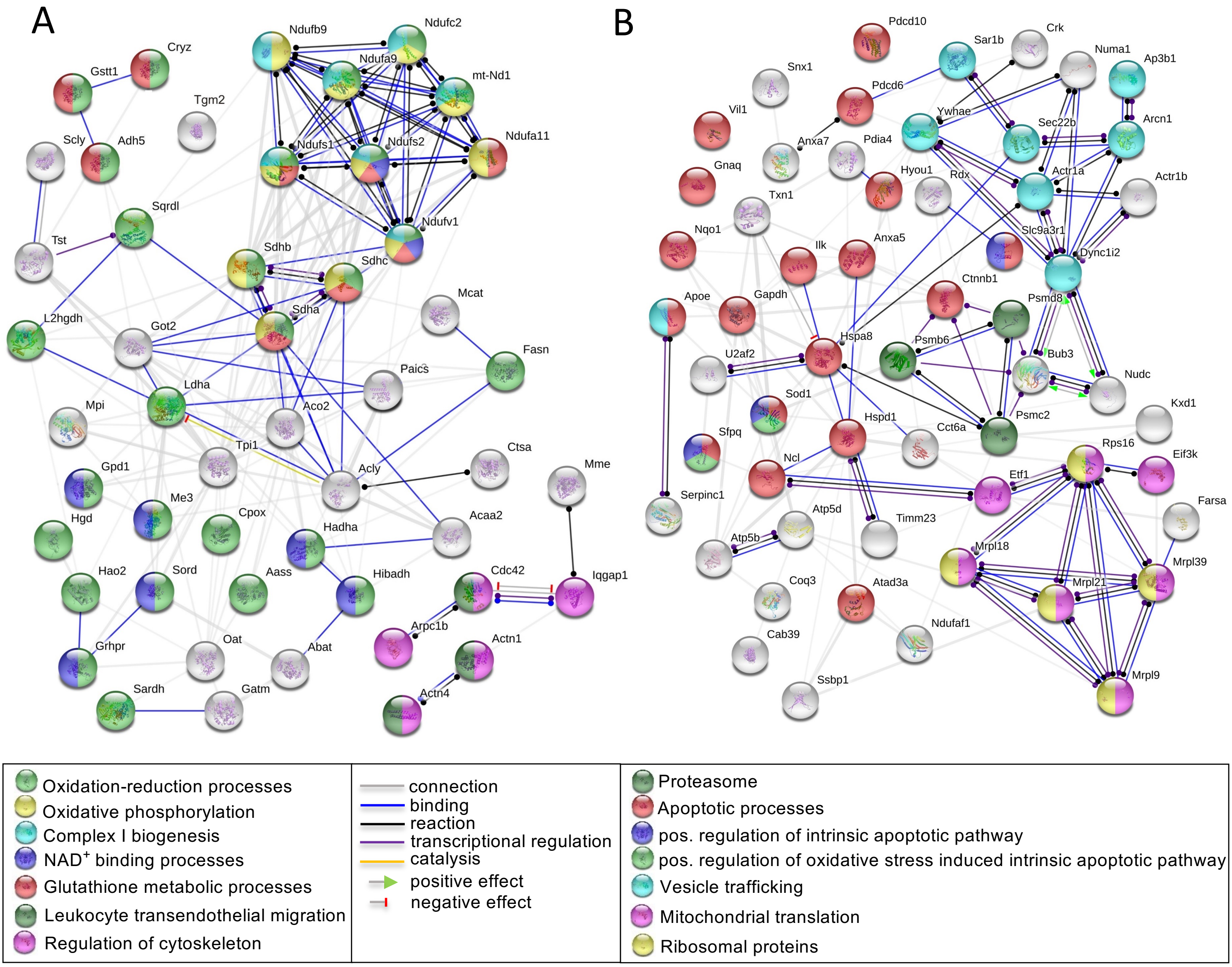

Figure S3: Associated network analysis of regulated genes upon glutamine treatment. WT mice were subjected to sham or IRI surgery and received glutamine or saline 15 min after reperfusion. Kidneys were collected and homogenized 24h after IRI induction. Mass spectrometric label free quantification was performed in order to identify alteration in protein expression levels as a result of glutamine treatment. Association network analysis illustrates upregulated (A) and downregulated proteins (B) by glutamine treatment compared to saline treatment and their mutual connections upon IRI induction. Colors of the different specified proteins represent memberships to the respective processes.

Figure S4

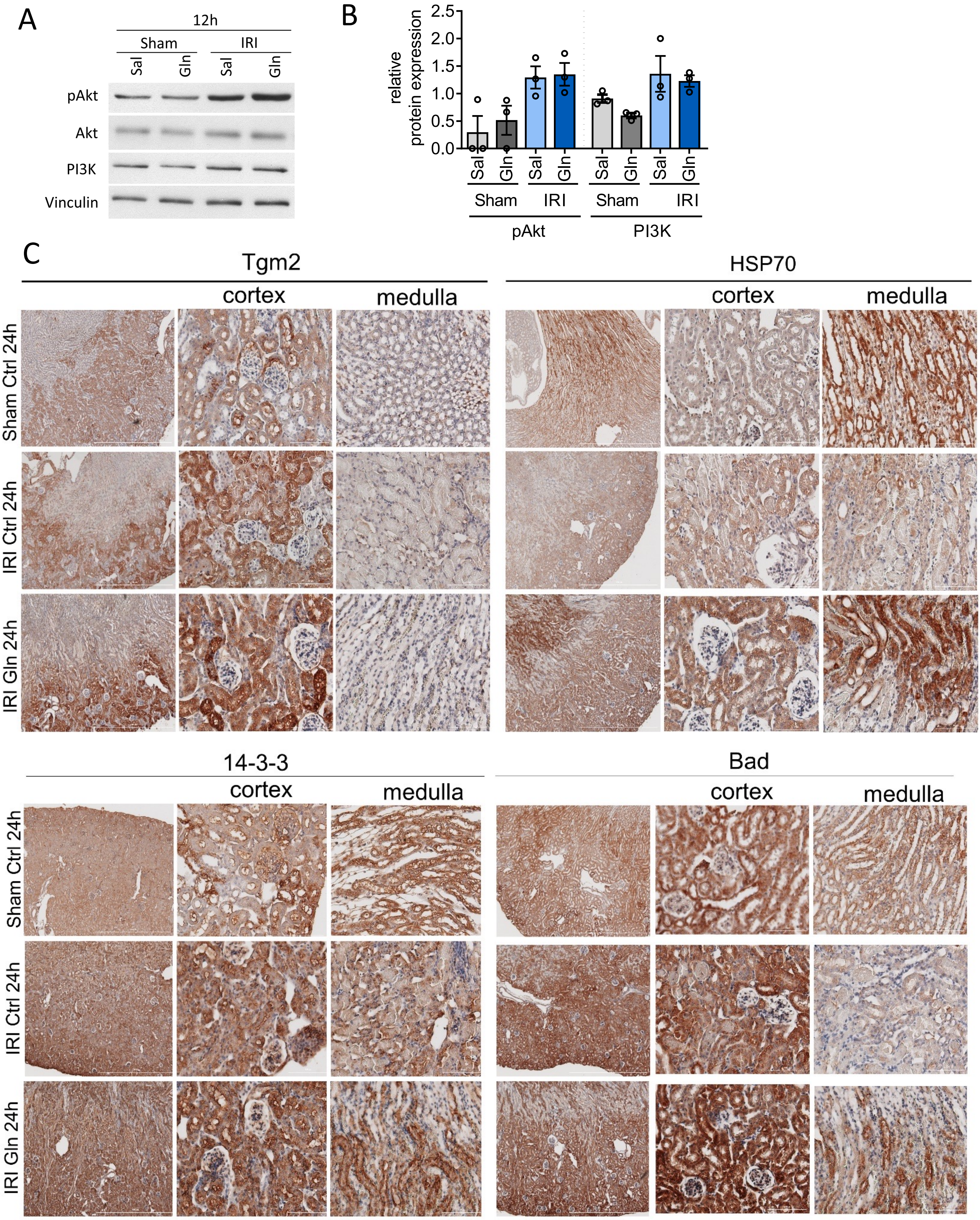

Figure S4: Glutamine administration does not affect PI3K Signaling but enhances transglutaminase 2 and heat shock protein 70 expression. WT mice were subjected to sham or IRI surgery and received glutamine or saline 4h after reperfusion. Analysis of PI3K expression and Akt activation was determined in kidney lysates 12h after IRI induction (A, B). Paraffin embedded tissue sections were prepared and immunohistochemistry staining were performed to illustrate Tgm2, Hsp70, 14-3-3 and Bad protein expression in the kidney (C).

Figure S5

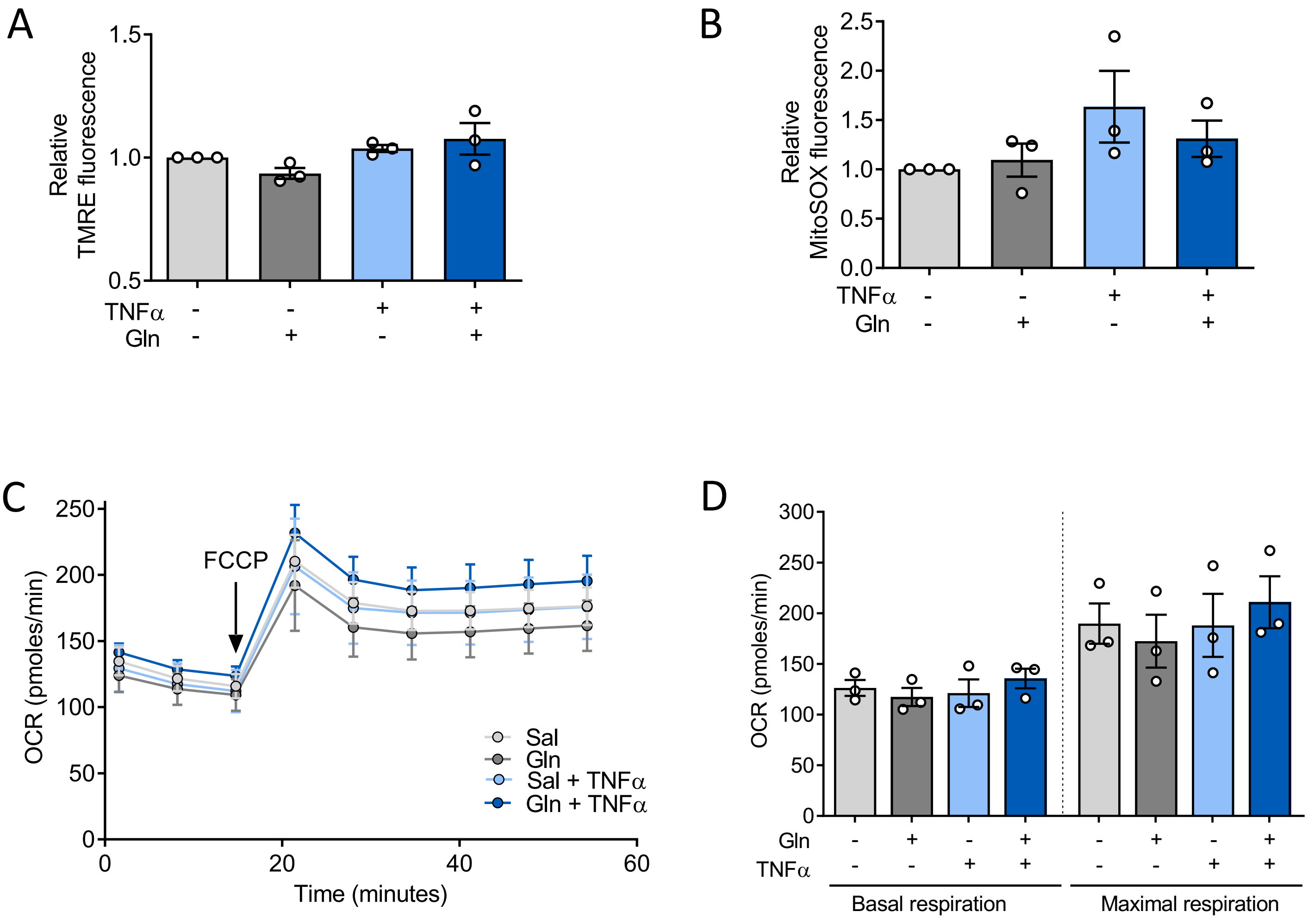

Figure S5: Mitochondrial function in TECs is not affected by TNFα-stimulation or glutamine treatment. TECs were treated with glutamine or saline and subsequently stimulated with 100ng/mL TNFα for 18h. The mitochondrial membrane potential was assessed by TMRE fluorescence detection (A, n=3). Mitochondrial ROS production was detected to assess oxidative stress (B, n=3) . Mitochondrial respiration was assessed using the Seahorse XF24 Flux Analyzer. The oxygen consumption rate (OCR) was measured before (basal respiration) and after FCCP addition (maximal respiration; C,D; n=3). FCCP: Carbonyl cyanide-p-trifluoromethoxyphenylhydrazone. Mean ± SEM; one-way ANOVA.

Figure S6

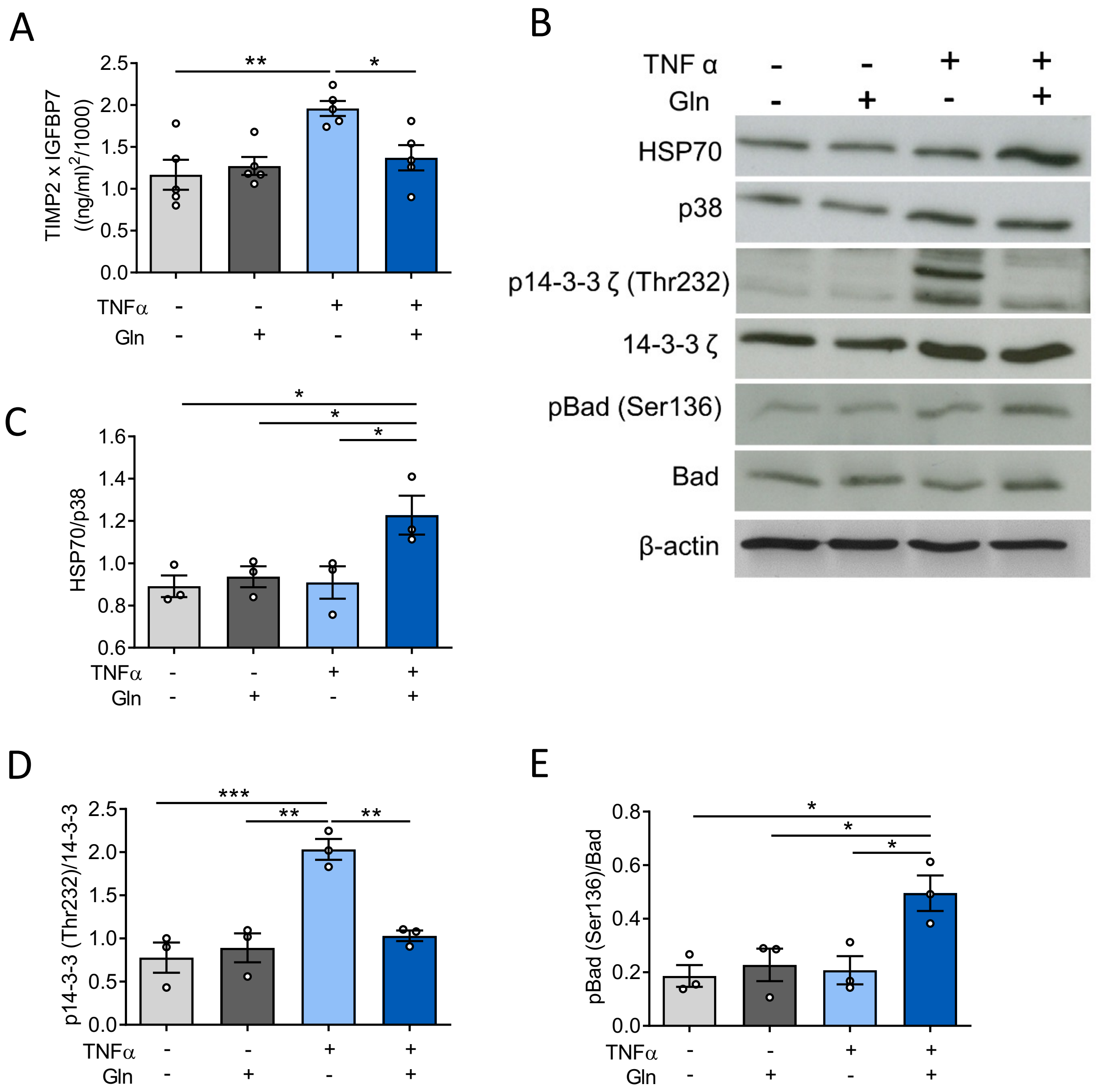

Figure S6: Glutamine administration reduces renal injury by diminishing apoptotic processes in human tubular epithelial cells. Immortalized Human Kidney Epithelial-1 (IHKE-1) cells were treated with glutamine or saline and subsequently stimulated with 30ng/mL TNFα. The supernatant was utilized for TIMP2 and IGFBP7 level determination (A; n=5). IHKE-1 cell lysates were subjected to Western blotting to assess the expression levels of HSP-70 (B, C; n=3), anti-14-3-3ζ and anti-p-14-3-3ζ (Thr232) (B, D; n=3), anti-Bad and anti-p-Bad (Ser136) (B, E; n=3) and β-actin (mean ± SEM; one-way ANOVA *=$p<0.05$; ** $p<0.005$; ***$p<0.001$).

Figure S7

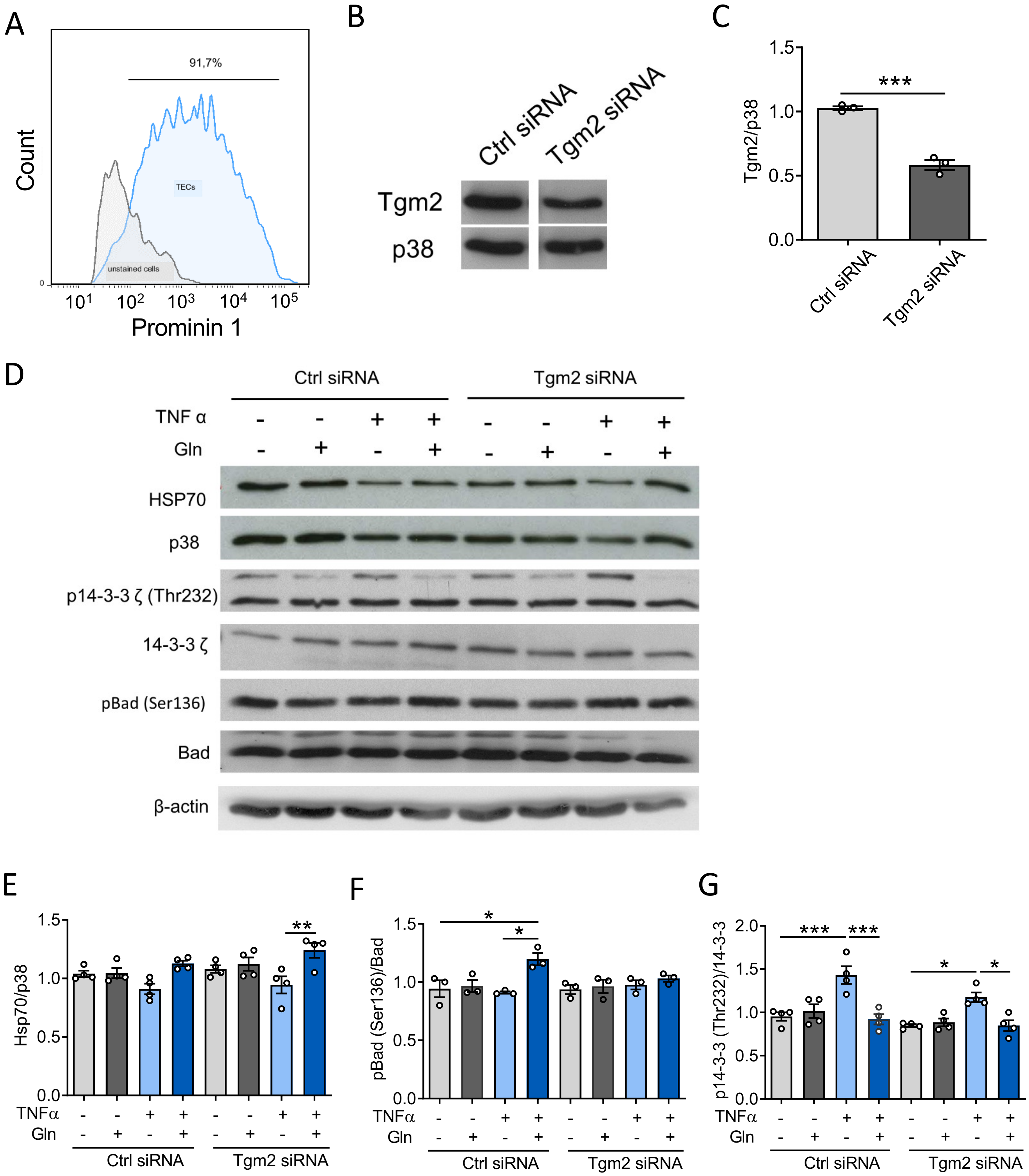

Figure S7: Tgm2 knockdown in kidney tubular epithelial cells modulates apoptotic processes. The purification rate of primary isolated murine TECs was 91.7% (A). TECs were transfected with Tgm2 siRNA and Ctrl siRNA using the Lipofectamine RNAiMAX Reagent. Knockdown efficiency determined by Western blot analysis was ~ 50% (B, C). Transfected TECs were treated with glutamine or saline and subsequently stimulated with 30ng/mL TNFα. TEC lysates were analyzed by Western blotting to assess the expression levels of HSP-70 (D, E; n=3), anti-14-3-3ζ and anti-p-14-3-3ζ (Thr232) (D, F; n=4) as well as anti-Bad and anti-p-Bad (Ser136) (D, G; n=3; mean ± SEM; one-way ANOVA *=p<0.05; ** p<0.005; ***p<0.001).

Figure S8

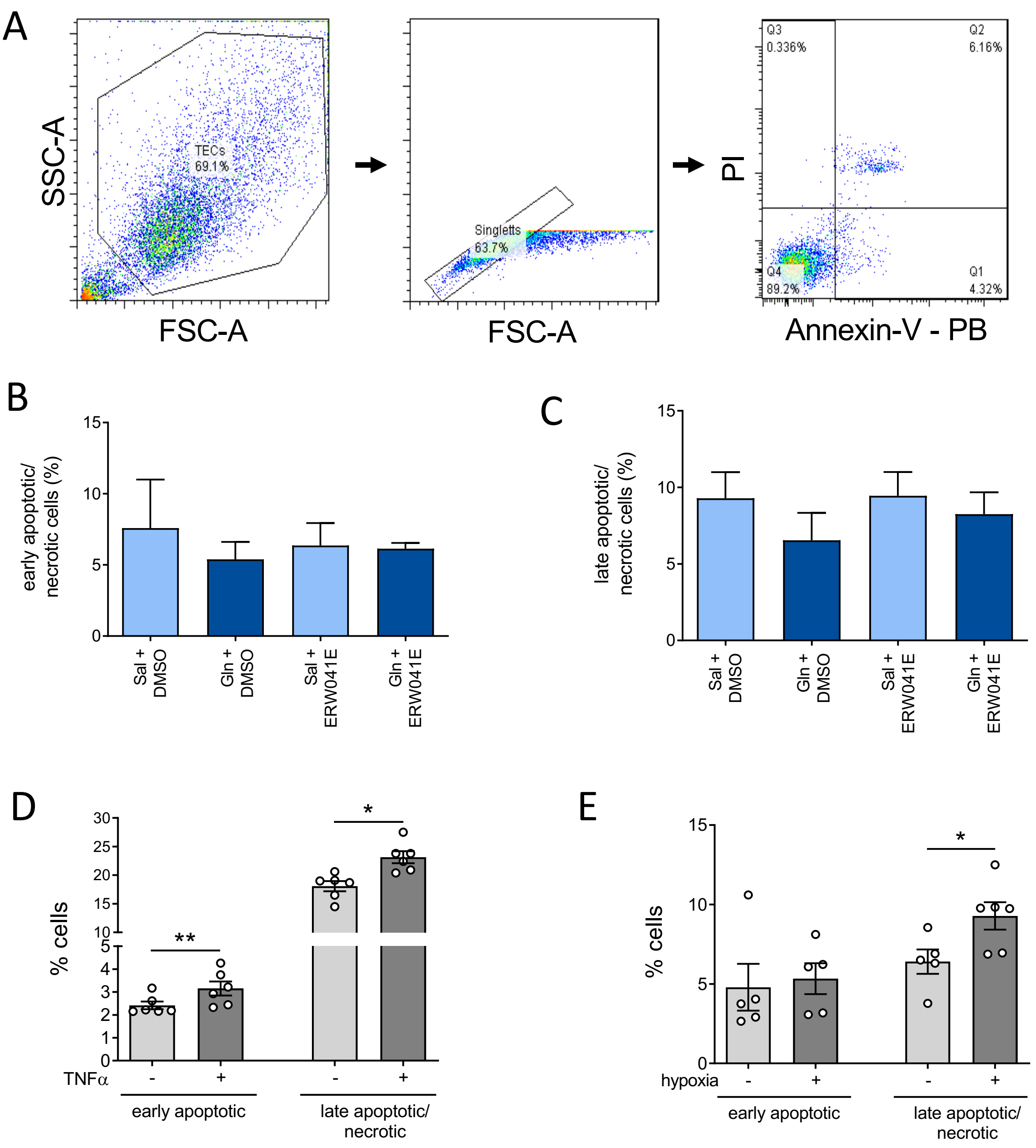

Figure S8: TEC viability after diverse cells treatments. TECs were stained with Pacific Blue™ Annexin V Apoptosis Detection Kit with propidium iodide (PI) after specific treatment with glutamine or saline and DMSO or ERW1041E (Tgm2 inhibitor). Exemplary FACS plots (A) and percentage of early apoptotic (B) and late stage apoptotic/ necrotic endothelial cells (C) (n=3). Percentage of early apoptotic and late stage apoptotic / necrotic TECs after TNFα (D, n=6) and hypoxia induction (E, n=5).

Supplementary Tables

Table S1: List of all identified and quantified proteins of proteomic analysis. WT mice were subjected to sham or IRI surgery and received glutamine or saline 4h after reperfusion. Kidneys were collected and homogenized 24h post reperfusion and mass spectrometric label free quantification was performed in order to detect regulated proteins.

log10LFQ ctrl24IRI_1	log10LFQ ctrl24IRI_2	log10LFQ ctrl24IRI_3	log10LFQ ctrl24IRI_4	log10LFQgln 24IRI_1	log10LFQgln 24IRI_2	log10LFQgln 24IRI_3	log10LFQgln 24IRI_4	t-test Significant	Score	Q-value	Peptides	Number of proteins	Mol. weight [kDa]	(=-Log t-test p value)	t-test Difference	Protein names	Gene names
0,603731	0,372497	0,635281	0,688614	NaN	NaN	-1,84781	-0,45232	+	49,835	0	8	2	21,053	1,763830868	0,3633	Heme-binding protein 1	Hebp1
0,586958	1,467445	0,241937	1,158563	-0,90151	-1,05505	-0,58651	-0,91184	+	33,811	0	10	3	74,194	2,974021689	0,2411	Beta-glucuronidase	Gusb
0,474593	1,233962	0,196415	0,843852	-0,61818	-1,9032	0,371695	-0,59914	+	223,06	0	20	1	76,722	1,42130465	0,2277	Nucleolin	Ncl
0,690754	0,939103	0,894143	0,934003	-0,16964	-0,75648	-0,98062	-1,55127	+	275,6	0	24	2	65,622	2,987912229	0,2218	Acyl-coenzyme A synthetase ACSM3, mitochondrial	Acsm3
0,512417	0,252516	1,053691	1,385673	-0,73238	-1,53427	-0,073	-0,86464	+	12,053	0	6	3	163	2,178447875	0,2185	Receptor-type tyrosine-protein phosphatase kappa	Ptprk
0,099298	1,672394	-0,02089	0,937769	-0,46104	0,152153	-0,83897	-1,54072	+	43,181	0	9	4	56,712	1,35034042	0,2161	Cytochrome P450 2A5	Cyp2a5
0,61365	0,244419	1,449526	0,496765	-1,83714	-0,81845	-0,35706	0,20828	+	17,283	0	4	4	35,866	1,490993121	0,1864	Apolipoprotein E	Apoe
0,532162	0,851748	0,942033	1,246017	-0,52028	-1,34911	-0,7765	-0,92607	+	21,881	0	9	1	38,358	3,647112592	0,1714	Nuclear migration protein nudC	Nudc
-0,11374	1,476712	-0,02062	1,336937	-0,98233	-1,0581	0,284701	-0,92355	+	86,936	0	10	6	20,772	1,339796857	0,1687	Ferritin;Ferritin light chain 1;Ferritin light chain 2	Ftl1;Ftl2
0,193502	1,18833	0,411796	1,20914	-0,66317	-1,56388	0,217305	-0,99302	+	36,954	0	6	3	33,901	1,782157193	0,1677	Splicing factor U2AF 65 kDa subunit	U2af2
0,680786	1,374123	0,741922	0,311658	-0,66187	-1,53529	0,172945	-1,08428	+	126,85	0	33	2	68,542	1,973504781	0,1662	Radixin	Rdx
0,847392	1,487114	0,260638	0,122551	-0,94942	-1,25501	-1,08708	0,573819	+	3,2732	0,001 7857	2	2	35,262	1,384072031	0,1639	Hematopoietic progenitor cell antigen CD34	Cd34
0,973094	0,407136	0,46855	1,108644	-0,05809	-1,97216	-0,70452	-0,22266	+	87,114	0	5	3	17,156	1,708807334	0,1610	Single-stranded DNA-binding protein;Single-stranded DNA-binding protein, mitochondrial	Ssbp1
0,741783	1,017261	0,500419	0,862496	0,090023	-0,89654	-1,83893	-0,47651	+	23,11	0	5	1	40,528	2,000276632	0,1562	Zinc-binding alcohol dehydrogenase domain-containing protein 2	Zadh2
-0,04794	0,572113	0,987215	1,667174	-0,92112	-1,24763	-0,53165	-0,47817	+	174,65	0	20	6	52,003	2,119854361	0,1549	Antithrombin-III	Serpinc1
0,488654	0,574126	0,755362	1,291337	-1,22389	-1,28485	0,357005	-0,95775	+	25,574	0	10	2	55,662	1,97545449	0,1525	Probable carboxypeptidase PM20D1	Pm20d1
1,227787	0,460443	0,048376	1,05915	-0,32256	-1,98297	-0,24378	-0,24643	+	24,525	0	7	3	24,715	1,479908601	0,1459	Programmed cell death protein 10	Pdcd10
0,422179	0,456565	1,010826	1,417324	-1,02693	-1,24923	-0,04826	-0,98247	+	64,504	0	11	3	62,022	2,44512923	0,1436	Bifunctional coenzyme A synthase;Phosphopantetheine adenylyltransferase;Dephospho-CoA kinase	Coasy
-0,0311	1,276189	0,228921	1,263294	-0,42598	-1,76812	0,093651	-0,63686	+	7,0015	0	2	2	25,186	1,407383197	0,1435	Eukaryotic translation initiation factor 4H	Eif4h
NaN	1,410674	0,764646	0,882033	-1,09843	-0,68027	-0,356	-0,92264	+	6,8015	0	3	5	99,657	3,054139267	0,1422		Numa1
0,91786	1,081572	-0,09207	0,901558	-0,29472	-1,94876	-0,03568	-0,52976	+	176,48	0	18	2	51,073	1,496915617	0,1390	Glutathione reductase, mitochondrial	Gsr
0,913992	0,425093	0,733984	0,820791	-0,36066	0,224156	-2,00703	-0,75032	+	20,001	0	7	1	42,158	1,613254023	0,1384	Guanine nucleotide-binding protein G(q) subunit alpha	Gnaq
0,935474	0,725373	0,034243	1,113067	-0,24209	-1,7358	-1,10155	0,271287	+	22,123	0	8	5	85,47	1,495919653	0,1368	Catenin beta-1	Ctnnb1
0,347825	0,847179	0,803195	0,830692	-0,62759	-2,09963	-0,14739	0,045723	+	29,901	0	6	1	42,613	1,52320953	0,1352	Alpha-centractin	Actr1a
0,989309	1,192637	1,112553	0,183444	-1,28105	-0,726	-0,93235	-0,53855	+	8,9972	0	4	1	22,382	3,081089193	0,1341	GTP-binding protein SAR1b	Sar1b
0,331981	1,293173	0,822882	1,035718	-1,30106	-1,04144	-0,71038	-0,43087	+	20,329	0	5	2	21,867	3,109607219	0,1313	Programmed cell death protein 6	Pdcd6
0,234977	0,928712	1,001404	0,687809	-0,65518	-1,96631	0,261117	-0,49254	+	55,253	0	12	1	75,441	1,555661204	0,1304	Splicing factor, proline- and glutamine-rich	Sfpq
-0,16954	1,542408	0,947102	0,55739	-0,24377	-1,53476	-0,12167	-0,97716	+	31,016	0	9	1	30,959	1,589706494	0,1292	NAD(P)H dehydrogenase [quinone] 1	Nqo1
0,531953	-0,36104	0,889891	1,888009	-0,60155	-0,6522	-0,79121	-0,90385	+	24,487	0	8	4	31,458	1,695395021	0,1263	Epimerase family protein SDR39U1	Sdr39u1
1,033816	0,209658	0,507741	0,894725	0,41713	-2,0134	-0,68334	-0,36632	+	31,021	0	3	2	17,6	1,302755699	0,1224	ATP synthase subunit delta, mitochondrial	Atp5d
0,368178	1,573749	NaN	0,886361	-0,25921	-1,26167	-0,86402	-0,44339	+	7,5987	0	2	5	13,637	2,063732447	0,1211	Mesencephalic astrocyte-derived neurotrophic factor	Manf
1,015513	0,911681	0,782615	NaN	-1,01377	-0,67615	-1,01989	NaN	+	3,8185	0,001 3947	2	1	40,956	3,781609458	0,1204	Hexaprenyldihydroxybenzoate methyltransferase, mitochondrial	Coq3
0,276625	1,05217	0,773159	0,8709	-1,1061	-1,77158	0,168877	-0,26406	+	260,84	0	20	4	111,18	1,73324921	0,1200	Hypoxia up-regulated protein 1	Hyou1
1,636919	0,727697	0,411655	0,648727	-0,73094	-1,28869	-0,60751	-0,79786	+	12,323	0	4	10	46,527	2,847459428	0,1198	C-terminal-binding protein 1	Ctbp1
0,497533	0,201915	0,844753	1,407611	-0,1722	-1,8883	-0,33572	-0,5556	+	323,31	0	32	1	56,3	1,700043298	0,1174	ATP synthase subunit beta, mitochondrial	Atp5b
0,471877	0,779154	0,539365	1,27419	0,250972	-1,6158	-1,11198	-0,58778	+	6,3726	0	11	5	43,953	1,890229935	0,1168	3-ketoacyl-CoA thiolase A, peroxisomal	Acaa1a
1,069762	1,256946	0,064499	0,579507	-1,04063	-1,6784	-0,17116	-0,08053	+	94,655	0	12	3	52,27	1,729829421	0,1167	Annexin A7	Anxa7
0,985565	0,405337	1,086666	NaN	NaN	-0,88074	-0,23588	-1,36095	+	3,1982	0,001 7778	3	2	30,839	1,87956551	0,1153	39S ribosomal protein L9, mitochondrial	Mrpl9
0,526134	0,28662	0,999361	1,23494	-0,89366	-1,7084	0,165418	-0,61041	+	290,93	0	24	3	29,174	1,858555708	0,1152	14-3-3 protein epsilon	Ywhae
1,454775	0,953222	1,026171	-0,27646	-0,99041	-1,12548	-0,35474	-0,68708	+	6,9233	0	2	6	55,163	2,074402772	0,1134	COP9 signalosome complex subunit 1	Gps1
0,860108	0,254946	1,149846	0,740273	-1,71322	-1,16511	0,04707	-0,17391	+	20,188	0	6	1	25,378	1,786179673	0,1131	Proteasome subunit beta type-6	Psmb6
0,57087	0,829649	0,380262	1,658043	-0,8352	-0,58333	-0,92813	-1,09217	+	21,112	0	6	2	122,74	2,904382405	0,1122	AP-3 complex subunit beta-1	Ap3b1
0,488631	1,317993	0,399713	0,89959	-0,37168	-1,87423	-0,56119	-0,29883	+	12,88	0	7	1	16,445	1,968481629	0,1118	40S ribosomal protein S16	Rps16
1,101619	0,305887	0,361369	1,451374	-1,22285	-1,27836	-0,17551	-0,54353	+	36,23	0	7	3	57,47	2,216250177	0,1107	Phenylalanine--tRNA ligase alpha subunit	Farsa
1,589986	0,522521	0,548483	0,607713	-1,04878	-1,34018	-0,08932	-0,79043	+	8,7633	0	4	3	52,779	2,338901189	0,1104	Adenylosuccinate synthetase isozyme 1	Adssl1
-0,55881	1,133375	0,743504	1,381199	-0,58925	-1,54081	-0,4569	-0,1123	+	52,935	0	7	6	22,889	1,362604963	0,1088	Adapter molecule crk	Crk
0,272711	0,960852	0,411514	1,203004	-0,47684	-0,68532	0,221846	-1,90777	+	133,24	0	21	1	52,767	1,549070675	0,1082	Cytosolic non-specific dipeptidase	Cndp2
1,36379	0,296405	0,79737	0,742092	0,130553	-0,96315	-1,428	-0,93905	+	18,601	0	6	4	51,373	2,167670789	0,1075	Integrin-linked protein kinase	Ilk
-0,14466	1,554943	-0,12053	1,464026	-0,73582	-0,50232	-0,27772	-1,23792	+	24,659	0	7	2	33,794	1,427349599	0,1060	Phosphopantothenate--cysteine ligase	Ppcs
-0,05851	0,68898	0,606482	1,460555	-0,23423	-1,80114	0,170263	-0,8324	+	23,747	0	7	1	39,842	1,360575305	0,1055	Calcium-binding protein 39	Cab39
1,340444	1,383219	0,49247	-0,28558	-1,13416	-0,85228	-0,97577	0,03166	+	5,6873	0	6	12	58,886	1,667433039	0,1050	Copine-1	Cpne1
1,050048	0,601941	0,352587	1,172603	-0,19468	-1,73757	-0,30326	-0,94167	+	51,678	0	10	1	49,03	2,116788499	0,1046	Eukaryotic peptide chain release factor subunit 1	Etf1
0,983809	-0,33428	1,19175	1,051393	0,16155	-1,43342	-0,82447	-0,79634	+	39,031	0	12	1	38,6	1,611544409	0,1018	Na(+)/H(+) exchange regulatory cofactor NHE-RF1	Slc9a3r1
0,119105	0,162497	0,827202	1,635624	-0,47128	-0,27086	-0,19498	-1,80732	+	166,89	0	13	3	24,205	1,415972849	0,1017	Maleylacetoacetate isomerase	Gstz1
-0,15971	0,704185	1,032512	1,083299	0,33897	-1,86306	-0,50629	-0,6299	+	11,384	0	2	1	43,496	1,318532875	0,1017		Tha1
0,528543	0,260843	1,439932	0,542935	-0,07687	-1,91252	0,00502	-0,78788	+	50,423	0	14	6	66,741	1,450176894	0,1011	ATPase family AAA domain-containing protein 3	Atad3
1,188943	0,602611	0,412097	0,674222	0,124889	-1,46106	-1,56462	0,022926	+	62,818	0	6	7	24,687	1,590425951	0,0981	Acyl-protein thioesterase 1	Lypla1
1,349391	0,493861	NaN	0,619827	0,463544	-1,41416	-0,44593	-1,06653	+	4,7128	0	2	1	38,549	1,3590516	0,0976	39S ribosomal protein L39, mitochondrial	Mrpl39
1,175805	-0,07098	1,313979	0,357752	-0,16775	-1,8536	-0,44386	-0,31134	+	20,087	0	4	1	20,677	1,455560077	0,0974	39S ribosomal protein L18, mitochondrial	Mrpl18
0,686397	0,4883	1,439842	0,284445	-0,32318	-1,50198	0,237504	-1,31133	+	246,57	0	24	2	72,369	1,620667062	0,0972	Protein disulfide-isomerase A4	Pdia4
1,774507	0,078739	0,795775	0,482776	-0,27391	-0,67221	-1,27048	-0,91519	+	6,0644	0	2	2	23,366	2,020219536	0,0970	39S ribosomal protein L21, mitochondrial	Mrpl21
0,246758	1,175295	0,907626	0,637857	0,4172	-1,49063	-1,20907	-0,68504	+	5,3139	0	3	1	23,275	1,724766078	0,0950	SPRY domain-containing protein 4	Spryd4

log10LFQ ctrl24IRI_1	log10LFQ ctrl24IRI_2	log10LFQ ctrl24IRI_3	log10LFQ ctrl24IRI_4	log10LFQgln 24IRI_1	log10LFQgln 24IRI_2	log10LFQgln 24IRI_3	log10LFQgln 24IRI_4	t-test Significant	Score	Q-value	Peptides	Number of proteins	Mol. weight [kDa]	(=-Log t-test p value)	t-test Difference	Protein names	Gene names
0,141283	0,744274	0,30631	1,651571	-0,51259	-0,51044	-0,0663	-1,75411	+	131,6	0	17	1	39,771	1,542758985	0,0924	Alcohol dehydrogenase 1	Adh1
0,403155	0,092356	1,4184	1,324531	-0,36153	-1,128	-1,12967	-0,61924	+	81,612	0	5	11	8,7279	2,260855973	0,0922	Ubiquitin-60S ribosomal protein L40	Uba52;Kxd1;Ubc;Ubb;Rps27a
1,335409	0,175514	0,15429	1,296552	0,193859	-1,22787	-0,76682	-1,16093	+	26,836	0	3	4	9,7982	1,715630772	0,0912	Profilin-2;Profilin	Pfn2
0,731196	0,436509	0,27333	1,493972	-1,62527	-1,13844	-0,17488	0,003587	+	11,466	0	5	3	32,768	1,674193584	0,0910	26S proteasome non-ATPase regulatory subunit 8	Psmd8
0,577619	0,350353	1,023464	1,023134	-0,44668	-1,99968	-0,02792	-0,50029	+	77,114	0	11	1	57,229	1,736000603	0,0902	Coatomer subunit delta	Arcn1
1,116471	0,866621	0,808057	0,241996	-0,64154	-1,89205	0,045815	-0,54537	+	25,154	0	12	3	58,004	1,83401866	0,0897	T-complex protein 1 subunit zeta	Cct6a
1,168225	0,261714	-0,2353	1,49727	0,235508	-1,42455	-0,91796	-0,58491	+	52,853	0	7	1	15,942	1,354153265	0,0896	Superoxide dismutase [Cu-Zn]	Sod1
1,230138	0,492485	1,050892	0,692202	-0,1824	-0,94807	-1,13683	-1,19843	+	150,13	0	15	2	55,028	3,023142	0,0874	Xaa-Pro dipeptidase	Pepd
1,703495	1,181907	-0,19303	0,430797	-0,64472	-0,7921	-0,76141	-0,92495	+	39,69	0	10	3	58,878	2,002724505	0,0862	Sorting nexin-1	Snx1
0,509836	0,8312	0,635209	1,103662	-0,19015	-0,58471	-0,31798	-1,98707	+	14,718	0	7	2	61,51	1,918631916	0,0862	Carboxylesterase 1E	Ces1e
1,53042	0,779243	0,20992	0,25164	0,160995	-1,68539	-1,01036	-0,23647	+	8,6379	0	4	4	21,918	1,448892542	0,0855	Guanylate kinase	Guk1
0,997488	0,5525	1,339553	0,287539	0,0665	-1,53498	-0,88927	-0,81933	+	26,672	0	6	1	11,675	2,116569489	0,0854	Thioredoxin	Txn
-0,26559	0,67379	0,698184	1,776769	-1,36942	-0,77252	-0,16529	-0,57593	+	282,6	0	19	5	35,81	1,597914895	0,0843	Glyceraldehyde-3-phosphate dehydrogenase	Gapdh
0,15413	1,349652	0,976102	0,70702	-1,40615	-1,21263	-0,06852	-0,4996	+	102,31	0	13	2	52,866	2,138537703	0,0834	26S protease regulatory subunit 7	Psmc2
1,229144	-0,32278	1,706326	0,101105	-0,19127	-1,2449	-0,81306	-0,46456	+	89,084	0	18	1	60,82	1,379496027	0,0831	EH domain-containing protein 3	Ehd3
1,090736	0,051431	0,360038	1,260832	0,214577	-1,54486	-1,27731	-0,15544	+	266,38	0	41	3	213,42	1,438730699	0,0820	von Willebrand factor A domain-containing protein 8	Vwa8
-0,21504	0,986344	1,033734	1,103103	-0,11567	-1,5848	-0,13414	-1,07354	+	28,461	0	7	3	42,281	1,634045892	0,0815	Beta-centractin	Actr1b
0,587064	0,323305	1,498382	0,308531	-0,57405	0,006207	-0,18847	-1,96097	+	207,07	0	25	2	61,417	1,383586935	0,0805	Phosphoglucomutase-1	Pgm1;Pgm2
1,29051	0,575822	1,229661	0,086085	-0,60064	-1,56164	-0,7963	-0,2235	+	6,7418	0	3	3	37,782	2,127696111	0,0803	Complex I intermediate-associated protein 30, mitochondrial	Ndufaf1
1,29566	0,711931	1,279609	-0,01048	-1,41077	-0,64506	-0,49808	-0,72281	+	25,812	0	7	5	36,766	2,360449564	0,0792	Mitotic checkpoint protein BUB3	Bub3
0,807653	1,239973	-0,00494	1,001158	-1,46541	-1,21057	0,068302	-0,43617	+	323,31	0	28	2	61,612	1,852853143	0,0760		Ces1f
0,482121	0,406796	0,445717	1,361812	-0,27923	-2,06977	0,077889	-0,42534	+	207,59	0	21	2	35,752	1,359357946	0,0725	Annexin A5	Anxa5
0,583629	0,78428	1,540247	-0,02927	-0,42096	-1,55702	0,125622	-1,02653	+	106,36	0	9	5	68,265	1,591860425	0,0720	Cytoplasmic dynein 1 intermediate chain 2	Dync1i2
1,822756	-0,07542	0,640431	0,306217	-0,37544	-1,66747	-0,45706	-0,19402	+	47,972	0	3	4	6,9459	1,356532692	0,0691	Vesicle-trafficking protein SEC22b	Sec22b
0,055753	1,109352	0,693641	1,381552	-0,67572	-1,36261	-0,18822	-1,01374	+	8,1005	0	3	4	6,088	2,265502908	0,0669	Eukaryotic translation initiation factor 3 subunit K	Eif3k
0,729424	0,232816	0,962499	0,809086	0,08552	0,149848	-1,92895	-1,04025	+	148,45	0	10	10	24,489	1,403209762	0,0633	Ras-related protein Rab-11B;Ras-related protein Rab-11A	Rab11b;Rab11a
0,708971	0,26596	1,172344	0,661393	0,071415	-2,06672	-0,34504	-0,46833	+	323,31	0	46	4	60,955	1,496584062	0,0629	60 kDa heat shock protein, mitochondrial	Hspd1
0,818214	NaN	0,880769	1,063055	-0,94502	-1,41139	0,275381	-0,68101	+	9,422	0	2	2	24,443	1,893780097	0,0613	Uridine diphosphate glucose pyrophosphatase	Nudt14
0,490309	0,183278	1,051776	1,016424	-0,21156	-1,9085	-0,9051	0,28338	+	245,44	0	21	1	44,889	1,412779397	0,0575	Short-chain specific acyl-CoA dehydrogenase, mitochondrial	Acads
0,617633	0,485569	0,197081	1,534839	0,28524	-1,62193	-1,02626	-0,47217	+	323,31	0	21	1	44,816	1,531544522	0,0542	Acetyl-CoA acetyltransferase, mitochondrial	Acat1
1,206903	0,98926	0,784839	-0,30925	-0,86688	-0,98794	0,53809	-1,35503	+	323,31	0	37	3	70,87	1,331323443	0,0488	Heat shock cognate 71 kDa protein	Hspa8
-0,28142	0,334544	1,455372	1,264378	-0,53751	-1,2856	0,116481	-1,06625	+	323,31	0	37	2	92,774	1,450948814	0,0472	Villin-1	Vil1
1,348006	0,791925	0,192354	0,7449	-0,26326	-0,70972	-0,28496	-1,81924	+	17,862	0	2	3	20,749	1,91353446	0,0326	Mitochondrial import inner membrane translocase subunit Tim23	Timm23
-0,80708	-1,46319	-0,68188	0,281452	1,406831	-0,23117	0,240905	1,25414	+	214,41	0	13	2	32,191	1,330144253	-0,0268	Triosephosphate isomerase	Tpi1
NaN	-1,81313	-0,31657	-0,5681	0,321101	0,538202	1,240787	0,597719	+	7,5419	0	2	5	23,143	1,752334667	-0,0452	Lysosomal thioesterase PPT2	Ppt2
-1,43639	NaN	-0,51804	-0,89315	1,067452	1,262533	0,218151	0,299446	+	6,4886	0	4	1	67,098	2,119024838	-0,0470	NADP-dependent malic enzyme, mitochondrial	Me3
-1,60203	0,086677	-0,94211	-0,26071	0,55431	1,325863	1,145002	-0,30701	+	151,61	0	13	3	39,547	1,38462815	-0,0517	Alcohol dehydrogenase class-3	Adh5
0,305604	-1,62363	-0,47072	-0,86812	-0,32802	0,893715	0,727444	1,363722	+	206,05	0	27	5	56,451	1,314750519	-0,0520	4-aminobutyrate aminotransferase, mitochondrial	Abat
-0,69449	-1,3335	-0,87826	-0,11331	0,770498	1,255692	-0,30688	1,300256	+	19,816	0	8	2	38,822	1,810549646	-0,0534	ATPase Asna1	Asna1
0,106259	-0,28503	-1,6472	-0,96909	1,196472	0,019887	0,252262	1,326449	+	23,07	0	6	6	65,936	1,479030274	-0,0548	Phosphoenolpyruvate carboxykinase [GTP], mitochondrial	Pck2
-1,01947	-1,02703	-1,04721	0,141749	0,504217	1,770172	0,63193	0,045636	+	242,7	0	21	17	36,498	1,700266497	-0,0569	L-lactate dehydrogenase;L-lactate dehydrogenase A chain	Ldha
NaN	-0,72369	-0,57242	-1,43887	1,481759	0,842391	-0,00511	0,415941	+	16,937	0	9	2	82,955	1,831767249	-0,0581	Protein transport protein Sec23A	Sec23a
-0,6446	-1,8237	-0,19627	-0,25269	0,782899	1,510113	0,496531	0,127718	+	218,06	0	28	2	102,97	1,647514346	-0,0600	Alpha-aminoadipic semialdehyde synthase, mitochondrial;Lysine ketoglutarate reductase;	Aass
-1,19183	-1,64069	0,57806	-0,57839	1,064959	0,422692	0,646009	0,699189	+	236,43	0	21	1	38,249	1,528494581	-0,0612	Sorbitol dehydrogenase	Sord
-1,72385	-1,38215	-0,06614	0,484769	0,880329	0,502987	0,665825	0,638228	+	191,66	0	9	4	27,974	1,34896796	-0,0613	Isoamyl acetate-hydrolyzing esterase 1 homolog	Iah1
-1,14922	-1,3445	-0,46758	-0,50247	0,640561	1,386696	0,851496	0,585007	+	129,92	0	14	4	93,354	3,014105808	-0,0621	Ubiquitin carboxyl-terminal hydrolase;Ubiquitin carboxyl-terminal hydrolase 5	Usp5
-1,27616	0,668257	-0,99386	-1,08271	1,06242	1,162952	0,528098	-0,069	+	25,583	0	9	1	206,23	1,345667821	-0,0625	Plexin-B2	Plxnb2
-1,12761	-1,13953	-0,44994	-0,43586	1,526536	1,133097	-0,14171	0,635016	+	70,388	0	14	4	176,43	2,064239744	-0,0680	UDP-glucose:glycoprotein glucosyltransferase 1	Uggt1
-0,71584	-0,80072	-0,59132	-1,37527	0,448062	0,766929	0,784693	1,483474	+	85,821	0	15	1	49,714	3,106654967	-0,0687	Oxygen-dependent coproporphyrinogen-III oxidase, mitochondrial	Cpox
-0,42258	-1,68001	-0,31304	-0,41079	1,747255	0,377637	-0,03044	0,731981	+	78,321	0	15	4	47,32	1,519937889	-0,0700	Kynurenine--oxoglutarate transaminase 3	Ccbl2
-1,31148	-1,34058	-0,28022	-0,10456	1,575162	0,521756	0,155068	0,784856	+	63,254	0	17	1	70,35	1,840488818	-0,0711	Bifunctional 3-phosphoadenosine 5-phosphosulfate synthase 2;Sulfate adenylyltransferase;	Papss2
-0,5791	-0,88654	-0,69009	-1,15768	0,712366	1,516514	-0,03517	1,119699	+	249,05	0	36	4	188,74	2,464223933	-0,0720	Ras GTPase-activating-like protein IQGAP1	Iqgap1
-0,23008	-0,96694	-1,06941	-1,13573	0,505989	1,07231	0,407213	1,416645	+	120,04	0	21	3	120,79	2,758762825	-0,0723	ATP-citrate synthase	Acly
-0,63748	-1,14777	-0,82983	-0,89205	0,922686	0,639145	1,538287	0,407024	+	23,799	0	5	2	54,205	3,231379446	-0,0738	Serine/threonine-protein phosphatase;Serine/threonine-protein phosphatase 5	Ppp5c
-0,19685	-0,8307	-0,33881	-1,38745	0,093189	0,381987	0,278881	1,999753	+	303,25	0	29	1	47,411	1,427387487	-0,0753	Aspartate aminotransferase, mitochondrial	Got2
-1,01491	0,180332	-0,84693	-1,00916	1,239963	-0,65913	1,100218	1,009611	+	40,038	0	6	1	29,758	1,352724411	-0,0785	Metaxin-2	Mtx2
-0,39939	-0,42212	-1,93739	-0,10478	1,317144	0,189007	0,357993	0,999539	+	85,364	0	14	3	50,282	1,570541226	-0,0866	Sulfide:quinone oxidoreductase, mitochondrial	Sqrdl
-1,66699	-0,01024	-1,06604	-0,02929	0,183772	0,1254	1,236905	1,226493	+	193,83	0	12	11	43,008	1,450572459	-0,0890	Protein NDRG1	Ndrg1
-0,08855	-0,34863	-1,57423	-1,12046	0,575889	0,271913	0,894103	1,389961	+	130,36	0	18	4	52,625	2,020360792	-0,0899	NADH dehydrogenase [ubiquinone] iron-sulfur protein 2, mitochondrial	Ndufs2
-1,65813	-0,22207	-0,72133	-0,75291	0,495849	0,735367	0,870057	1,253177	+	61,408	0	13	10	37,331	2,591752393	-0,0906	Guanine nucleotide-binding protein G(I)/G(S)/G(T) subunit beta-2	Gnb2
-1,66889	0,126688	-1,01329	-0,21634	1,298664	0,511571	1,078566	-0,11698	+	24,163	0	6	3	42,516	1,449642526	-0,0908	Eukaryotic translation initiation factor 3 subunit M	Eif3m
-1,66707	-0,33862	-0,41667	-0,7874	0,332534	1,035221	1,386749	0,455259	+	306,08	0	24	2	35,231	2,191264941	-0,0911	Glycerol-3-phosphate dehydrogenase [NAD(+)], cytoplasmic	Gpd1
-1,41657	-0,85505	-0,90066	-0,25536	1,250496	0,501377	0,549259	1,126507	+	164,38	0	15	3	35,328	2,858128488	-0,0927	Glyoxylate reductase/hydroxypyruvate reductase	Grhpr
0,593326	-1,30909	-1,06217	-0,87691	1,269515	0,391952	-0,12716	1,120539	+	107,99	0	13	1	31,814	1,312524302	-0,0952	Succinate dehydrogenase [ubiquinone] iron-sulfur subunit, mitochondrial	Sdhb

log10LFQ ctrl24IRI_1	log10LFQ ctrl24IRI_2	log10LFQ ctrl24IRI_3	log10LFQ ctrl24IRI_4	log10LFQgln 24IRI_1	log10LFQgln 24IRI_2	log10LFQgln 24IRI_3	log10LFQgln 24IRI_4	t-test Significant	Score	Q-value	Peptides	Number of proteins	Mol. weight [kDa]	(=-Log t-test p value)	t-test Difference	Protein names	Gene names
-1,45285	-0,46957	-0,74525	-0,63145	1,48445	0,604245	1,059819	0,150608	+	45,014	0	9	4	46,284	2,422749816	-0,0958	COP9 signalosome complex subunit 4	Cops4
-0,98352	-0,30022	-0,85026	-0,90105	1,520878	1,434807	0,167783	-0,08841	+	175,2	0	23	2	137,61	1,837358421	-0,0965	5-oxoprolinase	Oplah
-0,96146	-0,84712	-1,55073	0,459425	0,660938	0,407863	1,396442	0,434641	+	61,77	0	5	1	31,062	1,621975482	-0,0967	S-methyl-5-thioadenosine phosphorylase	Mtap
-1,12436	-0,81267	-0,69971	-0,89836	0,222978	1,323164	0,982119	1,006832	+	88,972	0	13	3	47,006	3,394241811	-0,0972	Multifunctional protein ADE2;Phosphoribosyl-aminoimidazole-succinocarboxamide synthase	Paics
-0,60693	-1,10665	-0,66296	-1,10355	0,629433	1,313742	0,295993	1,240926	+	323,31	0	44	3	85,462	3,091577245	-0,0984	Aconitate hydratase, mitochondrial	Aco2
-1,22486	0,307563	-1,81433	-0,11685	0,556766	0,720931	0,650725	0,920055	+	38,454	0	6	4	15,115	1,54961102	-0,0998	NADH dehydrogenase [ubiquinone] 1 alpha subcomplex subunit 11	Ndufa11
0,210275	-0,76762	-1,14385	-1,1982	0,733725	1,728724	0,208978	0,227965	+	15,583	0	5	2	29,736	1,621260362	-0,1023	Syntaxin-7	Stx7
NaN	-0,98587	-0,94825	-0,95879	0,525243	0,382657	0,35331	1,631705	+	31,389	0	3	2	35,377	2,261938478	-0,1037	Aminoacyl tRNA synthase complex-interacting multifunctional protein 2	Aimp2
-1,31217	-0,9197	-0,51687	-0,26918	1,045176	-0,35784	0,885558	1,445026	+	75,409	0	5	2	35,334	1,807730766	-0,1041	Elongation factor Ts, mitochondrial	Tsfm
NaN	-1,05686	-0,93567	-0,85565	1,119233	0,156854	1,403463	0,16863	+	9,8641	0	4	4	41,928	2,12078847	-0,1047	Malonyl-CoA-acyl carrier protein transacylase, mitochondrial	Mcat
-1,30303	-1,27559	-0,7404	0,258301	0,878625	0,595418	1,374205	0,212464	+	276,71	0	17	9	35,268	1,883158225	-0,1057	Quinone oxidoreductase	Cryz
-1,18519	-1,19104	-0,85627	0,314655	1,399899	0,691651	-0,11838	0,944682	+	17,336	0	3	1	48,34	1,648387516	-0,1058	39S ribosomal protein L37, mitochondrial	Mrpl37
-0,16004	-1,14981	-0,57032	-1,23987	0,782435	1,058226	-0,14751	1,426879	+	287,64	0	28	1	82,669	1,996411528	-0,1065	Trifunctional enzyme subunit alpha, mitochondrial;Long-chain enoyl-CoA hydratase;	Hadha
-0,22884	-0,89109	-0,38756	-0,94041	NaN	1,826996	-0,2319	0,852821	+	7,0912	0	4	3	68,6	1,338338397	-0,1087	Inhibitor of carbonic anhydrase	1300017J02Rik;Ica
-0,67192	-1,39751	-0,52967	-0,75413	0,996968	1,560013	0,369525	0,426725	+	139,81	0	19	2	49,959	2,587766897	-0,1097	Homogentisate 1,2-dioxygenase	Hgd
0,235471	-0,75912	-1,38014	-0,90937	0,521145	1,650001	0,788113	-0,14611	+	9,9756	0	3	2	58,752	1,502436552	-0,1118	Intercellular adhesion molecule 1	Icam1
0,146389	-0,59699	-0,65308	-1,76789	0,775513	1,138818	-0,11876	1,076006	+	323,31	0	33	2	72,585	1,581559197	-0,1127	Succinate dehydrogenase [ubiquinone] flavoprotein subunit, mitochondrial	Sdha
-0,9181	-0,56409	-1,37248	-0,23339	-0,24107	0,795616	1,278179	1,255336	+	17,847	0	9	3	27,374	1,934024718	-0,1128	Glutathione S-transferase theta-1	Gstt1
-0,47158	-1,92647	0,000876	-0,59246	1,109106	0,697617	0,204872	0,978036	+	132,4	0	13	1	48,354	1,760422988	-0,1129	Ornithine aminotransferase, mitochondrial	Oat
-0,8537	-0,51195	-1,60216	0,289393	0,927282	1,440277	-0,27808	0,588942	+	6,6349	0	2	1	31,775	1,338818449	-0,1163	Sodium/potassium-transporting ATPase subunit beta-3	Atp1b3
-1,39007	-1,18862	-0,00617	-0,76181	0,650263	0,996554	1,176855	0,523005	+	34,149	0	11	1	41,793	2,566559958	-0,1176	Arsenite methyltransferase	As3mt
-0,91141	-1,41592	-0,56958	-0,00158	-0,10151	0,262731	1,283741	1,453531	+	6,3452	0	2	2	13,545	1,619956614	-0,1181	SH3 domain-binding glutamic acid-rich-like protein 2	Sh3bgrl2
-0,5034	-0,14376	-1,64038	-0,78408	0,079769	0,497214	1,413275	1,081366	+	22,393	0	6	5	41,498	1,903191633	-0,1197	Actin-related protein 2/3 complex subunit 1B	Arpc1b
-0,45449	0,252307	-1,60526	-0,93744	0,690327	-0,26829	1,043578	1,279269	+	300,67	0	22	6	49,913	1,416530104	-0,1232	NADH dehydrogenase [ubiquinone] flavoprotein 1, mitochondrial	Ndufv1
-1,43726	-1,34632	0,317873	-0,2806	1,397481	0,450825	0,886333	0,011671	+	213,78	0	19	9	36,876	1,418253048	-0,1253	Aldo-keto reductase family 1 member C21	Akr1c21
-0,37203	-1,48042	-0,70121	-0,78075	0,483285	0,308994	1,205971	1,336149	+	286,95	0	14	1	35,44	2,527788646	-0,1273	3-hydroxyisobutyrate dehydrogenase, mitochondrial	Hibadh
-0,04947	-0,51353	-1,25172	-1,47958	0,846801	0,870853	0,428091	1,148554	+	323,31	0	42	6	79,776	2,40906347	-0,1285	NADH-ubiquinone oxidoreductase 75 kDa subunit, mitochondrial	Ndufs1
-0,99975	-0,75903	-1,04023	0,028249	-0,53455	0,576451	1,372424	1,356436	+	34,223	0	5	6	38,044	1,448312764	-0,1297	GTPase IMAP family member 4	Gimap4
-0,38117	-1,19177	-0,92226	-0,4822	0,300222	-0,20554	1,262546	1,620161	+	66,183	0	6	1	22,417	1,740539217	-0,1320	Isochorismatase domain-containing protein 2A, mitochondrial	Isoc2a
-1,12667	-1,35442	NaN	NaN	0,135841	0,613585	0,896655	0,835007	+	8,597	0	2	2	81,11	2,645718664	-0,1322	Cullin-3	Cul3
-0,78485	-0,95123	-0,78339	-0,87829	1,033116	1,616792	0,115957	0,631895	+	143,35	0	13	3	37,377	2,742392142	-0,1327	Guanine nucleotide-binding protein G(I)/G(S)/G(T) subunit beta-1	Gnb1
-0,65924	-0,29267	-0,94811	-0,92098	-0,06495	1,933908	-0,04129	0,993335	+	21,799	0	4	11	21,258	1,512741782	-0,1342	Cell division control protein 42 homolog	Cdc42
-0,58122	-0,09268	-1,52117	-0,90877	0,385253	1,394895	0,140555	1,18315	+	77,919	0	16	2	42,121	1,964433153	-0,1346	NADH dehydrogenase [ubiquinone] 1 alpha subcomplex subunit 9, mitochondrial	Ndufa9
NaN	-1,17314	NaN	-0,9923	0,670384	1,403188	0,37496	-0,2831	+	18,787	0	4	2	57,788	1,432485234	-0,1355	Protein ERGIC-53	Lman1
-0,87987	-1,56639	-0,52344	-0,05279	1,393925	0,236846	0,182082	1,209633	+	95,907	0	10	1	33,466	1,815556336	-0,1379	Thiosulfate sulfurtransferase	Tst
-0,83778	-1,45197	-0,26278	NaN	0,833006	1,367409	-0,32151	0,67363	+	13,105	0	5	2	46,575	1,491112354	-0,1384	Mannose-6-phosphate isomerase	Mpi
-0,50005	-1,63887	-0,82154	-0,34937	0,795825	0,893842	1,321119	0,299046	+	14,205	0	5	9	47,203	2,453695703	-0,1389	Selenocysteine lyase	Scly
-0,77727	-1,25632	0,032002	-0,76344	1,110917	1,433234	-0,56815	0,789023	+	90,587	0	11	4	41,708	1,441191607	-0,1483	3 beta-hydroxysteroid dehydrogenase type 4	Gm4450;Hsd3b4
-0,36	0,060261	-1,65732	-0,82621	0,695283	1,250041	-0,30225	1,140199	+	57,329	0	9	3	29,497	1,464022418	-0,1484	Cytochrome c1, heme protein, mitochondrial	Cyc1
-0,20814	-0,99294	-0,27388	-1,23812	0,6746	1,893518	-0,32237	0,467321	+	45,313	0	5	5	122,29	1,378644947	-0,1490	Calcium-transporting ATPase	Atp2b4
-0,60907	0,065491	-1,28889	-1,32514	0,622731	1,132522	0,174961	1,227396	+	11,168	0	3	5	14,364	2,074186561	-0,1502	60S ribosomal protein L18a	Rpl18a
-1,03494	-1,22752	-0,59661	-0,73163	0,701081	1,246287	0,561007	1,082317	+	56,507	0	7	1	16,318	3,79785768	-0,1534	Cytochrome b5 type B	Cyb5b
-0,36576	-1,29265	-0,12038	-0,99026	0,93353	0,533804	-0,39712	1,698835	+	244,38	0	25	1	48,297	1,446180479	-0,1560	Glycine amidinotransferase, mitochondrial	Gatm
-0,26863	-1,21431	-0,08119	-1,60304	1,11319	1,023568	0,343129	0,687281	+	154,91	0	22	4	64,76	2,094807283	-0,1595	Acyl-coenzyme A synthetase ACSM1, mitochondrial	Acsm1
-0,78802	-0,96253	-0,86356	-0,56559	1,680543	-0,11902	0,463185	1,15499	+	70,542	0	5	2	26,34	2,122391594	-0,1599	Lysophospholipase-like protein 1	Lyplal1
-0,78802	-0,96253	-0,86356	-0,56559	1,680543	-0,11902	0,463185	1,15499	+	70,542	0	5	2	26,34	2,122391594	-0,1599	Lysophospholipase-like protein 1	Lyplal1
-1,05072	-0,74393	-0,65009	-0,91582	0,98163	0,715103	0,05022	1,613605	+	73,855	0	10	4	55,726	2,611917201	-0,1602	Carboxypeptidase;Lysosomal protective protein;Lysosomal protective protein 32 kDa chain;Lysosomal protective protein 20 kDa chain	Ctsa
-0,78776	-0,51506	-1,25101	-0,88626	0,483338	1,45567	0,373418	1,127665	+	22,092	0	6	2	14,164	2,909736277	-0,1654	NADH dehydrogenase [ubiquinone] 1 subunit C2	Ndufc2
-0,98057	-0,84608	-1,144	-0,54068	0,734715	1,094513	1,374467	0,307633	+	14,774	0	6	17	90,202	3,254480844	-0,1658	Band 4.1-like protein 3;Band 4.1-like protein 3, N-terminally processed	Epb4.1l3;Epb41l3
-0,29131	-1,24324	-0,91941	-0,28182	0,488367	2,016955	0,351437	-0,12098	+	51,882	0	6	5	85,701	1,405552262	-0,1715	Neprilysin	Mme
0,335188	-1,248	-1,23059	-0,86772	1,099472	0,433563	0,203456	1,274622	+	36,152	0	5	1	12,097	1,796177352	-0,1781	CDGSH iron-sulfur domain-containing protein 1	Cisd1
-0,65039	-1,1464	-0,68967	-0,82177	0,658277	0,219639	0,633344	1,796972	+	323,31	0	28	1	41,829	2,449022598	-0,1795	3-ketoacyl-CoA thiolase, mitochondrial	Acaa2
0,202088	-1,33419	-0,47471	-1,08103	0,536301	1,78598	-0,16432	0,529883	+	17,284	0	3	1	25,07	1,349508595	-0,1861	Collectrin	Tmem27
0,172298	-1,03371	-0,90656	-0,92684	1,09064	1,737234	-0,01884	-0,11422	+	323,31	0	73	7	282,34	1,357476577	-0,1898	Spectrin alpha chain, non-erythrocytic 1	Sptan1
-0,5289	-0,23013	-0,88764	-1,2306	0,643851	1,95077	-0,08783	0,370481	+	15,72	0	6	3	18,382	1,589576308	-0,1954	Succinate dehydrogenase cytochrome b560 subunit, mitochondrial	Sdhc
-1,76699	-0,64027	-0,75049	-0,09605	0,781192	1,096979	0,948148	0,427475	+	45,217	0	7	1	21,984	2,299826632	-0,1956	NADH dehydrogenase [ubiquinone] 1 beta subcomplex subunit 9	Ndufb9
-0,04725	-1,21375	-0,91297	-0,88723	0,622647	1,132531	-0,17787	1,483893	+	14,266	0	6	1	48,696	1,884052338	-0,2012	Protein amnionless	Amn
0,07258	-1,24702	-0,775	-0,86728	1,566725	0,850422	-0,4441	0,843679	+	55,353	0	8	6	44,331	1,507114014	-0,2269	Acyl-coenzyme A amino acid N-acyltransferase 1;Acyl-coenzyme A amino acid N-acyltransferase 2	Acnat1;Acnat2
-0,98057	-0,2081	-0,88234	-0,61438	0,800698	1,998677	-0,37073	0,256746	+	15,598	0	5	1	36,059	1,346718056	-0,2307	NADH-ubiquinone oxidoreductase chain 1	Mtnd1
-0,50555	-1,53409	-0,54343	-0,53452	0,098113	0,443545	0,909909	1,666029	+	56,744	0	10	1	38,699	1,99154113	-0,2352	Hydroxyacid oxidase 2	Hao2
-0,72249	-1,15027	-0,57836	-0,63457	-0,44953	1,133683	1,047887	1,353641	+	49,258	0	12	1	50,898	1,92951906	-0,2540	L-2-hydroxyglutarate dehydrogenase, mitochondrial	L2hgdh
-0,33061	-0,80504	-0,04414	-1,51798	0,093516	1,205201	-0,16548	1,564529	+	121,39	0	22	2	272,43	1,360880878	-0,2554	Fatty acid synthase;	Fasn
-0,66084	-0,86235	-1,00577	-0,99479	0,826424	1,426182	0,252415	1,018722	+	145,15	0	16	2	77,06	3,325557615	-0,2554	Protein-glutamine gamma-glutamyltransferase 2	Tgm2
-0,44114	-1,05759	-0,74498	-1,22016	1,554598	0,579266	0,608808	0,721203	+	185,86	0	36	3	101,68	3,014639834	-0,2713	Sarcosine dehydrogenase, mitochondrial	Sardh

log10LFQ ctrl24IRI_1	log10LFQ ctrl24IRI_2	log10LFQ ctrl24IRI_3	log10LFQ ctrl24IRI_4	log10LFQgln 24IRI_1	log10LFQgln 24IRI_2	log10LFQgln 24IRI_3	log10LFQgln 24IRI_4	t-test Significant	Score	Q-value	Peptides	Number of proteins	Mol. weight [kDa]	(=-Log t-test p value)	t-test Difference	Protein names	Gene names
-0,71717	-1,39427	0,285435	-1,01814	0,51891	1,621981	-0,02001	0,723261	+	24,602	0	6	1	59,246	1,543719418	-0,2806	Solute carrier family 22 member 8	Slc22a8
-0,53713	-0,95688	-1,19723	-0,94441	0,910479	0,881424	0,634624	1,20912	+	258,17	0	35	10	104,98	4,254495112	-0,3793	Alpha-actinin-4	Actn4
-0,83456	-0,60525	-1,39175	-0,69809	0,870983	1,393719	0,55565	0,709289	+	84,501	0	17	2	102,72	3,360787115	-0,3995	Alpha-actinin-1	Actn1

**Column name**	**Description**
log10LFQctrl24IRI_1	Log10 Intensity of the sample ctrl 24 IRI first biological replicate
log10LFQctrl24IRI_2	Log10 Intensity of the sample ctrl 24 IRI second biological replicate
log10LFQctrl24IRI_3	Log10 Intensity of the sample ctrl 24 IRI third biological replicate
log10LFQctrl24IRI_4	Log10 Intensity of the sample ctrl 24 IRI fourth biological replicate
log10LFQgln24IRI_1	Log10 Intensity of the sample gln 24 IRI first biological replicate
log10LFQgln24IRI_2	Log10 Intensity of the sample gln 24 IRI second biological replicate
log10LFQgln24IRI_3	Log10 Intensity of the sample gln 24 IRI third biological replicate
log10LFQgln24IRI_4	Log10 Intensity of the sample gln 24 IRI fourth biological replicate
t-test Significant	Two sample t-test significance
Score	Protein score which is derived from peptide posterior error probabilities.
Q-value	The ratio of reverse to forward protein groups.
Peptides	The total number of peptide sequences associated with the protein group (i.e. for all the proteins in the group).
Number of proteins	Number of proteins contained within the group. This corresponds to the number of entries in the colum 'Protein IDs'.
Mol. weight [kDa]	Molecular weight of the leading protein sequence contained in the protein group
(=-Log t-test p value)	(-Log10) p value from t-test (ctrl24IRI vs gln24IRI)
t-test Difference	t-test difference (ctrl24IRI vs gln24IRI)
Protein names	Name(s) of protein(s) contained within the group.
Gene names	Name(s) of the gene(s) associated to the protein(s) contained within the group.

Table S2: List of all identified and quantified differentially regulated genes in TECs. WT mice were subjected to sham or IRI surgery and received glutamine or saline 15min after reperfusion. Kidneys were collected and homogenized 24h post reperfusion. Renal TECs were sorted from kidney suspension by FACS based upon the expression of CD45^{-} Prominin-1^{+} and RNA-Seq was performed in order to detect regulated genes.

baseMean	log2FoldChange	lfcSE	svalue	protein names	gene names
16,01	7,97	2,96	9.183887e-04	predicted gene 17126	Gm17126
14,49	7,86	2,90	8.613171e-04	predicted gene, 22460	Gm22460
13,11	7,69	2,89	1.009046e-03	PET117 homolog	Pet117
12,16	7,15	3,20	3.621658e-03	integrin alpha 8	Itga8
10,37	7,05	3,09	2.974900e-03	predicted gene 14288	Gm14288
12,95	5,05	2,00	1.615530e-03	transmembrane protein 61	Tmem61
32,09	4,92	0,92	7.601998e-09	predicted gene, 35240	Gm35240
12,13	4,91	1,69	3.774011e-04	predicted gene 11491	Gm11491
12,41	4,89	2,10	2.701389e-03	RIKEN cDNA 1700124L16 gene	1700124L16Rik
9,33	4,86	1,92	1.579023e-03	predicted gene 28625	Gm28625
15,54	4,84	1,81	9.482936e-04	predicted gene, 28040	Gm28040
12,36	4,67	1,82	1.378013e-03	axonemal dynein light chain domain containing 1	Axdnd1
12,62	4,58	1,67	6.934647e-04	predicted gene 15538	Gm15538
14,47	4,49	1,53	3.672363e-04	poly(A) binding protein, cytoplasmic 4-like	Pabpc4l
21,49	4,29	1,43	3.108966e-04	stabilin 2	Stab2
18,78	4,20	1,26	8.985513e-05	predicted gene 45088	Gm45088
16,42	3,88	1,36	4.817452e-04	small proline-rich protein 2A2	Sprr2a2
15,52	3,76	1,76	4.703496e-03	solute carrier family 26, member 7	Slc26a7
10,69	3,74	1,50	1.856085e-03	predicted gene, 48508	Gm48508
62,78	3,59	0,65	3.701374e-09	apolipoprotein L 9a	Apol9a
49,32	3,58	0,98	2.861549e-05	tRNA aspartic acid methyltransferase 1	Trdmt1
46,88	3,54	0,88	7.806127e-06	predicted gene, 48765	Gm48765
47,04	3,54	0,97	3.277297e-05	myeloid nuclear differentiation antigen like	Mndal
35,51	3,52	1,57	3.414310e-03	RIKEN cDNA 1700110I01 gene	1700110I01Rik
18,63	3,41	1,70	6.972204e-03	unc-79 homolog	Unc79
24,92	3,40	1,14	3.417156e-04	a disintegrin and metallopeptidase domain 22	Adam22
24,31	3,37	0,99	6.665517e-05	olfactory receptor 1233	Olfr1233
34,31	3,21	0,98	1.132874e-04	tetratricopeptide repeat and ankyrin repeat containing 1	Trank1
16,05	3,17	1,41	3.503260e-03	predicted gene, 38009	Gm38009
43,33	3,13	0,77	6.523578e-06	predicted gene 13936	Gm13936
45,99	3,12	0,77	7.366500e-06	serine/threonine/tyrosine kinase 1	Styk1
16,83	3,06	1,45	5.075674e-03	RIKEN cDNA 4921531C22 gene	4921531C22Rik
29,29	3,01	0,98	2.549569e-04	dynein heavy chain domain 1	Dnhd1
13,51	2,76	1,39	7.351053e-03	predicted gene 6313	Gm6313
25,87	2,76	0,91	2.748214e-04	RIKEN cDNA A530058O07 gene	A530058O07Rik
14,37	2,73	1,23	3.972966e-03	predicted gene, 47608	Gm47608
28,29	2,71	1,24	4.218714e-03	RIKEN cDNA A630010A05 gene	A630010A05Rik
45,94	2,68	0,97	6.230687e-04	predicted gene 44660	Gm44660
14,70	2,62	1,23	4.871789e-03	olfactory receptor 66	Olfr66
40,54	2,44	1,11	4.032686e-03	Fanconi anemia, complementation group M	Fancm
48,45	2,42	0,98	1.951958e-03	ankyrin 2, brain	Ank2
19,03	2,42	1,08	3.444035e-03	vomeronasal 2, receptor 73	Vmn2r73
303,30	2,39	0,35	3.607212e-12	sterile alpha motif domain containing 4	Samd4
24,53	2,38	0,88	8.751200e-04	predicted gene, 29683	Gm29683
24,20	2,38	1,12	4.838143e-03	predicted pseudogene 3839	Gm3839
36,92	2,37	0,82	4.606418e-04	kinesin family member 20B	Kif20b
50,34	2,36	0,66	3.918333e-05	RIKEN cDNA 2810429I04 gene	2810429I04Rik
114,18	2,35	0,57	5.374777e-06	TBC1 domain family, member 24	Tbc1d24
134,16	2,34	0,48	1.273893e-07	hydroxy-delta-5-steroid dehydrogenase, 3 beta- and steroid delta-isomerase 2	Hsd3b2
82,79	2,33	0,56	4.985244e-06	methyl-CpG binding domain protein 5	Mbd5
99,55	2,28	0,83	6.793438e-04	olfactory receptor 539	Olfr539
419,46	2,26	0,40	1.817680e-09	predicted gene, 49273	Gm49273
133,44	2,22	0,55	6.938409e-06	olfactory receptor 986	Olfr986
202,57	2,20	0,43	3.106980e-08	predicted gene, 47036	Gm47036
28,60	2,17	0,89	2.118578e-03	janus kinase and microtubule interacting protein 2	Jakmip2
26,06	2,16	0,87	1.893720e-03	predicted gene, 50172	Gm50172
29,65	2,16	0,85	1.487642e-03	predicted gene, 49144	Gm49144
52,93	2,15	0,76	5.366039e-04	RAB30, member RAS oncogene family	Rab30
588,89	2,11	0,35	2.863217e-10	predicted gene, 48416	Gm48416
56,44	2,06	0,55	2.267479e-05	predicted gene, 47853	Gm47853
54,78	2,06	0,57	3.751345e-05	vomeronasal 2, receptor 112	Vmn2r112
1001,39	2,04	0,30	3.690777e-12	predicted gene, 48415	Gm48415
403,41	2,04	0,33	8.649617e-11	predicted gene, 47821	Gm47821
26,25	2,04	1,01	6.783442e-03	olfactory receptor 1487	Olfr1487
85,89	2,02	0,51	1.061935e-05	predicted gene, 47356	Gm47356
45,54	1,99	0,77	1.360031e-03	ubiquitin specific peptidase 33	Usp33
38,45	1,99	0,83	2.348892e-03	nebulette	Nebl
37,17	1,98	0,70	5.035690e-04	glycine receptor, beta subunit	Glrb
28,20	1,96	0,86	3.004450e-03	predicted gene 4951	Gm4951
99,79	1,96	0,92	4.737146e-03	thrombospondin, type I, domain containing 7A	Thsd7a
36,29	1,94	0,71	7.075010e-04	olfactory receptor 699	Olfr699
330,24	1,94	0,32	1.894421e-10	predicted gene, 48054	Gm48054
32,41	1,94	0,85	3.063311e-03	APOBEC1 complementation factor	A1cf
37,33	1,92	0,92	5.485007e-03	predicted gene, 49795	Gm49795
139,97	1,92	0,46	4.313872e-06	predicted gene 43540	Gm43540
104,15	1,89	0,57	9.745002e-05	predicted gene, 28010	Gm28010
22,63	1,89	0,98	8.412433e-03	RIKEN cDNA E030022I16 gene	E030022I16Rik
41,23	1,87	0,92	6.413469e-03	RIKEN cDNA 4933427J07 gene	4933427J07Rik
65,41	1,84	0,59	2.116906e-04	predicted gene, 49165	Gm49165
83,90	1,84	0,75	1.971746e-03	cytidine monophosphate (UMP-CMP) kinase 2, mitochondrial	Cmpk2
392,25	1,83	0,32	1.200675e-09	predicted gene 43498	Gm43498
367,63	1,78	0,44	8.695002e-06	predicted gene 17131	Gm17131
26,78	1,76	0,93	9.207264e-03	predicted gene, 44078	Gm44078
47,97	1,75	0,69	1.505901e-03	xylosyltransferase 1	Xylt1
63,62	1,73	0,83	5.553699e-03	centrosomal protein 85-like	Cep85l
92,95	1,73	0,70	1.932160e-03	predicted gene 28756	Gm28756
119,55	1,72	0,41	3.009799e-06	tetratricopeptide repeat domain 9C	Ttc9c
439,82	1,72	0,55	2.002547e-04	surfeit gene 6	Surf6

baseMean	log2FoldChange	lfcSE	svalue	protein names	gene names
99,70	1,72	0,55	1.946413e-04	olfactory receptor 1247	Olfr1247
113,64	1,71	0,89	8.288158e-03	sterol O-acyltransferase 1	Soat1
251,86	1,70	0,35	1.573842e-07	predicted gene, 47331	Gm47331
60,83	1,70	0,73	2.648216e-03	melanocortin 1 receptor	Mc1r
62,89	1,68	0,56	3.494937e-04	predicted gene, 48841	Gm48841
27,06	1,68	0,87	8.205475e-03	predicted gene, 16892	Gm16892
144,92	1,67	0,45	2.157130e-05	predicted gene, 37985	Gm37985
289,92	1,67	0,35	1.892575e-07	predicted gene, 48501	Gm48501
39,70	1,67	0,75	3.797595e-03	UDP glucuronosyltransferase 2 family, polypeptide B37	Ugt2b37
3798,28	1,67	0,28	4.121896e-10	predicted gene, 47164	Gm47164
175,67	1,66	0,62	9.632281e-04	casein kinase 1, gamma 1	Csnk1g1
251,07	1,64	0,38	2.092047e-06	predicted gene, 44189	Gm44189
195,60	1,62	0,46	4.758908e-05	centrosomal protein 57	Cep57
822,12	1,62	0,36	4.952738e-07	predicted gene, 21962	Gm21962
156,11	1,61	0,52	2.059062e-04	membrane protein, palmitoylated 7 (MAGUK p55 subfamily member 7)	Mpp7
337,04	1,61	0,37	1.215153e-06	predicted gene, 47147	Gm47147
55,69	1,61	0,72	3.562440e-03	coiled-coil domain containing 144B	Ccdc144b
198,15	1,60	0,51	2.175249e-04	predicted gene, 48270	Gm48270
196,93	1,59	0,46	5.872126e-05	GTP binding protein 2	Gtpbp2
30,64	1,59	0,84	9.338621e-03	RIKEN cDNA 4930452G13 gene	4930452G13Rik
202,55	1,58	0,35	7.822750e-07	novel transcript	AC139131.1
3028,22	1,58	0,45	4.328112e-05	heparin-binding EGF-like growth factor	Hbegf
108,18	1,58	0,45	4.118998e-05	olfactory receptor 365	Olfr365
480,33	1,57	0,37	2.529301e-06	predicted gene, 48498	Gm48498
91,30	1,57	0,78	6.859034e-03	chondroitin sulfate proteoglycan 5	Cspg5
59,90	1,57	0,60	1.133316e-03	vomeronasal 1 receptor 26	Vmn1r26
567,37	1,57	0,32	9.586338e-08	predicted gene, 47626	Gm47626
155,59	1,56	0,41	1.527940e-05	vomeronasal 1 receptor, 88	Vmn1r88
154,87	1,55	0,41	1.629944e-05	predicted gene, 47355	Gm47355
739,76	1,55	0,36	2.745804e-06	predicted gene 42785	Gm42785
61,60	1,54	0,61	1.634132e-03	non-catalytic region of tyrosine kinase adaptor protein 1	Nck1
234,04	1,53	0,44	5.636704e-05	pre-mRNA processing factor 18	Prpf18
209,23	1,53	0,44	5.180820e-05	predicted gene, 48177	Gm48177
62,67	1,53	0,66	2.728012e-03	zinc finger protein 488	Zfp488
382,80	1,53	0,48	1.625549e-04	Mir22 host gene (non-protein coding)	Mir22hg
1355,40	1,53	0,30	1.758848e-08	predicted gene, 48269	Gm48269
250,10	1,53	0,41	2.722045e-05	predicted gene, 47611	Gm47611
55,66	1,52	0,75	6.821302e-03	vomeronasal 1 receptor 76	Vmn1r76
55,61	1,52	0,70	4.472343e-03	zinc finger protein 446	Zfp446
54,86	1,51	0,60	1.524017e-03	predicted gene, 47678	Gm47678
133,02	1,51	0,46	1.054041e-04	predicted gene, 47632	Gm47632
172,58	1,50	0,49	2.817313e-04	olfactory receptor 639	Olfr639
100,43	1,50	0,57	1.182503e-03	RIKEN cDNA 4930435F18 gene	4930435F18Rik
389,78	1,50	0,30	6.330844e-08	predicted gene, 47628	Gm47628
47,01	1,49	0,73	5.977690e-03	transmembrane protein 140	Tmem140
99,29	1,49	0,44	7.577272e-05	predicted gene, 43960	Gm43960
458,07	1,49	0,35	3.306215e-06	phosphatidylinositol-4-phosphate 3-kinase catalytic subunit type 2 alpha	Pik3c2a
88,73	1,49	0,72	5.869010e-03	TATA-box binding protein associated factor 2	Taf2
79,10	1,49	0,58	1.306985e-03	predicted gene 44578	Gm44578
48,79	1,49	0,76	7.916025e-03	tubulin, gamma complex associated protein 5	Tubgcp5
555,71	1,48	0,55	9.333278e-04	sperm associated antigen 9	Spag9
323,20	1,48	0,54	7.214107e-04	interferon-induced protein with tetratricopeptide repeats 3	Ifit3
219,56	1,48	0,40	3.132436e-05	family with sequence similarity 135, member B	Fam135b
69,61	1,47	0,68	4.537337e-03	predicted gene, 37903	Gm37903
298,83	1,46	0,37	1.010952e-05	predicted gene, 17555	Gm17555
71,28	1,46	0,56	1.253404e-03	olfactory receptor 393	Olfr393
396,77	1,45	0,49	3.186678e-04	carbonic anhydrase 3	Car3
46,11	1,45	0,71	6.708616e-03	predicted gene, 47974	Gm47974
657,06	1,44	0,42	6.374821e-05	solute carrier family 12, member 1	Slc12a1
385,32	1,44	0,36	9.608531e-06	predicted gene, 48786	Gm48786
78,64	1,44	0,57	1.670980e-03	RAB23, member RAS oncogene family	Rab23
536,13	1,41	0,38	1.941926e-05	vomeronasal 2, receptor 53	Vmn2r53
655,28	1,41	0,32	9.737650e-07	olfactory receptor 1307	Olfr1307
109,20	1,41	0,58	2.182169e-03	2'-5' oligoadenylate synthetase-like 1	Oasl1
127,32	1,40	0,55	1.469288e-03	kynurenine aminotransferase 3	Kyat3
307,04	1,40	0,35	8.242150e-06	predicted gene, 48541	Gm48541
250,14	1,40	0,37	2.050195e-05	vomeronasal 1 receptor 55	Vmn1r55
489,97	1,39	0,34	5.754550e-06	predicted gene, 47933	Gm47933
112,06	1,39	0,49	5.143557e-04	excision repair cross-complementing rodent repair deficiency, complementation group 6	Ercc6
34852,26	1,38	0,44	1.781934e-04	predicted gene, 21738	Gm21738
16742,71	1,38	0,31	6.395076e-07	predicted gene 10717	Gm10717
21640,91	1,38	0,27	4.682724e-08	predicted gene 42428	Gm42428
268,66	1,37	0,48	5.254119e-04	predicted gene, 48132	Gm48132
91,66	1,37	0,51	8.334796e-04	RIKEN cDNA A130071D04 gene	A130071D04Rik
72,14	1,37	0,54	1.560791e-03	vomeronasal 2, receptor 115	Vmn2r115
134485,83	1,37	0,34	9.148272e-06	predicted gene 10719	Gm10719
660,58	1,37	0,43	1.727724e-04	solute carrier family 7, (cationic amino acid transporter, y+ system) member 13	Slc7a13
475,44	1,37	0,62	3.885483e-03	midasin AAA ATPase 1	Mdn1
122,44	1,37	0,45	2.679291e-04	HDGF like 3	Hdgfl3
178,39	1,36	0,48	5.829633e-04	predicted gene 45033	Gm45033
124,10	1,36	0,47	4.710983e-04	predicted gene, 38194	Gm38194
61,56	1,36	0,64	5.041444e-03	Ly6/Plaur domain containing 5	Lypd5
208,27	1,36	0,36	1.733066e-05	predicted gene, 47967	Gm47967
10463,36	1,35	0,31	1.437554e-06	predicted gene 11168	Gm11168
227,08	1,35	0,64	5.280325e-03	predicted gene, 44310	Gm44310
108,52	1,35	0,64	5.382303e-03	predicted gene, 23365	Gm23365
118,10	1,34	0,73	9.971523e-03	homer scaffolding protein 1	Homer1
266,86	1,33	0,39	6.953241e-05	vomeronasal 1 receptor 81	Vmn1r81
431,60	1,33	0,36	2.377932e-05	predicted gene, 47476	Gm47476
734,22	1,33	0,40	1.093340e-04	predicted gene, 48082	Gm48082
616,17	1,33	0,34	1.325827e-05	predicted gene, 37515	Gm37515
198,27	1,33	0,55	2.205177e-03	cyclin-dependent kinase 8	Cdk8
147,42	1,33	0,40	1.172785e-04	predicted gene 10660	Gm10660

baseMean	log2FoldChange	lfcSE	svalue	protein names	gene names
2441,03	1,32	0,31	2.302705e-06	predicted gene 10720	Gm10720
159,02	1,31	0,58	3.121462e-03	predicted gene, 30948	Gm30948
141,95	1,31	0,48	7.771037e-04	predicted gene, 21833	Gm21833
1608,71	1,31	0,39	9.356509e-05	heme oxygenase 1	Hmox1
310,71	1,31	0,65	7.047218e-03	non imprinted in Prader-Willi/Angelman syndrome 2 homolog (human)	Nipa2
235809,69	1,30	0,33	1.184392e-05	predicted gene 10800	Gm10800
54,19	1,30	0,68	8.914715e-03	olfactory receptor 45	Olfr45
198,12	1,30	0,40	1.295024e-04	predicted gene 44715	Gm44715
978,22	1,29	0,50	1.342383e-03	predicted gene, 48412	Gm48412
73,06	1,29	0,57	3.295832e-03	tripartite motif-containing 30A	Trim30a
314,48	1,29	0,42	2.292552e-04	predicted gene, 48216	Gm48216
2557,89	1,29	0,47	6.090775e-04	cyclin-dependent kinase 11B	Cdk11b
2680,31	1,29	0,30	1.663349e-06	predicted gene 10722	Gm10722
1013,18	1,29	0,30	1.874984e-06	predicted gene, 21897	Gm21897
64253,30	1,29	0,42	2.419108e-04	predicted gene 10718	Gm10718
1150,48	1,29	0,34	1.838738e-05	predicted gene, 17535	Gm17535
92,19	1,29	0,48	9.785713e-04	predicted gene, 48366	Gm48366
181,62	1,28	0,42	3.031668e-04	predicted gene 43294	Gm43294
95,44	1,28	0,51	1.763278e-03	predicted gene 6211	Gm6211
190,72	1,28	0,49	1.235526e-03	predicted gene, 47932	Gm47932
44720,47	1,27	0,31	6.134864e-06	predicted gene 10801	Gm10801
174,26	1,27	0,38	8.617068e-05	predicted gene, 47968	Gm47968
88,12	1,27	0,58	4.093689e-03	predicted gene, 47842	Gm47842
207,64	1,26	0,49	1.289088e-03	predicted gene, 48401	Gm48401
30692,88	1,26	0,40	1.573227e-04	predicted gene 10721	Gm10721
188,58	1,25	0,39	1.337097e-04	predicted gene, 48418	Gm48418
230,83	1,25	0,46	9.040135e-04	RHO family interacting cell polarization regulator 2	Ripor2
251,33	1,25	0,36	6.109879e-05	predicted gene, 48793	Gm48793
235,65	1,24	0,61	6.450488e-03	interferon-induced protein with tetratricopeptide repeats 1	Ifit1
245,28	1,24	0,45	6.509423e-04	predicted gene, 38297	Gm38297
107,71	1,24	0,51	2.034062e-03	vomeronasal 2, receptor 97	Vmn2r97
249,96	1,23	0,43	4.502720e-04	TBC1 domain family, member 32	Tbc1d32
79,15	1,22	0,61	7.009706e-03	cell division cycle 25C	Cdc25c
88,06	1,22	0,54	3.532880e-03	predicted gene 44848	Gm44848
183,70	1,22	0,37	1.212334e-04	vomeronasal 2, receptor 59	Vmn2r59
324,44	1,22	0,43	4.926640e-04	predicted gene 45053	Gm45053
906,79	1,22	0,35	4.969016e-05	predicted gene 10715	Gm10715
527,31	1,21	0,59	6.195144e-03	predicted gene, 21596	Gm21596
394,77	1,21	0,36	7.910477e-05	predicted gene, 48443	Gm48443
658,60	1,21	0,32	2.488809e-05	olfactory receptor 46	Olfr46
256,28	1,20	0,34	4.545927e-05	predicted gene, 47056	Gm47056
122,30	1,20	0,53	3.325193e-03	predicted gene, 37404	Gm37404
237,94	1,19	0,42	5.481079e-04	protein phosphatase 1, regulatory inhibitor subunit 1A	Ppp1r1a
3037,97	1,19	0,44	8.054667e-04	bromodomain containing 4	Brd4
133,31	1,18	0,47	1.708011e-03	predicted gene, 49902	Gm49902
93,25	1,18	0,55	4.636478e-03	vomeronasal 1 receptor 236	Vmn1r236
211,18	1,18	0,61	8.246886e-03	structural maintenance of chromosomes 4	Smc4
100,50	1,17	0,54	4.187447e-03	RIKEN cDNA A330044P14 gene	A330044P14Rik
203,12	1,17	0,60	8.122462e-03	SPT20 SAGA complex component	Supt20
69,36	1,17	0,56	5.727441e-03	predicted gene 42546	Gm42546
419,09	1,16	0,58	7.196501e-03	karyopherin (importin) alpha 1	Kpna1
246,13	1,16	0,40	3.878970e-04	olfactory receptor 635	Olfr635
166,27	1,16	0,52	3.768137e-03	vomeronasal 1 receptor 227	Vmn1r227
14622,74	1,15	0,28	3.980872e-06	nuclear paraspeckle assembly transcript 1 (non-protein coding)	Neat1
437,49	1,14	0,33	5.403156e-05	predicted gene, 21750	Gm21750
436,85	1,14	0,39	4.191373e-04	LIM domain only 7	Lmo7
372,16	1,13	0,38	2.958300e-04	predicted gene 42669	Gm42669
1878,76	1,13	0,41	7.351707e-04	CREB binding protein	Crebbp
103,72	1,13	0,57	7.272724e-03	centrosomal protein 135	Cep135
1360,95	1,13	0,38	3.339689e-04	calpastatin	Cast
124,58	1,12	0,55	6.598367e-03	RIKEN cDNA 2900027M19 gene	2900027M19Rik
600,72	1,12	0,46	2.097202e-03	predicted gene 45178	Gm45178
136,77	1,11	0,52	4.504612e-03	zinc finger protein 280D	Zfp280d
196,54	1,11	0,52	4.973434e-03	RIKEN cDNA 4631422I05 gene	4631422I05Rik
135,52	1,11	0,43	1.395998e-03	predicted gene 43362	Gm43362
85,35	1,10	0,58	9.039363e-03	MACRO domain containing 1	Macrod1
141,26	1,10	0,52	4.905289e-03	predicted gene, 36839	Gm36839
181,80	1,10	0,48	2.862824e-03	zinc finger protein 574	Zfp574
205,48	1,09	0,45	2.228077e-03	predicted gene, 38451	Gm38451
1755,16	1,09	0,28	1.255187e-05	predicted gene 17132	Gm17132
70,62	1,09	0,56	7.751820e-03	SAS-6 centriolar assembly protein	Sass6
166,17	1,09	0,50	4.312598e-03	myeloid/lymphoid or mixed-lineage leukemia; translocated to, 10	Mllt10
129,18	1,08	0,53	6.635238e-03	trans-acting transcription factor 3	Sp3
9201,38	1,08	0,45	2.397136e-03	predicted gene, 25072	Gm25072
137,83	1,08	0,44	1.992538e-03	predicted gene, 48260	Gm48260
182,22	1,07	0,38	5.596209e-04	predicted gene, 37086	Gm37086
263,36	1,07	0,37	4.087942e-04	transcription factor A, mitochondrial	Tfam
1312,95	1,07	0,29	2.997968e-05	predicted gene, 48119	Gm48119
1375,48	1,07	0,36	3.263668e-04	scaffold attachment factor B2	Safb2
77,29	1,06	0,54	7.670884e-03	predicted gene, 47022	Gm47022
134,00	1,06	0,50	5.416684e-03	retinol dehydrogenase 12	Rdh12
328,61	1,06	0,54	7.510409e-03	vacuolar protein sorting 13D	Vps13d
1037,33	1,05	0,48	4.408311e-03	trinucleotide repeat containing 6a	Tnrc6a
1372,79	1,03	0,31	8.246953e-05	RAR-related orphan receptor alpha	Rora
443,39	1,03	0,31	1.014467e-04	predicted gene, 47654	Gm47654
205,29	1,03	0,51	7.234441e-03	predicted gene, 18214	Gm18214
176,80	1,03	0,45	3.150545e-03	predicted gene 13722	Gm13722
179,92	1,02	0,46	3.856200e-03	Rho GTPase activating protein 32	Arhgap32
1035,54	1,02	0,46	3.709633e-03	WAP four-disulfide core domain 15B	Wfdc15b
511,28	1,02	0,33	2.614729e-04	CREB3 regulatory factor	Crebrf
134,28	1,01	0,50	6.524469e-03	ubiquitin protein ligase E3 component n-recognin 1	Ubr1
211,84	1,01	0,50	6.561453e-03	predicted gene 10408	Gm10408
788,53	1,01	0,30	7.248908e-05	mitogen-activated protein kinase kinase kinase 5	Map3k5

baseMean	log2FoldChange	lfcSE	svalue	protein names	gene names
211,84	1,01	0,50	6.561453e-03	predicted gene 10408	Gm10408
156,79	1,00	0,52	8.831447e-03	predicted gene 43042	Gm43042
292,85	1,00	0,43	2.807778e-03	protein disulfide isomerase associated 4	Pdia4
1918,43	1,00	0,42	2.324846e-03	predicted gene, 50383	Gm50383
304,09	0,99	0,40	1.874772e-03	olfactory receptor 338	Olfr338
166,89	0,99	0,50	7.430407e-03	single-stranded DNA binding protein 2	Ssbp2
217,73	0,99	0,43	2.780783e-03	C2 calcium-dependent domain containing 5	C2cd5
730,24	0,98	0,30	1.380118e-04	predicted gene, 37954	Gm37954
146,40	0,98	0,52	9.563243e-03	predicted gene 43088	Gm43088
1355,18	0,98	0,37	9.937911e-04	predicted gene, 48309	Gm48309
947,19	0,97	0,44	3.943628e-03	predicted gene 42519	Gm42519
555,40	0,97	0,35	6.369781e-04	dishevelled associated activator of morphogenesis 1	Daam1
311,60	0,97	0,38	1.432629e-03	triple functional domain (PTPRF interacting)	Trio
308,64	0,96	0,36	1.070308e-03	vomeronasal 2, receptor 76	Vmn2r76
3509,01	0,96	0,29	1.252239e-04	aldo-keto reductase family 1, member C21	Akr1c21
512,29	0,96	0,36	1.117532e-03	predicted gene, 49534	Gm49534
131,42	0,96	0,50	9.123561e-03	predicted gene, 31258	Gm31258
261,11	0,96	0,46	6.013969e-03	solute carrier family 9 (sodium/hydrogen exchanger), member 3	Slc9a3
272,45	0,96	0,38	1.652620e-03	predicted gene 15481	Gm15481
718,63	0,95	0,41	2.621998e-03	predicted gene 13577	Gm13577
1801,73	0,95	0,51	9.653054e-03	N(alpha)-acetyltransferase 15, NatA auxiliary subunit	Naa15
1333,41	0,94	0,31	2.886880e-04	ring finger protein 149	Rnf149
940,50	0,94	0,40	2.470386e-03	predicted gene, 47578	Gm47578
1635,42	0,94	0,36	1.149533e-03	Rho-associated coiled-coil containing protein kinase 2	Rock2
213,78	0,94	0,42	3.738950e-03	olfactory receptor 344	Olfr344
559,38	0,93	0,45	5.904924e-03	predicted gene, 35206	Gm35206
480,31	0,93	0,41	3.354774e-03	cystatin domain containing 4	Cstdc4
321,20	0,93	0,41	3.384595e-03	RIKEN cDNA 9930017N22 gene	9930017N22Rik
139,73	0,93	0,49	8.705637e-03	predicted gene 13684	Gm13684
1105,15	0,92	0,38	2.139833e-03	aspartoacylase (aminoacylase) 3	Acy3
780,06	0,91	0,45	6.671967e-03	membrane protein, palmitoylated 5 (MAGUK p55 subfamily member 5)	Mpp5
1636,35	0,90	0,47	8.956150e-03	Eph receptor A5	Epha5
296,99	0,89	0,33	7.629660e-04	acyl-Coenzyme A binding domain containing 5	Acbd5
1682,10	0,89	0,42	5.450896e-03	DEAH (Asp-Glu-Ala-His) box polypeptide 15	Dhx15
694,35	0,87	0,41	4.669984e-03	ring finger and CCCH-type zinc finger domains 2	Rc3h2
515,52	0,87	0,45	8.538109e-03	FYVE, RhoGEF and PH domain containing 4	Fgd4
509,19	0,87	0,46	9.081527e-03	microtubule-associated protein 7	Map7
434,70	0,87	0,37	2.421590e-03	ELKS/RAB6-interacting/CAST family member 1	Erc1
383,73	0,86	0,42	6.487416e-03	protein kinase N2	Pkn2
972,11	0,86	0,40	4.376343e-03	natural killer tumor recognition sequence	Nktr
623,28	0,86	0,36	2.519193e-03	topoisomerase I binding, arginine/serine-rich	Topors
5304,97	0,86	0,30	5.712762e-04	eukaryotic translation initiation factor 3, subunit A	Eif3a
224,53	0,85	0,37	2.917652e-03	RIKEN cDNA A630089N07 gene	A630089N07Rik
449,15	0,85	0,34	1.912551e-03	jumonji domain containing 1C	Jmjd1c
142,64	0,85	0,44	8.579977e-03	major facilitator superfamily domain containing 4A	Mfsd4a
190,05	0,84	0,40	4.804592e-03	predicted gene, 17477	Gm17477
310,11	0,84	0,36	2.835287e-03	protein-L-isoaspartate (D-aspartate) O-methyltransferase domain containing 1	Pcmtd1
507,85	0,84	0,33	1.597148e-03	microtubule associated serine/threonine kinase family member 4	Mast4
1363,13	0,84	0,36	2.890178e-03	striatin, calmodulin binding protein 3	Strn3
426,84	0,82	0,33	1.744985e-03	RIKEN cDNA B930086L07 gene	B930086L07Rik
1799,91	0,82	0,36	3.237556e-03	predicted gene 43305	Gm43305
1133,42	0,81	0,39	5.657436e-03	methyl-CpG binding domain protein 4	Mbd4
277,30	0,81	0,44	9.742531e-03	B double prime 1, subunit of RNA polymerase III transcription initiation factor IIIB	Bdp1
374,48	0,80	0,41	7.957366e-03	coiled-coil domain containing 138	Ccdc138
283,68	0,79	0,38	5.622579e-03	solute carrier family 22, member 23	Slc22a23
201,38	0,79	0,40	7.630503e-03	MER proto-oncogene tyrosine kinase	Mertk
594,75	0,78	0,36	4.570136e-03	RIO kinase 1	Riok1
729,33	0,78	0,40	8.496155e-03	major facilitator superfamily domain containing 4B5	Mfsd4b5
484,21	0,77	0,36	4.440165e-03	phosphoribosylformylglycinamidine synthase (FGAR amidotransferase)	Pfas
413,07	0,77	0,38	6.746004e-03	vomeronasal 2, receptor 114	Vmn2r114
882,52	0,75	0,38	7.711118e-03	Luc7-like	Luc7l
263,94	0,75	0,35	5.007443e-03	B-TFIID TATA-box binding protein associated factor 1	Btaf1
430,76	0,74	0,33	3.033960e-03	solute carrier family 1 (neutral amino acid transporter), member 5	Slc1a5
1189,43	0,74	0,38	7.874716e-03	polypyrimidine tract binding protein 3	Ptbp3
255,01	0,74	0,35	5.212080e-03	RIKEN cDNA 9930111J21 gene 2	9930111J21Rik2
834,45	0,74	0,32	2.544762e-03	solute carrier family 22 (organic cation transporter), member 2	Slc22a2
265,11	0,73	0,36	7.159397e-03	mitochondrial assembly of ribosomal large subunit 1	Malsu1
541,48	0,73	0,39	9.608235e-03	cytochrome P450, family 2, subfamily j, polypeptide 13	Cyp2j13
530,85	0,73	0,34	5.144091e-03	predicted gene, 49971	Gm49971
631,09	0,72	0,30	2.300824e-03	pyruvate dehydrogenase kinase, isoenzyme 2	Pdk2
505,31	0,71	0,38	9.294671e-03	polycomb group ring finger 5	Pcgf5
1851,42	0,71	0,38	9.427593e-03	PRP38 pre-mRNA processing factor 38 (yeast) domain containing B	Prpf38b
570,09	0,70	0,30	2.945862e-03	mitochondrial ribosomal protein L48	Mrpl48
441,47	0,69	0,34	6.340483e-03	zinc finger protein 326	Zfp326
902,97	0,68	0,32	5.314344e-03	KCNQ1 overlapping transcript 1	Kcnq1ot1
3481,16	0,68	0,34	6.934560e-03	defensin beta 1	Defb1
680,26	0,67	0,34	7.550354e-03	sphingomyelin synthase 1	Sgms1
761,76	0,65	0,30	4.002265e-03	ring finger protein 168	Rnf168
703,38	0,65	0,34	8.370862e-03	kelch-like 41	Klhl41
301,73	0,65	0,33	7.998567e-03	predicted gene 43622	Gm43622
603,80	0,64	0,33	7.390815e-03	protein phosphatase 4, regulatory subunit 2	Ppp4r2
755,87	0,62	0,33	9.382902e-03	ankyrin repeat domain 12	Ankrd12
1115,62	0,58	0,31	9.831827e-03	MOB family member 4, phocein	Mob4
1049,45	0,55	0,28	7.833384e-03	Rho guanine nucleotide exchange factor (GEF) 12	Arhgef12
1490,39	0,51	0,27	8.621711e-03	WD repeat domain, phosphoinositide interacting 2	Wipi2
4041,42	-0,48	0,25	9.165476e-03	Y box protein 1	Ybx1
6765,87	-0,50	0,26	8.164020e-03	esterase D/formylglutathione hydrolase	Esd
2344,17	-0,52	0,27	7.792569e-03	ribosomal protein L36A-like	Rpl36al
1345,18	-0,53	0,27	7.590470e-03	proteasome (prosome, macropain) subunit, beta type 6	Psmb6
5996,27	-0,53	0,26	5.833335e-03	ribosomal protein S5	Rps5
550,95	-0,55	0,30	9.877559e-03	polymerase (RNA) II (DNA directed) polypeptide K	Polr2k
3589,46	-0,56	0,30	9.250816e-03	chloride intracellular channel 1	Clic1

baseMean	log2FoldChange	lfcSE	svalue	protein names	gene names
2265,54	-0,57	0,31	9.924610e-03	heterogeneous nuclear ribonucleoprotein A/B	Hnrnpab
1853,31	-0,57	0,29	8.039668e-03	tubulin, beta 2A class IIA	Tubb2a
2087,57	-0,58	0,30	8.663391e-03	retinoblastoma binding protein 4, chromatin remodeling factor	Rbbp4
1253,53	-0,59	0,31	8.789665e-03	predicted gene, 26619	Gm26619
1618,78	-0,59	0,29	6.267587e-03	myosin, light chain 12A, regulatory, non-sarcomeric	Myl12a
939,96	-0,60	0,30	6.896876e-03	karyopherin (importin) beta 1	Kpnb1
3609,53	-0,60	0,29	6.050174e-03	ribosomal protein S6	Rps6
2105,24	-0,61	0,27	3.473699e-03	RAN, member RAS oncogene family	Ran
1094,67	-0,61	0,29	5.178058e-03	mitochondrial ribosomal protein L33	Mrpl33
1295,66	-0,62	0,33	9.518097e-03	ring-box 1	Rbx1
2681,58	-0,62	0,27	2.596130e-03	thioredoxin 1	Txn1
911,87	-0,62	0,32	8.080963e-03	RIKEN cDNA B020010K11 gene	B020010K11Rik
6594,33	-0,63	0,31	5.941354e-03	glutaredoxin	Glrx
554,67	-0,64	0,32	7.084701e-03	NudC domain containing 3	Nudcd3
5239,21	-0,65	0,28	2.373087e-03	ribosomal protein L17	Rpl17
523,16	-0,65	0,31	5.109934e-03	PRELI domain containing 3B	Prelid3b
464,85	-0,66	0,31	4.603171e-03	annexin A4	Anxa4
4873,13	-0,67	0,27	1.818583e-03	phosphogluconate dehydrogenase	Pgd
326,33	-0,68	0,36	8.747723e-03	proliferation-associated 2G4	Pa2g4
511,02	-0,69	0,33	6.122616e-03	serine (or cysteine) peptidase inhibitor, clade H, member 1	Serpinh1
542,66	-0,70	0,31	3.592130e-03	lectin, mannose-binding 2	Lman2
16209,02	-0,70	0,26	8.474226e-04	peroxiredoxin 1	Prdx1
933,98	-0,71	0,30	2.446077e-03	predicted gene, 17300	Gm17300
1264,65	-0,71	0,27	1.085869e-03	RAB10, member RAS oncogene family	Rab10
313,42	-0,73	0,33	4.124936e-03	ATP-binding cassette, sub-family F (GCN20), member 2	Abcf2
482,39	-0,73	0,30	2.013299e-03	regulator of chromosome condensation 2	Rcc2
328,70	-0,73	0,34	5.246290e-03	EBNA1 binding protein 2	Ebna1bp2
364,72	-0,74	0,33	3.266595e-03	refilin B	Rflnb
1282,89	-0,75	0,36	5.692346e-03	microsomal glutathione S-transferase 1	Mgst1
922,26	-0,75	0,28	1.024204e-03	adaptor protein complex AP-1, sigma 1	Ap1s1
257,82	-0,75	0,34	4.281175e-03	tripartite motif-containing 35	Trim35
2561,62	-0,75	0,28	8.895947e-04	quiescin Q6 sulfhydryl oxidase 1	Qsox1
304,33	-0,76	0,37	6.231336e-03	3-hydroxyacyl-CoA dehydratase 1	Hacd1
1931,70	-0,77	0,35	4.063137e-03	A kinase (PRKA) anchor protein (gravin) 12	Akap12
364,07	-0,77	0,36	4.939236e-03	mitochondrial ribosomal protein L50	Mrpl50
277,51	-0,77	0,39	7.470366e-03	homeobox C10	Hoxc10
271,41	-0,78	0,35	3.651068e-03	WD repeat domain 54	Wdr54
219,40	-0,79	0,39	6.376980e-03	Ras converting CAAX endopeptidase 1	Rce1
769,20	-0,80	0,33	2.160969e-03	expressed sequence AI413582	AI413582
180,31	-0,80	0,40	7.311765e-03	Fas apoptotic inhibitory molecule	Faim
316,25	-0,81	0,34	2.252328e-03	CLPTM1-like	Clptm1l
264,99	-0,81	0,37	4.344337e-03	phosducin-like 3	Pdcl3
523,78	-0,81	0,37	4.156204e-03	serine/arginine-rich splicing factor 9	Srsf9
1495,12	-0,81	0,31	1.054921e-03	cystatin B	Cstb
220,30	-0,83	0,43	8.873127e-03	small integral membrane protein 10 like 1	Smim10l1
226,94	-0,85	0,39	4.250017e-03	Terf1 (TRF1)-interacting nuclear factor 2	Tinf2
224,53	-0,85	0,40	4.770897e-03	WASH complex subunit 1	Washc1
186,19	-0,85	0,44	8.997578e-03	c-src tyrosine kinase	Csk
260,18	-0,86	0,35	2.054750e-03	WT1-interacting protein	Wtip
347,04	-0,86	0,34	1.781531e-03	bridging integrator 1	Bin1
169,04	-0,87	0,45	8.454288e-03	small integral membrane protein 11	Smim11
143,34	-0,88	0,47	9.472919e-03	DnaJ heat shock protein family (Hsp40) member A3	Dnaja3
1432,37	-0,89	0,29	2.484768e-04	RIKEN cDNA 2200002D01 gene	2200002D01Rik
216,04	-0,89	0,48	9.787150e-03	guanosine monophosphate reductase	Gmpr
267,09	-0,89	0,39	3.179485e-03	transcription termination factor, RNA polymerase I	Ttf1
1411,44	-0,90	0,36	1.837369e-03	tubulin, beta 5 class I	Tubb5
251,88	-0,90	0,39	2.674738e-03	RIKEN cDNA 2210016L21 gene	2210016L21Rik
598,98	-0,90	0,35	1.324720e-03	dolichol-phosphate (beta-D) mannosyltransferase 2	Dpm2
622,67	-0,91	0,32	4.398517e-04	RIKEN cDNA 2610001J05 gene	2610001J05Rik
5498,03	-0,91	0,35	1.199710e-03	sulfiredoxin 1 homolog (S. cerevisiae)	Srxn1
379,94	-0,92	0,35	1.039506e-03	six transmembrane epithelial antigen of the prostate 1	Steap1
1228,77	-0,95	0,38	1.799992e-03	S100 calcium binding protein A6 (calcyclin)	S100a6
225,27	-0,95	0,43	3.914625e-03	abhydrolase domain containing 4	Abhd4
3566,83	-0,96	0,33	3.983730e-04	glutathione reductase	Gsr
428,01	-1,03	0,41	1.689552e-03	cytochrome C oxidase assembly factor 3	Coa3
322,00	-1,03	0,40	1.217596e-03	RIKEN cDNA 1300002E11 gene	1300002E11Rik
142,61	-1,03	0,55	9.697859e-03	dihydropyrimidinase-like 2	Dpysl2
726,51	-1,04	0,33	1.520073e-04	phosphoserine aminotransferase 1	Psat1
712,22	-1,06	0,29	3.595125e-05	predicted gene, 17711	Gm17711
383,06	-1,06	0,45	2.276666e-03	trafficking protein particle complex 1	Trappc1
346,41	-1,10	0,49	3.826929e-03	Ral GTPase activating protein, beta subunit (non-catalytic)	Ralgapb
154,36	-1,11	0,41	7.913115e-04	block of proliferation 1	Bop1
851,35	-1,12	0,38	3.581252e-04	activating transcription factor 5	Atf5
587,71	-1,12	0,29	1.428334e-05	glia maturation factor, beta	Gmfb
101,26	-1,14	0,50	3.208535e-03	retinol dehydrogenase 11	Rdh11
452,34	-1,17	0,51	3.092477e-03	aldo-keto reductase family 1, member B8	Akr1b8
1173,89	-1,18	0,32	2.603081e-05	profilin 1	Pfn1
329,17	-1,18	0,37	1.677020e-04	armadillo repeat gene deleted in velocardiofacial syndrome	Arvcf
195,02	-1,18	0,43	7.490274e-04	zinc binding alcohol dehydrogenase, domain containing 2	Zadh2
85,11	-1,20	0,59	6.158872e-03	solute carrier family 14 (urea transporter), member 1	Slc14a1
243,77	-1,20	0,43	5.953152e-04	aldo-keto reductase family 1, member B3 (aldose reductase)	Akr1b3
184,18	-1,20	0,58	5.762428e-03	fibulin 5	Fbln5
199,42	-1,23	0,39	1.890763e-04	peptidylprolyl isomerase (cyclophilin)-like 1	Ppil1
209,43	-1,24	0,60	6.086414e-03	neurolysin (metallopeptidase M3 family)	Nln
81,20	-1,25	0,62	7.122129e-03	tubulin tyrosine ligase-like family, member 12	Ttll12
262,54	-1,30	0,42	2.353249e-04	BCL2-associated agonist of cell death	Bad
77,05	-1,30	0,51	1.451039e-03	EEF1A lysine methyltransferase 4	Eef1akmt4
78,45	-1,34	0,58	2.754480e-03	Hoxa transcript antisense RNA, myeloid-specific 1	Hotairm1
81,22	-1,39	0,67	5.588229e-03	coiled-coil domain containing 58	Ccdc58
95,72	-1,40	0,52	8.195425e-04	STIM activating enhancer	Stimate
100,58	-1,46	0,62	2.570539e-03	transmembrane protein 237	Tmem237
226,11	-1,47	0,46	1.469953e-04	methylthioribose-1-phosphate isomerase 1	Mri1

baseMean	log2FoldChange	lfcSE	svalue	protein names	gene names
123,58	-1,49	0,59	1.542428e-03	predicted gene, 50387	Gm50387
295,08	-1,49	0,47	1.836023e-04	predicted gene, 49416	Gm49416
352,25	-1,51	0,36	4.652629e-06	carbonyl reductase 1	Cbr1
145,49	-1,56	0,64	2.075741e-03	predicted gene, 42067	Gm42067
405,15	-1,57	0,62	1.414191e-03	solute carrier family 14 (urea transporter), member 2	Slc14a2
61,37	-1,62	0,69	2.494564e-03	centrin 4	Cetn4
130,22	-1,64	0,73	3.680364e-03	acidic (leucine-rich) nuclear phosphoprotein 32 family, member A	Anp32a
51,39	-1,65	0,85	8.329291e-03	RIKEN cDNA A730063M14 gene	A730063M14Rik
42,72	-1,67	0,67	1.726575e-03	prolyl 3-hydroxylase family member 4 (non-enzymatic)	P3h4
73,93	-1,70	0,66	1.271178e-03	tryptophan rich basic protein	Wrb
119,82	-1,76	0,49	3.436545e-05	coiled-coil domain containing 93	Ccdc93
69,03	-1,78	0,84	5.348383e-03	KN motif and ankyrin repeat domains 3	Kank3
41,69	-1,88	0,92	6.304040e-03	RIKEN cDNA B230377A18 gene	B230377A18Rik
131,11	-1,91	0,73	1.165689e-03	cytoplasmic polyadenylation element binding protein 2	Cpeb2
23,58	-1,97	0,94	5.519120e-03	RIKEN cDNA 4930522L14 gene	4930522L14Rik
21,12	-2,10	1,01	5.797670e-03	collagen, type V, alpha 1	Col5a1
33,55	-2,42	0,92	1.101639e-03	Fanconi anemia, complementation group A	Fanca
35,99	-2,74	1,00	6.651701e-04	predicted gene, 22063	Gm22063
32,73	-3,27	0,83	1.112949e-05	expressed sequence AU021092	AU021092
38,54	-3,34	1,07	2.233135e-04	RIKEN cDNA 2500002B13 gene	2500002B13Rik
59,52	-3,53	1,09	1.424709e-04	myocardial zonula adherens protein	Myzap
23,93	-3,87	1,34	4.294072e-04	RIKEN cDNA 1810026B05 gene	1810026B05Rik
35,16	-4,45	0,99	3.477858e-07	predicted gene, 23472	Gm23472
32,15	-5,42	1,30	3.636419e-06	harakiri, BCL2 interacting protein (contains only BH3 domain)	Hrk

Column name	Description
baseMean	The average of the normalized count values, dividing by size factors, taken over all samples.
log2FoldChange	The effect size estimate. This value indicates how much the gene or transcript's expression seems to have changed between the comparison and control groups. This value is reported on a logarithmic scale to base 2.
lfcSE	The standard error estimate for the log2 fold change estimate.
svalue	the negative log of the P-value $-\log_2(p)$
Protein names	Name(s) of protein(s) contained within the group.
Gene names	Name(s) of the gene(s) associated to the protein(s) contained within the group.

Supplementary Methods

Flow cytometry
For determination of neutrophil surface expression, the following antibodies were used: Gr-1 Alexa633 (selfmade), CD45 (clone 30-F11, Biolegend, #103132), Ly6B.2 (clone 7/4, Bio-Rad, #MCA771G), CD11a (clone M7/14, Biolegend, #101107), CD11b (clone M1/70, BD Bioscience, #553311), CD62L (clone MEL-14, BD Bioscience, #561918), CXCR2 (clone SA044G4, Biolegend, #149303), CD162 (clone 2PH1, BD Bioscience, #555306). Isotype controls, fluorescence-minus-one and empty staining were used to adjust threshold.
For detection of intrarenal macrophages, T-cells and dendritic cells (DCs), kidneys were flushed and digested as described above and the following antibodies were used for fluorescence-based cytometric detection: CD3 (clone 17A2, Biolegend, #100221), CD4 (clone GK1.5, Biolegend, #100431), CD8 (clone 53-6.7, Invitrogen, #11-0081-85), CD25 (clone 3C7, Biolegend, #101903), CD45 (clone 30-F11, BD Bioscience, #557235), MerTK AF488 (clone DS5MMER, Invitrogen, #53-5751-82), CD86 (clone GL-1, Biolegend, #105007), F4/80 (clone BM8, Biolegend, #123121), CD206 (clone MR6F3, Invitrogen, #25-2061-82), MHCII (clone M5/114.15.2, Invitrogen, #47-5321-82), CD11c (clone HL3, BD Bioscience, #553802). Isotype and negative controls were used to adjust threshold. A microbead-based volumetric reference was utilized for absolute quantification.

Glutamine measurement
Glutamine concentrations in urine and plasma samples were measured using a colorimetric assay (Glutamine Assay Kit, abcam, #ab197011) following the manufacturer's directions.

Immunohistochemistry
For immunohistochemistry staining sections were dewaxed by heating them to 60°C for 1 h. The paraffin was removed by processing the slices through an alcohol series [xylene (Sigma) for 10 min, 100% ethanol (Sigma) for 8 min, 90% ethanol for 2 min, 70% ethanol for 2 min]. Next, the sections were treated in 3 % H2O2 (Sigma) in ddH2O at RT to exhaust unspecific peroxidase activity. The sections were washed twice in ddH2O and antigen retrieval was performed in 1 mM citric acid (PH=6) heated to 80°C for 15 min and dried for 10 min. Next, the sections were washed in ddH2O for 2 min, blocked in TBS (PH=7.6) + 2% BSA (Sigma) for 10 min at RT and stained using primary antibodies [rb-anti-HSP70 (CST; #4872; 1:50), rb-anti-tgm2 (CST; #3557; 1:50), rb-anti-14-3-3zeta (Invitrogen; #PA5-27317; 1:500), rb-anti-Bad (Invitrogen, #MA-31978; 1:50)] at 8°C over night. Next, the sections were washed three times in TBS + 0,05 % tween20 (Merck) and stained with the secondary antibody at RT for 1h (anti-rb-HRP; CST; #7074; 1:100). The sections were again washed three times in TBS + 0,05 % tween20 and the oxidative reaction was performed using the signal stain DAB substrate kit (CST; #8059) strictly according to the manufacturer's instructions. Incubation times were 30 min for HSP70, 15 min for tgm2, 1 min for 14-3-3zeta and 4 min for Bad stained sections. The reaction was stopped in ddH2O for 5 min. Hematoxylin counterstaining was performed after dipping the sections in tap water and applying Mayer's Hämalaun solution (Carl Roth) for 8 min, followed by a wash in tap water for 30 s. Differentiation was performed by dipping the slices in ddH2O 6 times and by incubating them for 10 min in running tap water. Finally, the sections were processed in an alcohol series (70 % ethanol for 5 min, 90 % ethanol for 5 min, 100 % ethanol for 10 min, xylene for 20 min) and embedded using Eukitt (Sigma).

Viability Assay
Murine primary tubular epithelial cells (TECs) were seeded in 12-well cell culture plates and grown to confluence. Cells were subjected to glutamine or saline and DMSO or ERW1041E for 90 min (37 °C, 5% CO_2). Cell viability of TECs was tested by using the Pacific Blue™ Annexin V Apoptosis Detection Kit with PI (Biolegend, #640928) according to the manufacturer's instructions. Cells were analysed with a BD FACSCanto™ II Flow Cytometry System. Annexin V-only positive cells were considered early apoptotic, while Annexin V - PI double positive and PI-only positive cells were defined as late-stage apoptotic cells and necrotic cells.

Graphical Abstract: Glutamine reduces apoptotic Ask1-JNK signaling. Left: Renal Ischemia reperfusion leads to inflammation and the generation of ROS and mROS in renal TECs. Redox-sensitive ASK1 (apoptosis signal-regulating kinase 1) gets phosphorylated (Thr845) which causes JNK (c-Jun N-terminal kinase) activation (Ser73). 14-3-3 (Thr232) and pro-apoptotic Bad (S128) were phosphorylated which prevents an interaction. Thereby, Bad can bind to antiapoptotic Bcl-2 which blocks its function to prevent apoptosis by controlling the mitochondrial outer membrane permeabilization. Cytochrome c gets released which activates caspase 3, finally resulting in apoptosis. Right: Glutamine reduces (m)ROS production as well as TNFα release, which leads to reduced Ask1 phosphorylation and a subsequent decreased JNK activation. Moreover, glutamine treatment maintains Tgm2 and HSP70 protein expression. The signalosome of these two proteins additionally decreased JNK phosphorylation, increases Bad phosphorylation (Ser136) thereby reducing further downstream intrinsic apoptotic signaling. Short upward arrows symbolize up-regulated proteins and phosphorylation, while short downward arrows symbolize the downregulation. Solid lines symbolize direct action, while dotted lines symbolize direct interactions. Red arrows describe the effects of ischemia reperfusion as well as TNFα or hypoxia stimulation. Green arrows highlight the effects of glutamine on the single proteins.
